# Sorting‐free metabolic profiling uncovers the vulnerability of fatty acid β‐oxidation in *in vitro* quiescence models

**DOI:** 10.15252/msb.202110716

**Published:** 2022-09-12

**Authors:** Karin Ortmayr, Mattia Zampieri

**Affiliations:** ^1^ Institute of Molecular Systems Biology, ETH Zürich Switzerland; ^2^ Division of Pharmacognosy, Department of Pharmaceutical Sciences, Faculty of Life Sciences University of Vienna Vienna Austria

**Keywords:** cellular quiescence, fatty acid oxidation, metabolic adaptation, trimetazidine, quiescence-proliferation transition, Cancer, Cell Cycle, Metabolism

## Abstract

Quiescent cancer cells are rare nondiving cells with the unique ability to evade chemotherapies and resume cell division after treatment. Despite the associated risk of cancer recurrence, how cells can reversibly switch between rapid proliferation and quiescence remains a long‐standing open question. By developing a unique methodology for the cell sorting‐free separation of metabolic profiles in cell subpopulations *in vitro*, we unraveled metabolic characteristics of quiescent cells that are largely invariant to basal differences in cell types and quiescence‐inducing stimuli. Consistent with our metabolome‐based analysis, we show that impairing mitochondrial fatty acid β‐oxidation (FAO) can induce apoptosis in quiescence‐induced cells and hamper their return to proliferation. Our findings suggest that in addition to mediating energy and redox balance, FAO can play a role in preventing the buildup of toxic intermediates during transitioning to quiescence. Uncovering metabolic strategies to enter, maintain, and exit quiescence can reveal fundamental principles in cell plasticity and new potential therapeutic targets beyond cancer.

## Graphical abstract

Analyses of the metabolic adaptation of human cell lines transitioning into quiescence reveal a metabolic signature common to different cell types and quiescence‐inducing stimuli, indicating fatty acid oxidation as a key vulnerability in quiescent‐induced cancer cells.

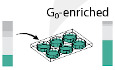

## Introduction

Cellular quiescence (G_0_) is a fundamental operating principle in cells to enable long‐term survival in a reversible nondividing state following cell cycle arrest in G_1_ phase (Hua & Thompson, 2001; Coller *et al*, 2006; Liu *et al*, 2007; Valcourt *et al*, 2012; Cheung & Rando, 2013). Essential functions in the human body, such as the immune response (Hua & Thompson, 2001; Chapman *et al*, 2020), hematopoiesis (Pietras *et al*, 2011), tissue repair, and regeneration (Li & Clevers, 2010; Cheung & Rando, 2013; Cho *et al*, 2019) rely on the ability of cells to enter a quiescent, nondividing state, and subsequently switch back to proliferation. Paradoxically, nonproliferating cells can be found also among tumor cells (Jackson, 1989; Aguirre‐Ghiso, 2007; Giancotti, 2013; Zeuner, 2015). While rare, quiescent cancer cells exhibit increased radio‐ and chemoresistance (Dembinski & Krauss, 2009; Chen *et al*, 2012; Brown *et al*, 2017), and when reactivated from their dormant state can be responsible for cancer recurrence and metastasis (Mellor *et al*, 2005; Chen *et al*, 2012; Sosa *et al*, 2014; Ramirez *et al*, 2016; Brown *et al*, 2017), posing a major challenge in cancer therapy.

Environmental signals indicating unfavorable conditions, including nutrient limitation (Zhang *et al*, 2015), stress (Yang *et al*, 2020), cell–cell contact, or the absence of growth factors (Coller *et al*, 2006; Mitra *et al*, 2018), can trigger the entry into quiescence. A key feature of quiescent cells is the ability to maintain cellular homeostasis in a nondividing state for long periods (i.e., years). Despite their apparent dormancy, quiescent cells remain viable and have the ability to rapidly switch back to fast proliferation. It is hence not surprising that metabolism in quiescence is more active than previously thought. However, while rewiring of metabolism has become a hallmark of rapidly proliferating cancer cells (Vander Heiden *et al*, 2009; Hanahan & Weinberg, 2011; DeBerardinis & Chandel, 2016; Keibler *et al*, 2016; Pavlova & Thompson, 2016; Vander Heiden & DeBerardinis, 2017; Fendt *et al*, 2020), a similar description of the metabolic characteristics in quiescent cancer cells is lagging behind.

Mounting evidence suggests an involvement of metabolism in mediating cellular quiescence across multiple cell types. Quiescent fibroblasts, endothelial cells, adult stem cells, and B‐lymphocytes reportedly exhibit active energy generation and homeostasis‐related metabolic processes (Lemons *et al*, 2010; Coloff *et al*, 2016; Lee *et al*, 2017; Kalucka *et al*, 2018) like fatty acid degradation (Ito *et al*, 2012; Knobloch *et al*, 2017; Kalucka *et al*, 2018), TCA cycle, and oxidative phosphorylation. However, most studies relied on genomic or proteomic measurements, and systematic and direct characterization of the metabolic features of quiescence is missing. Moreover, to what extent does metabolism play a role in the adaptation of nondividing cancer cells to microenvironmental challenges (Hu *et al*, 2011; Carmona‐Fontaine *et al*, 2017; Lyssiotis & Kimmelman, 2017; Kumar *et al*, 2019) or treatment‐induced stress and drug tolerance (Dembinski & Krauss, 2009; Chen *et al*, 2012; Ramirez *et al*, 2016; Brown *et al*, 2017; Yang *et al*, 2020) have remained key open questions.

Here, we monitored metabolic changes in six largely different cell types, including cancer cells, transitioning from rapid proliferation into a quiescent state induced by three different environmental stimuli. To overcome technical limitations in preserving and profiling the metabolic state of quiescent cells, we developed an original methodology that allows differentiating metabolic signatures of cell subpopulations without the need for physical separation (i.e., cell sorting). This approach allowed us to generate an unprecedented compendium of metabolic profiles of quiescent cells in six different cell lines and three quiescence‐inducing conditions. Remarkably, we found direct evidence for a common metabolic adaptation to quiescence that is shared among different cell types and is largely independent of the quiescence stimulus. Consistent with previous findings (Ito *et al*, 2012; Knobloch *et al*, 2017; Kalucka *et al*, 2018), our metabolome‐based analysis revealed a key difference in fatty acid degradation in quiescence. We show that fatty acid β‐oxidation (FAO) becomes a vulnerability in quiescence‐induced cells and suggest that beyond its roles in mediating energy and redox balance, FAO can also prevent the accumulation of toxic intermediates in cells transitioning to quiescence.

## Results

### Quiescent cells rapidly exit growth arrest and resume proliferation

**Figure 1:**
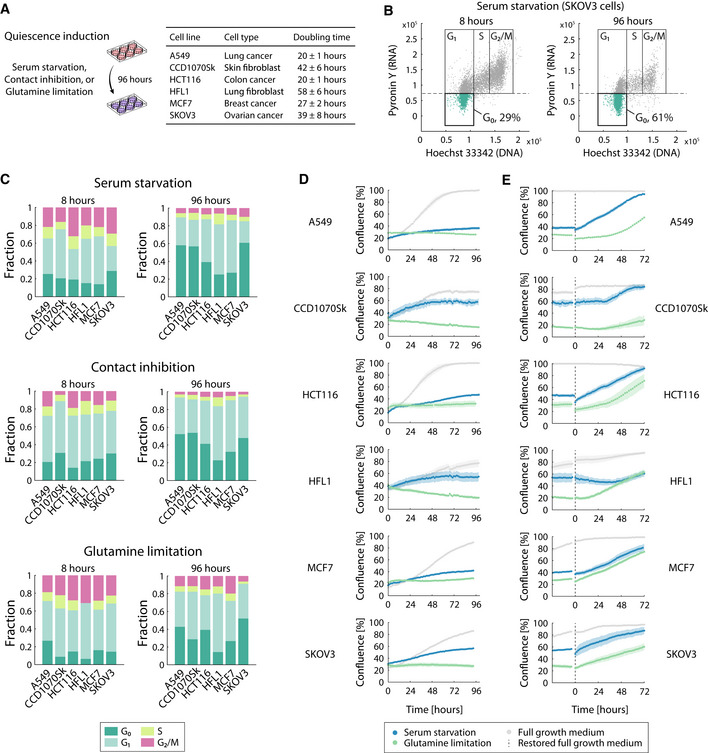
A collection of *in vitro* models for cellular quiescence in diverse human cell types ASchematic illustration of the experimental setup for the induction of quiescence in six different adherently growing cell types using three quiescence stimuli (i.e., serum starvation, contact inhibition, or glutamine limitation) for 96 h. Cell‐type and basal growth characteristics of the six cell lines are reported in the table.BFlow cytometry‐based stratification of cells in different cell cycle phases and in G_0_ (quiescence, 2n DNA, and low RNA content), shown for SKOV3 cells in serum starvation conditions at 8 and 96 h, respectively. Cells were detached, fixed with ethanol, and stained with Hoechst 33342 and Pyronin Y prior to flow cytometry analysis.CDistribution of cell cycle phases in six cell lines exposed to quiescence‐inducing conditions (see Panel A) for 8 and 96 h, respectively. Each stacked bar plot reports the fraction of cells in G_0_, G_1_, S, and G_2_/M phases, estimated using the assay depicted in panel B.DCell confluence of adherent cell cultures in quiescence‐inducing conditions (i.e., serum starvation and glutamine limitation) was monitored using time‐lapse microscopy in a plate reader (TECAN Spark 10M) in 1.5‐h intervals. Contact‐inhibited cells no longer grow in monolayers and could not be accurately imaged with this approach. Data points and shaded areas indicate mean ± standard deviation (SD) over three biological replicates. Approximately 9,500, 4,500, 19,500, 6,700, 19,500, and 9,500 cells/well were seeded for glutamine limitation and serum starvation of A549, CCD1070Sk, HCT116, HFL1, MCF7, and SKOV3 cells, respectively.EDynamic changes in cell confluence (as described in panel D) after restoring normal growth conditions (at 0 h, i.e., fresh growth media containing both fetal bovine serum and glutamine) after 96 h of quiescence induction. Data points and shaded areas indicate (mean ± SD) over three biological replicates.

A reversible cell cycle exit in G_1_ phase and the ability to resume proliferation are defining characteristics of quiescent (G_0_) cells (Jackson, 1989; Coller *et al*, 2006; Coller, 2011; Zeuner, 2015; Cho *et al*, 2019). However, whether these abilities depend on the cell type or specific quiescence‐inducing stimuli remains unclear. Here, we monitored the transition between rapid proliferation and quiescence in a diverse set of adherently growing human cell lines and under quiescence‐inducing conditions (Fig 1A). We chose six human cell lines exhibiting different proliferation rates, i.e., doubling times between 20 and 58 h (Fig 1A). Specifically, we included four cancer cell lines of different tissue origins (i.e., A549 lung, HCT116 colon, MCF7 breast, and SKOV3 ovarian cancer cells, Fig 1A) with largely different genetic backgrounds (i.e., mutations (Ikediobi *et al*, 2006)) and basal metabolic states (Ortmayr *et al*, 2019) (Appendix Fig S1), and two nontransformed fibroblast cell lines (i.e., CCD1070Sk (Liu *et al*, 2007) and HFL1 (Coller *et al*, 2006)) previously used to model cellular quiescence *in vitro* (Coller *et al*, 2006; Liu *et al*, 2007). As environmental stimuli inducing a transition from proliferation into a quiescent state, we selected two that are well‐established: serum starvation (Coller *et al*, 2006; Liu *et al*, 2007; Mitra *et al*, 2018) and contact inhibition (Coller *et al*, 2006; Leontieva *et al*, 2014; Mitra *et al*, 2018), and a less conventional stimulus that is more directly linked to cellular metabolism, i.e., glutamine limitation. All three conditions also mimic *in vivo* shifts in the cancer environment (Carmona‐Fontaine *et al*, 2017; Lyssiotis & Kimmelman, 2017), i.e., the lack of growth signals in a foreign niche, space limitations in an intact tissue or solid tumor, or nutrient gradients in tissue regions secluded from blood vessels, respectively. To induce quiescence, we exposed the cell lines separately to each of the three quiescence stimuli for 96 h. Cells were seeded and maintained at confluence (contact inhibition), or subconfluent cells were incubated with media lacking fetal bovine serum (serum starvation) or glutamine (glutamine limitation), respectively. We quantified the end‐point fraction of quiescent (G_0_) cells in each condition using a previously established flow cytometry‐based assay (Kim & Sederstrom, 2001) (Fig 1B, Dataset EV1).

At 96 h after exposure to quiescence stimuli, cell populations consisted for the vast majority (~90%) of cells in either G_1_ or G_0_ phase (2n DNA content) (Fig 1B and C, and Appendix Fig S1, Dataset EV1). The fraction of G_0_ phase cells in the population increased in all tested conditions, on average 2.25‐fold, reaching between 30 and 60% G_0_ cells (Fig 1C). Next, we asked whether the dynamics of the transition between rapid proliferation and quiescence is affected by the diversity in cell types and physiology (i.e., basal growth rates), as well as quiescence‐inducing conditions. After inducing quiescence by either serum starvation or glutamine limitation, we observed a rapid reduction in growth rates (Fig 1D and Appendix Fig S1, Dataset EV2), and a near‐complete growth arrest after approximately 72 h. To probe the ability to spontaneously resume proliferation, after 96 h of quiescence induction we restored normal growth conditions (i.e., full growth media containing both glutamine and serum). Remarkably, we observed an almost instantaneous exit from growth arrest and a return to a rapid proliferation in all cancer cell models (Fig 1E, Dataset EV2), with the two fibroblast cell lines exhibiting a slight delay and resuming growth within 24 h. Hence, our *in vitro* model allows reliably probing key functional characteristics of quiescent cells, and the collected growth phenotypic data uncovered largely similar growth dynamics upon entry and exit from quiescence. This similarity hinted at adaptive processes that are largely independent of cell types and quiescence‐inducing stimuli and potentially, not only mediate the switch from proliferation to quiescence but can also prepare for regrowth (Fig 1E and Appendix Fig S1).

### Cells in G_0_ phase display a common metabolic signature

The ability of cells to rapidly switch between proliferation and quiescence requires highly coordinated metabolic adaptation to maintain homeostasis and avoid exhaustion of essential intermediates and/or the buildup of toxic intermediates, and at the same time prepare to rapidly resume growth by providing the necessary energy and biosynthetic precursors for cell division (Vander Heiden *et al*, 2009; Boroughs & DeBerardinis, 2015; Keibler *et al*, 2016; Pavlova & Thompson, 2016). However, despite mounting evidence for quiescence‐specific transcriptional programs (Coller *et al*, 2006; Liu *et al*, 2007), direct and systematic evidence of universal metabolic characteristics mediating adaptation of cells in G_0_ phase is missing.

Measuring metabolic differences between quiescent and proliferating cell subpopulations remains a major challenge. Like in an *in vivo* context, quiescence‐induced cell cultures *in vitro* harbor mixed populations with co‐occurring cells in G_0_ and other cell cycle phases (Fig 1, Dataset EV1). While cell sorting of heterogeneous populations allows for selection of cells in different cell cycle phases (e.g., only G_0_ cells), this technique implies lengthy and invasive steps, which, as previously shown (Llufrio *et al*, 2018; Binek *et al*, 2019), can profoundly alter the metabolic state of cells. We found that the necessary initial step of enzymatic cell detachment commonly used to generate single‐cell suspensions for cell sorting, induced metabolic changes that can mask the differences between cell lines from different tissues of origin (Appendix Fig S2, Dataset EV3). In addition, we noted increased technical variability in ion intensity measurements of detached‐cell extracts (Appendix Fig S2), presumably arising from the multiple sample processing steps (e.g., centrifugation and washing). Thereby, while suitable for genome or proteome profiling (Coller *et al*, 2006; Ly *et al*, 2017; Hoogendijk *et al*, 2019; Herr *et al*, 2020) where the consequences of perturbations manifest within minutes to hours (Sabatier *et al*, 2018), classical cell sorting approaches hamper a systematic and unbiased metabolic characterization of quiescent cell subpopulations. To overcome this limitation and enable direct metabolome profiling in quiescent cells, we sought to develop a novel approach for the sorting‐free separation of metabolic signatures in distinct cell subpopulations.

**Figure 2:**
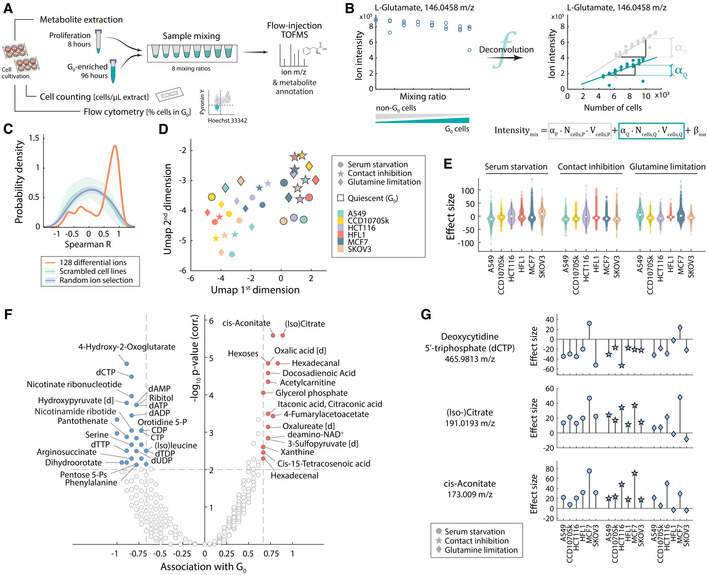
Metabolic profiling of quiescent (G_0_) cell subpopulations using sorting‐free *in silico* deconvolution A, BSchematic representation of the multistep approach for the *in silico* separation of subpopulation metabolic signatures without physical separation. The mixing of cell extracts obtained from cultures at 8 h (mostly G_1_, S, and G_2_/M phase cells) and 96 h (quiescence‐enriched, mainly cells in G_0_ and G_1_, see Fig 1C) allows for controlling the number of G_0_ and non‐G_0_ cells in each sample prior to FIA‐TOFMS analysis (Fuhrer *et al*, 2011). Panel B depicts the linear regression model used to determine separate estimates α_Q_ and α_P_ describing the relative intracellular abundance of individual metabolites in quiescent and proliferating cells, respectively.CComparison (Spearman correlation) of reconstructed metabolome profiles of five cell lines after mixing and deconvolution (Appendix Fig S4) to known metabolic differences between the five cell lines (Ortmayr *et al*, 2019). The plot shows the probability density (kernel smoothing function) of Spearman correlation coefficients for 128 ions with characteristic variance across cell lines (orange curve). Distributions expected at random, i.e., correlations obtained after scrambling cell line labels, or randomly selecting putatively annotated metabolites are shown in green and blue, respectively. Shaded errors represent the standard deviation across 100,000 permutations.DDimensionality reduction (Uniform Manifold Approximation and Projection, UMAP (preprint: McInnes *et al*, 2018)) of deconvoluted metabolic profiles of quiescent (G_0_) vs. non‐G_0_ cells (panel A and B), using Z‐scored relative abundances of 1940 putatively annotated metabolites in 6 cell lines × 3 stimuli × 2 cell states and Spearman similarity metric.EDistribution of metabolic differences (effect size) between G_0_ and non‐G_0_ cells in six cell lines exposed to three different quiescence‐inducing stimuli, depicted as a violin plot. The violin shape is given by the Kernel density, overlaid data points represent individual metabolites (in total 1940 putatively annotated metabolites), and white circles indicate the median.FVolcano plot of common metabolic changes in G_0_ cells. X‐axis values indicate the association with G_0_, i.e., the fraction of G_0_ models in which a metabolite is found at higher (positive association) or lower (negative value) abundance in G_0_ cells across the 18 cell models. The statistical significance (y‐axis) of the quiescence association was estimated for each metabolite using a hypergeometric test and an iterative testing scheme (see Materials and Methods section and Appendix Fig S4). Highlighted metabolites change consistently in two‐thirds or more conditions (G_0_ association >= 0.667, *P*‐value < 0.01, Benjamini–Hochberg correction). Only ions annotated to metabolites listed in the KEGG database are shown in the plot. The suffix [d] indicates α‐keto acids detected as phenyl hydrazine derivatives (Zimmermann *et al*, 2014).GEffect size profiles for selected putatively annotated metabolites showing significantly quiescence‐associated patterns across six cell lines and three quiescence stimuli (see panel F).

In a metabolite extract from a homogeneous cell population, the total abundance of any given metabolite is linearly dependent on the number of cells extracted and can be measured and compared between cell types using mass spectrometry techniques (Ortmayr *et al*, 2019). Similarly, we can assume that in a mixed cell population, the total measured metabolite abundance is a linear combination of the characteristic metabolite abundances in each individual cell subpopulation, provided that the presence or relative abundance of each distinct population in the culture does not affect the metabolism or general cell biology of the other in a way that might change the amount of any given metabolite per cell. Here, building on this general principle, we conceived an experimental‐computational framework based on a series of cell extract samples containing different relative amounts of two subpopulations of interest, and a linear regression model to separate *in silico* the characteristic metabolic profiles of the individual subpopulations. In brief, the method consists of three steps (Fig 2A and B). First, metabolites are extracted separately from two cell cultures enriched for either of the two subpopulations. In the second step, the two cell extract samples are mixed at several defined ratios and analyzed by high‐throughput nontargeted metabolomics (FIA‐TOFMS (Fuhrer *et al*, 2011)). In the third and final step, we solve a multilinear regression model to obtain coefficients representative of the relative metabolite abundance in the individual cell subpopulations (Fig 2B).

The approach relies on two key assumptions, i.e., that the key metabolic characteristics of each subpopulation are not affected by the relative proportion of subpopulations, and that metabolite concentrations lie within the range where measured ion intensities linearly increase with the number of cells (i.e., extracted biomass). It is worth noting that when either of the two assumptions is not fulfilled, we expect the relationship between metabolite concentration and ion intensity to significantly deviate from linearity. We verified that the linearity of MS measurements (*R* ^2^ > 0.9) was preserved across multiple cell line extracts (i.e., different sample matrices, Appendix Fig S3, Dataset EV3) by spiking metabolites into extracts of three cell lines exhibiting different basal levels of high‐abundant metabolites like glutamate, glutathione, and 2‐oxoglutarate (Appendix Fig S3 and Materials and Methods, Dataset EV3). For all metabolites, the linear range extended two orders of magnitude beyond metabolite concentrations in a typical cell extract (Appendix Fig S3). Deviations from linearity were observed for only 18% of metabolites on average (Appendix Fig S3), supporting the validity of our assumptions. Ions not following a linear dependency between cell number and ion intensity are filtered out during data processing.

To benchmark the methodology for the separation (deconvolution) of subpopulation metabolic profiles, we mimicked co‐occurring signals by mixing metabolite extracts from different cell lines and applied the deconvolution approach to reconstruct the individual cell line metabolic profiles (Appendix Fig S4). To that end, we generated cell extracts from five cell lines grown individually, prepared mixes in all possible pairwise combinations of cell lines, and applied the workflow described above to reconstruct the individual cell line profiles (Dataset EV3). After deconvolution, we compared (Spearman correlation) the relative metabolite abundances derived from mixed metabolite extracts against previously measured characteristic metabolic differences between the five cell lines (Ortmayr *et al*, 2019) (Appendix Fig S4, Dataset EV3). For 128 metabolites exhibiting large variation in abundance across cell lines in pure extracts (greater than 3 standard deviations variance across the 5 cell lines), we observed high correlations (median Spearman correlation 0.7) that were significantly higher than for ions exhibiting no characteristic difference across these five cell lines (622 metabolites, *P*‐value 8.9e‐13). Hence, we validated the ability of our approach to resolve distinct metabolic characteristics of different cell types from the profiling of mixed extracts.

We applied the sorting‐free deconvolution method to profile the relative difference in the abundance of 1940 putatively annotated metabolites between quiescent (G_0_) and proliferating (G_1_, S, and G_2_/M phases) cell subpopulations, in the six cell types and three quiescence stimuli (Fig 1, Dataset EV4). To that end, we generated cell extracts at 8 and 96 h after applying quiescence‐inducing conditions, enriched for non‐G_0_ and G_0_ cells, respectively (Fig 1C). Deconvolution using the mixing scheme revealed a distinct signature of G_0_‐cell populations (Appendix Fig S4) that was not discernible by simply comparing the metabolic profiles at 8 and 96 h after quiescence induction (i.e., without deconvolution, Appendix Fig S4, Dataset EV4). This further reinforces that conventional approaches used for the metabolome profiling of quiescent cells, where the bulk population is measured, may overlook characteristic differences in quiescent cell subpopulations.

Interestingly, we found that the differences in metabolite abundances were mostly due to the cell state (i.e., G_0_ vs. non‐G_0_) rather than cell type or quiescence stimulus (Fig 2D), while only 31 ions showed significant variation across stimuli (ANOVA *P*‐value <0.05, Benjamini–Hochberg correction, Appendix Fig S5, Dataset EV4). A common metabolic signature of cells in G_0_ that is largely invariant to cell type or quiescence‐inducing stimuli suggests a universal metabolic program underlying the proliferation‐quiescence transition, consistent with our observation of largely similar growth dynamics (Fig 1, Dataset EV1).

To systematically identify common features of the metabolic adaptation to quiescence, for each metabolite we estimated how frequently cells exhibit higher or lower levels in G_0_ (effect size, Fig 2E) across the 18 conditions (Fig 2F, Appendix Fig S4, Dataset EV4, Materials and Methods section). We identified 164 putatively annotated metabolites exhibiting an association with G_0_, i.e., that showed consistently increased or decreased abundances in G_0_ cells, irrespective of cell type or quiescence stimulus (Fig 2F, abs. G_0_ association > = 2/3, Benjamini–Hochberg corrected *P*‐value <0.01, hypergeometric statistical test).

While consistently reduced pool sizes of several nucleotides and intermediates of nucleotide biosynthesis (precursors dihydroorotate and pentose 5‐phosphates) are likely the result of the arrest of DNA replication, quiescence‐associated metabolites reflected adaptive changes in metabolism that likely go beyond an indirect effect of growth arrest (Fig 2F). For example, we found metabolites that exhibited consistently higher levels in G_0_ cells, such as cis‐aconitate and (iso‐)citrate (in 15 and 16 out of 18 quiescence models, respectively, Fig 2G). On average, citrate and aconitate accumulations were slightly lower in glutamine limitation as compared to serum starvation and contact inhibition (Fig 2G), potentially reflecting the indirect effect of an interrupted supply of a major anaplerotic TCA cycle substrate like glutamine (DeBerardinis *et al*, 2007; DeBerardinis & Cheng, 2010; Daye & Wellen, 2012). Maintaining large metabolite pools can report on active strategies supporting cell survival in the quiescent state, and possibly prepare quiescent cells for resuming growth (e.g., xanthine (Link *et al*, 2015)). Together with citrate and aconitate, we noted a significantly quiescence‐associated accumulation (corrected *P*‐value <0.01) of several metabolites located in lipid metabolism (acetylcarnitine, glycerol 3‐phosphate, and hexadecanal, Fig 2F). These changes suggest for differential regulation of lipid metabolism in quiescent cells. Interestingly, fatty acid β‐oxidation has been associated with quiescence in cell models other than cancer, i.e., in endothelial (Kalucka *et al*, 2018) and stem cells (Ito *et al*, 2012; Knobloch *et al*, 2017; Shyh‐Chang & Ng, 2017). However, its fundamental functional role, and whether fatty acid degradation also plays a role in cancer cell quiescence remains to be elucidated. Here, we observed increased levels of acetylcarnitine that could indicate an engagement of the carnitine system (Melone *et al*, 2018) responsible for fatty acid transport across the mitochondrial membrane, consistent with a possible role of fatty acid β‐oxidation and its product acetyl‐CoA in the accumulation of aforementioned TCA intermediates (e.g., citrate).

Altogether, our data uncover common metabolome characteristics of quiescent cells across several quiescence‐inducing conditions and multiple cell types, including cancer cells. While previous studies have focused on cellular quiescence in a physiological (i.e., noncancer) setting, here we show global metabolic rearrangements that hint at the involvement of fatty acid degradation also in cancer cells reversibly transitioning between proliferation and quiescence.

### With quiescence entry, FAO becomes a metabolic vulnerability

**Figure 3:**
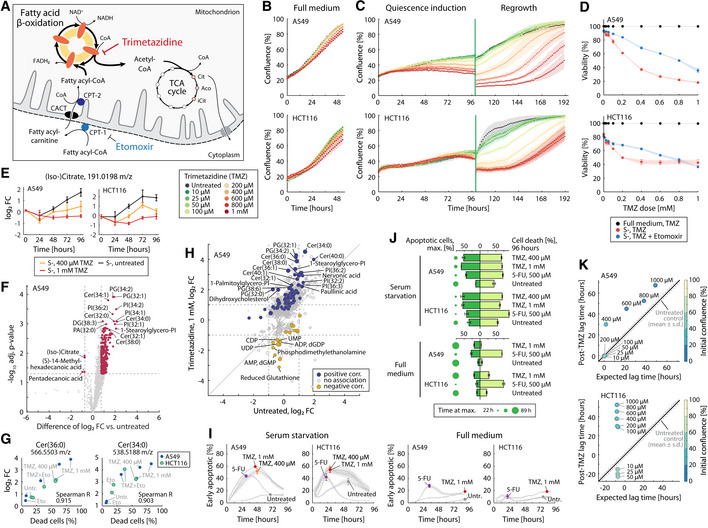
FAO inhibitor trimetazidine (TMZ) impairs viability and regrowth of quiescent cancer cells ASchematic representation of mitochondrial fatty acid β‐oxidation. In the cytosol, long‐chain fatty acids are conjugated to carnitine by carnitine palmitoyltransferase 1 (CPT1, irreversibly inhibited by the CoA conjugate of etomoxir) for transport across the mitochondrial membrane by acylcarnitine translocase (CACT). In the mitochondrial matrix, the fatty acyl chain is released from carnitine and conjugated to coenzyme A by CPT2. Fatty acyl‐CoAs are shortened stepwise by β‐oxidation enzymes acyl‐CoA dehydrogenase, enoyl‐CoA hydratase, and 3‐ketoacyl‐CoA thiolase, generating FADH_2_, NADH, and one molecule of acetyl‐CoA per reaction cycle. Trimetazidine competitively inhibits the last step of FAO, the cleavage of acetyl‐CoA by 3‐keto acyl‐CoA thiolase.BGrowth phenotypic changes induced by TMZ treatment in A549 and HCT116 cells grown in full growth medium (RPMI‐1640 with 5% dialyzed FBS). Cell confluence as a proxy of cell numbers was monitored using automated time‐lapse microscopy in a plate reader (TECAN Spark 10M). Data points and shaded areas indicate mean ± SD over three biological replicates.CGrowth phenotypic changes induced by TMZ treatment in quiescence‐inducing conditions (serum starvation) in A549 and HCT116 cancer cells. Growth was monitored via cell confluence as described in panel B. At 96 h, cells were stimulated to exit quiescence by replacing serum‐free media with a fresh growth medium without TMZ (regrowth). Data points and shaded areas indicate mean ± SD over three biological replicates.DViability of quiescence‐induced cells treated with TMZ, or with TMZ plus 10 μM etomoxir was assessed using a fluorescence imaging assay involving two fluorescent dyes: Hoechst 33342 (all nuclei) and propidium iodide (dead cells). Data points and error bars indicate mean ± SD over three biological replicates.EDynamic changes in the intracellular levels of citrate in A549 and HCT116 cells induced to enter quiescence by serum starvation with or without TMZ. Fold changes were estimated relative to steady‐state levels in full medium (Dubuis *et al*, 2018). Data points and error bars indicate mean ± SD over three biological replicates.FVolcano plot showing TMZ‐induced metabolic changes (1 mM, 48 h) in A549 cells in serum starvation (see also Appendix Fig S8). Shown are differences in log_2_ fold change between TMZ treatment and serum starvation alone (x‐axis), and their statistical significance (y‐axis, adjusted *P*‐value, *t*‐test, and Benjamini–Hochberg correction).GCorrelation of ceramide levels (log_2_ fold change, y‐axis) with the loss of viability under TMZ treatment (fraction of dead cells at 96 h, x‐axis, see panel D).HComparison of relative changes (log_2_ fold‐change) in the abundance of 1791 putatively annotated metabolites upon TMZ treatment (1 mM, y‐axis) against untreated quiescence‐induced A549 cells (x‐axis). Fold‐changes are calculated relative to the expected ion intensity in full medium at a steady state (Dubuis *et al*, 2018). Significant positive and negative correlations (Spearman, *P* < 0.01) with cell death at 96 h are highlighted in blue and yellow, respectively.IFractions of early apoptotic cells in A549 or HCT116 cancer cell cultures in quiescence‐inducing conditions (serum starvation, left panels) or full growth medium (5% dFBS, right panels), treated with TMZ (0.4 or 1 mM), 5‐fluorouracil (5‐FU, 0.5 mM, positive control), or medium (untreated). Early apoptotic cells were detected using ApoTracker Green under co‐staining with Hoechst 33342 (nuclei) and propidium iodide (dead cells). Cell staining was continuously monitored in 3‐h intervals for 96 h using automated fluorescence microscopy in a TECAN Spark Cyto. The full time course is shown in Appendix Fig S8. Data points and shaded areas indicate mean ± SD over three biological replicates. Approximately 3,400 and 7,500 cells/well were seeded of A549 and HCT116 cells, respectively.JSummarized differences in the dynamics of apoptosis induction by TMZ treatment between quiescence‐inducing conditions (upper panel) and full growth medium (lower panel). In each treatment, the maximum fraction of apoptotic cells and the time at which the maximum was reached (dark green bars) is reported alongside the fraction of dead cells at 96 h (light green bars, full time course in Appendix Fig S8). Apoptosis induction was assessed as described in panel I. Bar length and error bars indicate mean ± SD over three biological replicates.KComparison of lag times (i.e., time to resume cell duplication) after serum starvation with or without TMZ treatment in A549 and HCT116 cancer cells. Lag times were estimated from cell confluence data shown in panel C, by first estimating the maximum growth rate (see Materials and Methods section for a detailed description), and subsequently finding the intersection between the tangent to the point with the highest growth rate and the initial confluence (i.e., after media change at 96 h). To correct for the intrinsic dependency of lag time on the initial confluence (Appendix Fig S9), expected lag times (x‐axis) were determined by linear interpolation of lag times measured in untreated cells at different initial confluences (Appendix Fig S9).

Fatty acid β‐oxidation (FAO) is a major pathway in the degradation of fatty acids (as fatty acyl‐CoAs) into acetyl‐CoA units (Fig 3A) (Houten *et al*, 2016). Acetyl‐CoA can further be converted to citrate, forming a key intermediate not only in energy generation (TCA cycle) but also in the regeneration of redox equivalents (NADPH, isocitrate dehydrogenase reaction) in mitochondria or the cytosol. Thus, FAO can ensure cellular energy and redox homeostasis, and also provides citrate for lipogenesis, protein, or histone acetylation (Melone *et al*, 2018). While FAO has been associated with different functions in cellular quiescence in noncancer cells (Knobloch *et al*, 2017; Shyh‐Chang & Ng, 2017; Kalucka *et al*, 2018), its function in the adaptation of cancer cells to quiescence remains to be clarified.

The common metabolic characteristics of G_0_ cells (Fig 2F) hint at a differential regulation of fatty acid degradation, reflected in the accumulation of citrate and cis‐aconitate, which exhibit the most significant association with G_0_ (Fig 2F and G, Benjamini–Hochberg corrected *P*‐value 4.87e‐9 and 6.65e‐9, respectively). To test whether this quiescence‐associated accumulation of FAO‐related metabolites in G_0_ cells reports an increased activity of fatty acid β‐oxidation in quiescence, we used trimetazidine, a competitive inhibitor of 3‐ketoacyl‐CoA thiolase (Lopaschuk *et al*, 2003) catalyzing the third and last enzymatic step of FAO (Fig 3A). Cells in full growth media were insensitive to trimetazidine across a wide range of concentrations (Fig 3B, Dataset EV5), indicating that FAO is dispensable during rapid cell proliferation. By contrast, in A549 and HCT116 cancer cells under serum starvation conditions, trimetazidine treatment caused drastic dynamic changes in cell confluence (Fig 3C, Dataset EV5) and induced cell death in a dose‐dependent manner (Fig 3D, Dataset EV5). Similarly, trimetazidine impaired viability also when applied 96 h after quiescence induction, and also in cultures enriched for G_0_ cells by glutamine limitation (Appendix Fig S6, Dataset EV5). Using dynamic metabolome profiling (Dubuis *et al*, 2018), we found that trimetazidine treatment abolished the time‐dependent accumulation of citrate during G_0_ induction by serum deprivation (Fig 3E, Dataset EV6). Targeted LC–MS/MS measurements confirmed this observation (Appendix Fig S7, Dataset EV6), providing supportive evidence that the G_0_‐associated citrate accumulation reflects an increased activity of fatty acid β‐oxidation in G_0_ cells. Moreover, in line with our observation that fatty acid β‐oxidation is dispensable during proliferation (Fig 3B), trimetazidine did not alter citrate levels in cells in full medium containing 5% dFBS and 2 mM glutamine (Appendix Fig S7). Collectively, these results indicate that fatty acid degradation plays a central role in cells transitioning from rapid proliferation to G_0_ phase.

Previous studies exploring the function of FAO in quiescence models outside of cancer identified a link to energy production in adult stem cells (Ito *et al*, 2012; Knobloch *et al*, 2017) or redox homeostasis in endothelial cells (Kalucka *et al*, 2018). Downstream metabolism of FAO‐derived citrate can fuel oxidative phosphorylation and ATP synthesis, as well as the NADP^+^‐dependent isocitrate dehydrogenase reaction to regenerate NADPH. To test whether FAO is required to supply citrate for these functions, we supplemented serum‐starved and trimetazidine‐treated cells with citrate or reduced glutathione (GSH), a key endogenous antioxidant. Remarkably, the strong toxicity induced by trimetazidine was rescued neither by citrate nor by GSH (Appendix Fig S7, Dataset EV5), indicating that in our quiescence models, the role of FAO likely goes beyond the previously suggested functions to maintain redox homeostasis or use of FAO‐derived acetyl‐CoA/citrate as an energy source.

We hypothesized that if the inhibition of FAO affects mitochondrial metabolic intermediates, the toxicity of trimetazidine for quiescence‐induced cells is linked to accumulating FAO substrates in mitochondria, which can impair mitochondrial function and trigger apoptosis (Ostrander *et al*, 2001; Penzo *et al*, 2002; Artwohl *et al*, 2009). To test this possibility, we investigated whether a reduced transport of FAO substrates into mitochondria can alleviate trimetazidine‐induced toxicity. To this end, we used etomoxir, an irreversible inhibitor of carnitine palmitoyltransferase CPT1A, the limiting component of the carnitine system for the import of longer‐chain fatty acids into mitochondria (Melone *et al*, 2018) (Fig 3A). Co‐treatment with etomoxir reduced the toxic effect of trimetazidine in quiescence‐induced cells (Fig 3D and Appendix Fig S7, Dataset EV5), indicating that trimetazidine‐induced toxicity is at least partially caused by increased levels of potentially toxic intermediates in mitochondria. Nevertheless, etomoxir is not able to completely abolish trimetazidine toxicity (Fig 3D). The residual toxicity is consistent with a remaining citrate accumulation (Appendix Fig S7, Dataset EV6) and is likely due to incomplete inhibition of fatty acid‐driven respiration by etomoxir (Divakaruni *et al*, 2018), or a compensatory role of peroxisomal FAO (Violante *et al*, 2013, 2019).

To shed light on the mechanism underlying trimetazidine‐induced toxicity, we measured metabolic changes in quiescence‐induced cells treated with trimetazidine at 24, 48, 72, and 96 h (Fig 3F and Appendix Fig S8, Dataset EV6). Already at 24 and 48 h, we detected significantly higher levels (adjusted *P*‐value <0.05, Benjamini–Hochberg correction) of several metabolites related to lipid metabolism and phospholipids in trimetazidine‐treated as compared to untreated quiescence‐induced cells (Fig 3F and Appendix Fig S8, Dataset EV6). Among these early metabolic responses to trimetazidine, we found accumulations of several bioactive lipid species with signaling functions, such as lysophospholipids (stearoyl and arachidonoyl phosphoinositol, Fig 3F and Appendix Fig S8) and ceramides containing long‐chain fatty acids (e.g., sum compositions 32:0, 32:1, 34:0, 34:1, 38:0). Elevated levels of certain ceramides have been associated with the progression of apoptotic cell death (Rudd & Devaraj, 2018) by promoting the permeabilization of the mitochondrial membrane (Chang *et al*, 2015; Ogretmen, 2018; Dadsena *et al*, 2019). Hence, we hypothesized that impaired FAO can lead to the generation of cytotoxic intermediates such as ceramides, thus potentially exposing quiescence‐induced cells to apoptotic cell death.

To test this hypothesis, we systematically searched for metabolic changes associated with trimetazidine‐induced toxicity. To that end, we estimated the correlation (Spearman) between the reduction in viability at 96 h (Fig 3D) and characteristic relative changes in metabolite abundances in G_0_‐induced A549 and HCT116 cells treated with trimetazidine (400 μM or 1 mM), alone or in combination with 10 μM etomoxir (Dataset EV6). We found that the metabolic signature associated with cell death consists of several ceramide species (Fig 3G and H), reinforcing a potential link between FAO inhibition by trimetazidine and the accumulation of toxic metabolites such as apoptosis‐inducing ceramides. To verify apoptosis as the cell death mechanism triggered by trimetazidine, we monitored the induction of apoptosis by detecting externalized phosphatidylserine (PS) residues with ApoTracker Green (BioLegend, (Barth *et al*, 2020)) in a fluorescence imaging assay (Fig 3I and Appendix Fig S8, Dataset EV6). Consistent with the accumulation of ceramides (Fig 3G), we observed a rapid significant (*P*‐value = 6.8e‐08) increase in the fraction of early apoptotic cells (i.e., displaying PS) in trimetazidine‐treated quiescence‐induced cells as compared to untreated serum‐starved cells (Fig 3I and J), reaching up to 60% of all cells within the first 48 h in both A549 and HCT116 cells (Fig 3I and Appendix Fig S8). Subsequently, we observed a large increase in the number of cells with compromised plasma membranes (additional staining by live cell‐impermeable propidium iodide, Fig 3J and Appendix Fig S8, Dataset EV6), indicating late stages of apoptosis and cell death in agreement with the previous viability analysis (Fig 3D). Interestingly, co‐treatment with etomoxir did not abolish the increase in early apoptotic cells but reduced cell death at 96 h (Fig 3D and Appendix Fig S8). The lower rate of apoptotic cell death reinforces the hypothesis that trimetazidine‐induced toxicity is at least in part caused by the action of toxic intermediates in mitochondria. Hence, a co‐treatment with etomoxir, by reducing the import of fatty acids into mitochondria, can slow down the buildup of toxic metabolites. Altogether, these results suggest that maintaining homeostasis by controlling fatty acid degradation is critical to preserve mitochondrial integrity and avoid induction of the apoptotic cascade in cells reversibly transitioning into quiescence.

Several metabolites showing a reduced abundance after trimetazidine treatment are intermediates of nucleotide, energy, and redox metabolism (e.g., ADP, AMP, NADPH, reduced glutathione, 2‐oxoglutarate, Fig 3H and Appendix Fig S8), potentially indicating that FAO is involved in maintaining homeostasis of cofactors and metabolite pools that are not only relevant to survival in quiescence but also to rapidly resume proliferation. Furthermore, citrate, whose abundance was decreased by FAO inhibition (Fig 3E), is a key intermediate not only in energy metabolism but also in lipid biogenesis (Bauer *et al*, 2005). Hence, we hypothesized that, in addition to maintaining homeostasis during quiescence induction, fatty acid degradation also plays a role in preparing cells for reversing quiescence. We tested whether the ability to exit quiescence and switch back to rapid proliferation is affected by prior trimetazidine treatment, by restoring normal growth conditions (i.e., complete growth medium containing 5% dialyzed FBS) after 96 h of trimetazidine treatment in serum starvation. Strikingly, while untreated serum‐starved cells readily escaped the growth arrest, prior treatment with more than 200 μM trimetazidine increased the time necessary to restart cell proliferation (i.e., lag time, Fig 3C and K, Dataset EV5). Although we observed a basal dependency of lag time on the starting cell density (Appendix Fig S9, Dataset EV5), lag times observed after trimetazidine treatment exceeded the expected delay in regrowth simply due to reduced cell confluence caused by trimetazidine toxicity in quiescence‐induced cells (Fig 3K). Similarly, because most dead cells are removed during media change at 96 h (green vertical line in Fig 3C, Appendix Fig S9), we concluded that the increased lag time was not a mere consequence of higher numbers of dead cells in the population after trimetazidine treatment. Supplementation of citrate, whose abundance is decreased by trimetazidine treatment, did not restore rapid regrowth (Appendix Fig S9), indicating that the growth lag is not simply linked to a decreased citrate availability for lipid biosynthesis and/or generation of energy and redox cofactors. Not only the levels of citrate were decreased by trimetazidine but also levels of other intermediates of oxidative energy metabolism (e.g., 2‐oxoglutarate), nucleotides (e.g., AMP, ADP), and redox cofactors (e.g., NADPH, NAD/H), potentially indicating a key role of mitochondrial metabolism and respiration in mediating the rapid switch of nongrowing cells back to proliferation and cell cycle progression (Ahn *et al*, 2017).

Our experimental evidence suggests that while fatty acid degradation is dispensable during rapid proliferation, its proper function and maintenance of mitochondrial integrity becomes crucial for homeostasis during quiescence‐induced growth arrest, and also for preparing cells for rapid growth resumption. Thus, our findings demonstrate that the transition into cellular quiescence can impose metabolic dependencies that are fundamentally different from proliferating cells, and potentially offer unique opportunities to selectively target quiescent cancer cells.

## Discussion

While cancer cells are typically associated with rapidly proliferating cell states, nondividing, quiescent cancer cells pose a serious risk for cancer recurrence and represent a key challenge to conventional anticancer therapies. Moreover, because *in vivo* identification and selective isolation of quiescent cancer cells is complicated, the metabolic characteristics of quiescent cancer cells are largely unexplored. Here, to bypass major technical bottlenecks, we developed an innovative approach to systematically chart fundamental metabolic characteristics that differentiate proliferating from quiescent cells *in vitro*. This new methodology enables delineating metabolic differences between coexisting cell subpopulations without the need for laborious, time‐consuming, and invasive cell sorting procedures. Compared with other *in silico* frameworks able to remove contaminations of cancer tissue samples with nontumor cells or vice versa in metabolome profiling data (Wang *et al*, 2016), our framework is able to directly establish relative metabolite abundances in distinct cell subpopulations, and can in principle be applied to deconvolute more than 2 coexisting subpopulations.

It is noteworthy that our approach does not require samples of pure cell populations. Instead, a relative enrichment of individual subpopulations is sufficient to generate gradients of each subpopulation with the aid of the mixing scheme. Hence, we envisage that our approach could complement single‐cell technologies, like single‐cell transcriptomics (Aldridge & Teichmann, 2020), that allow defining different cell types in complex tissue samples, expanding the deconvolution of metabolic signatures in bulk measurements beyond *in vitro* systems. Here, this method enabled us to systematically characterize differential metabolic profiles in six cell types and three quiescence‐inducing conditions, generating an unprecedented resource to directly investigate the role of metabolism in mediating the transition from rapid proliferation into quiescence.

While more cell lines and conditions need to be tested to further support the generalization of our findings, we discovered characteristic metabolic adaptive changes that are common to diverse quiescence‐inducing environmental conditions and largely different cell types, suggesting a central role of metabolism in the reversible transitioning between proliferation and quiescence (G_0_). Our analysis uncovered quiescence‐associated changes in central metabolic pathways (e.g., from nucleotide and amino acid to energy, redox, and cofactor metabolism) and storage metabolism (e.g., xanthine, hexose sugars) (Fig 2F), suggesting widespread adaptive changes to mediate (Andrade *et al*, 2021) cellular homeostasis in quiescence, and potentially aid rapid regrowth (Link *et al*, 2015). Remarkably, the specific metabolic characteristics of G_0_ cells revealed metabolic functions like fatty acid β‐oxidation that are dispensable during proliferation but become a vulnerability in quiescence, potentially offering new and selective therapeutic targets in nondividing cells (Fendt *et al*, 2020).

Fatty acid beta‐oxidation has so far mostly been studied in specific cell types with a focus on its role in energy generation (Schafer *et al*, 2009; Ito *et al*, 2012; Knobloch *et al*, 2017; Shyh‐Chang & Ng, 2017) or redox homeostasis (Kalucka *et al*, 2018). Our results shed light on the multiple implications of fatty acid degradation functionality for cellular homeostasis and in mediating the reversible transition between proliferation and quiescence. Here, we show that the approved drug trimetazidine, inhibitor of FAO‐enzyme 3‐keto acyl‐CoA thiolase, whilst showing no activity against rapidly proliferating cells, is able to induce apoptosis in quiescence‐induced cells. Our metabolome‐based analysis suggests that interfering with fatty acid β‐oxidation using agents like trimetazidine can lead to the formation of bioactive signaling species such as apoptosis‐inducing ceramides, and ultimately cell death. We also observed increased levels of phospholipids containing long‐chain fatty acyls, and of phosphatidic acid, a central precursor of major classes of membrane phospholipids. While these types of phospholipids exert no known key signaling function, it is plausible that when FAO is impaired, long‐chain fatty acids are redirected for incorporation into phospholipids by *de novo* synthesis and/or remodeling of membrane phospholipids to avoid lipotoxicity induced by free or activated fatty acids (e.g., fatty acyl‐CoAs) (Ostrander *et al*, 2001; Penzo *et al*, 2002; Listenberger *et al*, 2003; Artwohl *et al*, 2009; Piccolis *et al*, 2019). Interfering with FAO using trimetazidine also impairs the ability of quiescence‐induced cells to reverse growth arrest, suggesting that following cell fate after treatment can offer new mechanistic insights and uncover modulators of cell state transitions.

While verification in *in vivo* models is needed to explore a potential therapeutic use of our findings, genome‐based investigations have demonstrated that metabolic shifts like enhanced FAO can be decisive for cell fate and proliferation *in vivo* (Echeverria *et al*, 2019; Oren *et al*, 2021). It is plausible that metabolic processes mediating homeostasis or cellular stress responses could similarly be key to the ability of quiescent cells to tolerate and resist treatments (Ramirez *et al*, 2016; Brown *et al*, 2017). Our approach, by uncovering common metabolic adaptations in cancer cell quiescence, offers new perspectives on the conventional search for chemotherapies based on the growth inhibitory activity against rapidly dividing cancer cells, leading to new strategies for the discovery of therapeutic agents against difficult‐to‐target nondividing cancer cell subpopulations. Obviously, it is crucial that agents interfering with essential metabolic processes in quiescence preserve healthy human quiescent cells such as immune, stem, or endothelial cells. Nevertheless, the presented findings unraveled the potential of exploring whether general medications with no significant adverse effects, like the anti‐ischemic therapeutic agent trimetazidine (Fragasso *et al*, 2003; Napoli *et al*, 2005), could be repurposed to selectively target quiescent cancer cells and potentially resensitize them to classical chemotherapeutic agents. Together with conventional anticancer agents, combination therapies (e.g., with trimetazidine) could simultaneously target highly proliferating and quiescent cancer cells, and thereby reduce the risk of cancer recurrence. Understanding the mechanisms that govern the ability to switch between proliferation and quiescence can shed light on fundamental aspects of tissue homeostasis, immune cell maintenance, and activation and have implications in largely diverse applicative fields, from tissue engineering to pharmacology.

## Materials and Methods

### Cell lines and cell cultivation

Six human‐derived cell lines were used in this study, four cancer cell lines A549, HCT116, MCF7, and SKOV3 (obtained as part of the NCI‐60 panel from the National Cancer Institute, Bethesda, MD, USA), and two nontransformed fibroblast cell lines CCD1070Sk and HFL1 (purchased from ATCC). The standard growth medium for all cell lines was RPMI‐1640 (cat. no. 01‐101‐1A, Biological Industries, Israel or cat. no. 21870076, Thermo Fisher Scientific) supplemented with 2 g/l glucose (as necessary, cat. no. G8644, Sigma Aldrich, Buchs, Switzerland), 2 mM glutamine (cat. no. 25030024, Thermo Fisher Scientific), 1% penicillin–streptomycin (P/S, cat. no. 15140122, Thermo Fisher Scientific) and 5% dialyzed fetal bovine serum (dFBS, cat. no. F0392, Sigma Aldrich, Buchs, Switzerland). Cultures were routinely tested for Mycoplasma contamination and were found contamination‐free.

### Quiescence induction

For serum starvation and glutamine limitation, cells were seeded below confluence in normal growth medium (RPMI‐1640 with 2 g/l glucose, 2 mM glutamine, 1% P/S, and 5% dFBS) and allowed to attach overnight. To induce quiescence via serum starvation, the medium was then changed to RPMI‐1640 with 2 mM glutamine and 2 g/l glucose but without serum supplementation for 96 h. For glutamine limitation, the medium was changed to RPMI‐1640 with 2 g/l glucose and 5% dialyzed FBS but without glutamine supplementation, for 96 h. For contact inhibition, cells were seeded already close to confluence and allowed to attach overnight. The medium was then renewed once (RPMI‐1640, 2 g/l glucose, 2 mM glutamine, and 5% dFBS). All quiescence‐induced cell cultures were incubated for 96 h at 37°C in 5% CO_2_ atmosphere.

### Continuous cell growth monitoring

To monitor cell numbers and growth in proliferating, quiescence‐induced (serum starvation or glutamine limitation), or quiescence‐exiting cells *in situ* in 96‐well plates, we used automated time‐lapse microscopy imaging in a plate reader to measure cell confluence, i.e., the area of the well bottom covered by cells. Using a TECAN Spark 10 M plate reader, we acquired bright‐field microscopy images of each well in 1.5‐h intervals in standard cell culture conditions (5% CO_2_ and 37°C). Images were analyzed and confluence estimated online in SparkControl software (TECAN, Männedorf, Switzerland). Confluence data during quiescence induction and subsequent regrowth are provided in Dataset EV2.

### Estimation of growth rates and lag times

To estimate dynamic growth rates in each growth phase (e.g., between quiescence induction and stimulation), we used a moving window approach following an exponential growth model. First, we log‐transformed cell confluence data and performed linear regression analysis for 24‐h time windows (i.e., 16 consecutive data points) to determine the slope, representative of the log_10_ change in confluence per unit of time, i.e., growth rate with the unit h^−1^. Next, we shifted the time window by 8 h and repeated the analysis, thus iteratively scanning the entire growth curve and recording instantaneous growth rates (Dataset EV2). To estimate lag times, i.e., the duration between stimulation until the onset of growth with maximal rate, we calculated the time‐point at which the tangent to the growth curve at the maximum growth rate intersects with the initial confluence, using the slope and intercept determined by linear regression of log‐transformed confluence data in the time window where the highest growth rate is observed (Dataset EV2).

### Cell cycle analysis using flow cytometry

To determine cell cycle distributions and G_0_ fractions in cell cultures exposed to quiescence stimuli, we used a well‐established flow cytometry assay (Kim & Sederstrom, 2001; Lemons *et al*, 2010; Hu *et al*, 2011) based on the quantification of single‐cell DNA and RNA contents. In brief, live cell cultures growing adherently in T75 flasks were detached using trypsin, resuspended in warm PBS (pH 7.4), and centrifuged at 400 g for 5 min. After discarding the supernatant, the cell pellets were resuspended in 1 ml PBS and fixed by drop‐wise addition of the cell suspension into 10 ml of a 70% ethanol/30% deionized water mixture (precooled to −20°C) in a 50 ml conical tube while vortexing. Samples were stored at 4°C until further processing. Immediately prior to flow cytometry analysis, the fixed cell suspension was centrifuged at 1,200 *g* for 15 min at 8°C. The supernatant was carefully aspirated, and the cell pellet was resuspended in 5 ml cold PBS and centrifuged again at 1,200 *g* for 15 min. The cell pellet was then resuspended in 500 μl cold PBS and mixed with a freshly prepared staining solution containing 4 μg/ml Hoechst 33342 (stains DNA) and 4 μg/ml pyronin Y (stains RNA) in PBS. Samples were incubated in the dark on ice for at least 15 min until flow cytometry analysis. The stained cell samples were analyzed using a BD LSRFortessa flow cytometry analyzer, using 405 and 561 nm lasers for excitation, and 450/40 and 586/15 emission filters for Hoechst 33342 and pyronin Y, respectively. At least 10.000 events were recorded in each sample with a flow rate of 12 μl/min. The measured data (Dataset EV1) were visually inspected in the instrument software and exported to fcs 3.0 file format. For further analysis, flow cytometry data were imported in Matlab using the function *fca_readfcs* (https://www.mathworks.com/matlabcentral/fileexchange/9608‐fca_readfcs). Cell cycle distributions and G_0_ fractions were determined using a custom script as follows (see also Appendix Fig S1). Samples collected at 8 h after quiescence induction (containing cells in all cell cycle phases) were used to define the gates separating the cell cycle phases in each cell line (Fig 1 and Appendix Fig S1). G_1_ and G_2_/M peaks (2n and 4n DNA contents, respectively) were picked based on DNA content measurements (Hoechst 33342 intensity) using the Matlab function *findpeaks*. Cells with DNA contents between G_1_ and G_2_/M phases were classified as S‐phase cells (Appendix Fig S1). Cells with 2n DNA content and an RNA content below S and G_2_/M phases are classified as G_0_ (Kim & Sederstrom, 2001) (Fig 1, Dataset EV1).

### Metabolome analysis after trypsin‐mediated cell detachment

To assess the metabolic impact of enzymatic cell detachment (e.g., during flow cytometry workflows), we generated cell extracts using two different methods (see schematic overview in Appendix Fig S2), i.e., with or without prior trypsin treatment to digest extracellular matrix components that mediate the attachment of cells to the culture dish. To that end, we seeded four different cell lines, i.e., A549, IGROV1, OVCAR8, and UO31 cancer cell lines, in 6‐well plates to achieve approximately 50% confluence after 24 h (1.32e5, 1.80e5, 1.32e5 and 1.26e5 cells/well, respectively). Approximately 24 h after seeding, cells in three replicate cultures (i.e., containing identical cell numbers) of each cell line were extracted with or without prior cell detachment as follows. For cell detachment, the culture medium was aspirated, and cells were briefly rinsed with 2 ml warm phosphate‐buffered saline solution (PBS, pH 7.4, 37°C, cat. no. 10010023, Thermo Fisher Scientific). After removing the wash solution, 500 μl trypsin solution (0.25%, cat. no. 25200056, Thermo Fisher Scientific) was added to each well, and the cultures were incubated at 37°C in 5% CO_2_ atmosphere until cells were detached (approximately 5 min). Detached cells were subsequently resuspended in 2 ml cell culture medium containing 5% serum to inactivate trypsin, transferred to separate sample tubes, and immediately centrifuged at 400 g for 5 min at room temperature. Cell pellets were washed once with 2 ml warm 75 mM ammonium carbonate solution (pH 7.4, 37°C) and centrifuged as before. Finally, the wash solvent was discarded, and cells were extracted by adding 400 μl precooled extraction solvent (40% acetonitrile, 40% methanol, 20% water, with 25 μM phenyl hydrazine) to each tube. The samples were incubated for 1 h at −20°C, and subsequently stored at −80°C until MS analysis. In parallel, three replicate cultures of each cell line were extracted by *in situ* metabolite extraction as described previously (Dubuis *et al*, 2018; Ortmayr *et al*, 2019), i.e., culture media were removed, each well briefly washed with 2 ml prewarmed 75 mM ammonium carbonate solution, and the still attached cells were then directly extracted by addition of 400 μl of cold extraction solvent (40% acetonitrile, 40% methanol, 20% water, with 25 μM phenyl hydrazine). Plates were sealed with aluminum adhesive foil (cat. no AB0626, Thermo Fisher Scientific), incubated for 1 h at −20°C, and subsequently stored at −80°C. Immediately before MS analysis, the bottom of each well was scraped with a cell culture scraper, and the extract with cell debris was quantitatively transferred to a fresh sample tube. Together with the samples obtained from trypsin‐mediated cell detachment, all extracts were centrifuged to separate cell debris (5 min, 14,000 rpm). Cell‐free extracts were then transferred to fresh 96‐well plates with a conical bottom for MS injection. MS analysis and metabolite annotation was carried out as described below (section “Sample mixing and MS analysis”). MS intensity data are provided in Dataset EV3. To compare metabolic profiles between trypsin‐mediated cell detachment and *in situ* extraction, measured ion intensities were transformed to Z‐scores and analyzed by principal component analysis. In addition, to account for potential systematic differences in effectively extracted cell numbers (e.g., due to loss of detached cells during centrifugation steps prior to extraction), we repeated the analysis after centering metabolite intensities to the mean for each extraction method separately (Appendix Fig S2, Dataset EV3).

### Evaluation of linearity of MS measurements

To evaluate the linearity of MS signals measured in cell extract samples, we supplemented cell extract samples with increasing concentrations of endogenous metabolites and measured MS signal intensities using FIA‐TOFMS (Fuhrer *et al*, 2011). To that end, we generated cell extracts of three different cell lines (i.e., A549, IGROV1, and MDAMB468 cancer cells), representing three potentially different sample matrices, and, for each cell line separately, mixed 25 μl extract with increasing amounts of 105 metabolites (equimolar mixture prepared from pure standards) ranging from 3 pmol to 3 nmol in a fixed volume of 5 μl. In parallel, metabolites were spiked at the same concentrations into cell‐free extraction solvent. All spiked samples were prepared in triplicates and immediately measured by FIA‐TOFMS.

Following metabolite annotation based on exact mass (3 mDa mass tolerance, 83 unique ions), we first determined the saturation limit for each annotated ion, i.e., the ion abundance above which a further increase in metabolite concentration no longer yields an increase in measured ion abundance (examples in Appendix Fig S3), due to ion suppression effects and/or saturation of the MS detector. Ions whose abundance was close to or above the saturation limit already in the unspiked cell extract samples were excluded from further analysis (13 ions). For all remaining spiked metabolites (75 unique ions, Appendix Fig S3), we evaluated the linearity of MS signals in each of the four sample matrices (i.e., three cell lines or cell‐free extract) as follows.

First, we asked whether the increase in MS ion intensity was linear with respect to the spiked metabolite concentration. To that end, for each sample matrix separately, we performed linear regression analysis on measured ion intensities to establish a base model that determines the slope of the linear relationship between ion intensity and the spiked metabolite concentration, and the intercept relating to the basal abundance of the metabolite in the cell extract (Dataset EV3). Measurements above the saturation limit were excluded from the analysis, allowing the fitting of a linear model across at least two orders of magnitude of ion intensity beyond the ion intensity in the unspiked cell extract for each metabolite (Appendix Fig S3, Dataset EV3).

In the second step, to test whether the same model also fits measured ion intensities in different sample matrices, we compared the predicted ion intensities in the base model to the measured ion intensity in all other sample matrices. Because 31 metabolites exhibited a cell‐type‐specific abundance in the unspiked extracts (1‐way ANOVA, adjusted *P*‐value <0.05, Benjamini–Hochberg multiple testing correction, Appendix Fig S3), we adjusted the intercept parameter to each sample matrix, while the slope parameter was fixed to the value determined in the base model. We then calculated *R* ^2^ values to quantify the deviation of model predictions from the actual MS measurements in each sample matrix (goodness of fit, Appendix Fig S3, Dataset EV3), i.e., the ratio of variance explained by the model (i.e., deviation from the model prediction) and the total variance (i.e., deviation from the mean). *R* ^2^ values close to 1 indicate an excellent fit, i.e., that the linear model explains most of the variance observed in the measured data. We repeated this linearity comparison for each sample matrix acting as the base model and applied each base model to all three other sample matrices (Appendix Fig S3 and Dataset EV3).

### Sample generation for metabolome profiling in G_0_ cells

Cell extracts of quiescence‐induced cultures at 8 and 96 h exposure to the quiescence stimulus were generated in 6‐well plates to increase the sample volume for later preparation of mixed samples. Five 6‐well plates were prepared in each experiment, with 2 cell lines in three replicates each. At 8 and 96 h after induction of quiescence, an aliquot of the culture supernatant was removed and stored at −80°C, and the remainder was aspirated. Each well was subsequently washed once with fresh 75 mM ammonium carbonate (wash solvent, 37°C, pH 7.4), and metabolites were extracted from adherent cells *in situ* with 400 μl cold extraction solvent (40:40:20 acetonitrile:methanol:water with 25 μM phenyl hydrazine for the stabilization of α‐keto acids (Zimmermann *et al*, 2014)). The plates were sealed with aluminum adhesive foil, kept at −20°C for 1 h, and then stored at −80°C until further processing. A second plate was used to determine the cell number per well for later normalization. In each well, the medium was aspirated, cells were washed once with warm wash solvent and detached with 250 μl trypsin (0.25%, cat. no. 25200056, Thermo Fisher Scientific). Immediately after resuspending cells in 750 μl warm PBS, equal volumes of cell suspension and trypan blue solution (0.4%, Invitrogen) were mixed, and the cell concentration, average cell size, and viability were determined using a Countess II Automated Cell Counter (Invitrogen, Thermo Fisher Scientific).

Of note, for contact‐inhibited cultures, the samples at 8 h (i.e., the reference sample containing mostly proliferating cells in G_1_, S, or G_2_/M phases) were collected from subconfluent cultures incubated with spent medium from a fully confluent culture for 8 h, to mimic the acute exposure to low‐nutrient conditions.

In addition, we generated metabolite extracts for each cell line grown in normal growth conditions (i.e., subconfluent culture, standard growth medium with both dFBS and glutamine) at several time‐points between 20 and 80% cell confluence. These samples were used for the selection of ions of likely biological origin in our data analysis and deconvolution procedure (see below).

### Sample mixing and MS

Immediately prior to MS analysis, the 6‐well plates holding cell extracts were briefly thawed on ice, and cells were detached from the well bottom using cell scrapers (cat. no. 3010, Corning). All subsequent steps were carried out on ice. The extracts were collected in fresh microcentrifuge tubes (cat. no. 0030120086, Eppendorf), mixed, and centrifuged at maximum speed (13,000 rpm in an Eppendorf 5424 R microcentrifuge) to deposit cell debris. The supernatant, i.e., the cell‐free metabolite extracts, was used for subsequent sample mixing and MS measurements. In brief, for each cell line and quiescence stimulus, the triplicate extracts obtained at 8 (Extract 1) and 96 h (Extract 2) were adjusted to the same average cell concentration and mixed at eight defined ratios, i.e., 100, 85, 70, 55, 40, 25, 10, 0% of Extract 1 in Extract 2, in a total volume of 50 μl. The mix samples were prepared independently for the three replicates in 96‐well microtiter plates. The metabolite extracts generated from cell cultures in normal growth conditions at different levels of cell confluence were processed as described above, but no mixing was applied. Empty wells on each plate were filled with fresh (i.e., cell‐free) extraction solvent or quality control samples, then plates were sealed and stored at 4°C until injection.

MS measurements were performed as described previously (Fuhrer *et al*, 2011) by flow‐injection analysis time‐of‐flight mass spectrometry (FIA‐TOFMS) on an Agilent 6550 iFunnel Q‐TOF LC–MS system (Agilent Technologies, Santa Clara, CA, USA). Raw MS spectra were aligned and centroids picked using in‐house data processing environment in Matlab R2018b (The Mathworks, Natick), yielding MS intensities for typically more than 10,000 unique *m/z* features in each sample.

### Metabolite annotation

The detected *m/z* features were putatively annotated to known metabolites by matching accurate masses to metabolites listed in the genome‐scale reconstruction of human metabolism (Brunk *et al*, 2018) (Recon3D, 5835 metabolites) and the human metabolome database (HMDBv4; Wishart *et al*, 2018). Here, we putatively annotated 2,099 measured ions with a mass tolerance of 0.003 *m/z*. Sum formulae were used to calculate reference masses for 5,835 and 7,038 metabolites listed in Recon3D and HMDBv4 (subset of endogenous metabolites in urine, serum, feces, excluding drugs), respectively. Because α‐keto acids were derivatized with phenyl hydrazine (Zimmermann *et al*, 2014) during extraction, sum formulae for the phenyl hydrazone derivatives (+C_6_H_8_N_2_‐H_2_O) of 30 α‐keto acid compounds (selected via KEGG SimComp search, http://www.genome.jp/tools/simcomp/) were added to the metabolite list for annotation (marked by the prefix “PHderiv_” in Dataset EV4 and by the suffix “[d]” in figure labels).

### Regression‐based deconvolution

We designed a two‐step procedure to analyze and deconvolute the metabolic profiles of cells in G_0_ as compared to any other cell cycle phase. Of note, most cells in the non‐G_0_ fraction resided in G_1_ phase (Fig 1).

In the first step, we selected only ions where the measured ion intensity is linearly dependent on the number of cells extracted, i.e., that are likely of biological origin. To that end, we adapted an approach we previously described in Ortmayr *et al*, 2019 for the comparative metabolic profiling of widely different cancer cell lines. During steady‐state growth, the metabolite concentration in each cell is constant in time, and the measured ion intensity of intracellular metabolites scales with the number of cells extracted. Ion signals not following this relationship are likely artifacts not related to the intracellular metabolic content or represent metabolites yielding ion abundances below the detection limit. Following this criteria, for each putatively annotated metabolite, we analyzed the ion intensities obtained in metabolite extracts of each cell line grown in normal growth conditions at different confluence levels (i.e., different amounts of cells extracted, see above for sample generation) using a multiple linear regression scheme (Matlab *fitlm* function) as described in detail in (Ortmayr *et al*, 2019). For each cell line, we obtained the slope and significance (*P*‐value) of the linear fit, and one value for the intercept, estimated from the measured intensity in a sample where no cells were extracted, and hence representative of the MS background signal independent of the cell line. Only putatively annotated metabolites with a regression *P*‐value below 3.97e‐06 (Bonferroni‐adjusted threshold, adjusted by the number of metabolites and cell lines) in at least one cell line were retained after this step.

In the second step, we used a multilinear regression scheme to estimate the relative metabolite abundance in G_0_ and non‐G_0_ cells in each cell line and quiescence‐inducing condition based on the measured ion intensities in mixed cell extracts (see above). While this approach can be in principle applied to estimate relative metabolic differences across multiple cell subpopulations, here we describe the approach for cell populations consisting of two main subpopulations. Key to this approach is to prepare samples in which the two different subpopulations are present in different ratios. The total measured ion intensity across samples can be expressed as a linear combination of metabolite concentrations in the two subpopulations. Specifically, ion intensities measured in mixed cell extract samples, $I_{\mathrm{mix}}$ can be expressed as:

(1) $$I_{\mathrm{mix}}=\alpha_{\mathrm{pop}1}\cdot N_{\mathrm{pop}1}\cdot V_{\mathrm{pop}1}+\alpha_{\mathrm{pop}2}\cdot N_{\mathrm{pop}2}\cdot V_{\mathrm{pop}2}+\beta ,$$

where β is a constant term representing the ion‐specific MS measurement background, $N_{\mathrm{pop}1}$ and $N_{\mathrm{pop}2}$ are the number of cells in either subpopulation in the mixed sample, and $\alpha_{\mathrm{pop}1}$ and $\alpha_{\mathrm{pop}2}$ are the parameters fitted in the model, representing the actual metabolite abundance in each subpopulation. By comparing the $\alpha_{\mathrm{pop}1}$ vs $\alpha_{\mathrm{pop}2}$ we can quantify relative differences in intracellular metabolite abundances between the two subpopulations. $V_{\mathrm{pop}1}$ and $V_{\mathrm{pop}2}$ are the cell volume of cells in the respective subpopulation. Here, we considered the volumes of cells equal, because flow cytometry‐based estimates of cell volume forward‐scatter (Tzur *et al*, 2011) indicated a characteristic difference in cell diameter between G_0_ and non‐G_0_ cells in any given cell line and quiescence stimulus (Appendix Fig S1).

Here, we applied this general model to deconvolute the relative metabolite abundances in G_0_ and non‐G_0_ cells (i.e., G_1_, S, or G_2_/M phase, Fig 1) in six cell lines exposed to three different quiescence‐inducing stimuli. According to the above‐described mixing scheme, for each cell line and quiescence stimulus, we obtained triplicate ion intensity measurements in eight different mix samples. The total number of cells is constant in all mix samples, and the variable number of cells in G_0_ (*N*_*Q*_) or any other cell cycle phase (N_P_) is given by the G_0_ fractions determined using flow cytometry (assay described above) at 8 and 96 h after exposure to the quiescence stimulus, respectively.

Here, for each putatively annotated metabolite retained in step 1, we solved the basic model described in Equation (1) across all cell lines and quiescence stimuli all at once using a multilinear regression scheme (Matlab *lsqlin* function with model coefficients constrained from 0 to Inf):

(2) $$\left[\begin{array}{c} I_{{\mathrm{mix}}_{1},1,1,1}\\ I_{{\mathrm{mix}}_{1},1,1,2}\\ I_{{\mathrm{mix}}_{1},1,1,3}\\ \ldots \\ I_{{\mathrm{mix}}_{1},1,2,1}\\ I_{{\mathrm{mix}}_{1},1,2,2}\\ I_{{\mathrm{mix}}_{1},1,2,3}\\ \ldots \\ I_{{\mathrm{mix}}_{m},\mathrm{cl},\mathrm{st},n} \end{array}\right]-\beta =\begin{array}{l} \left[\begin{array}{ccccccc} N_{1,1,1,P,1} & N_{1,1,Q,1,1} & 0 & 0 & \ldots & 0 & 0\\ N_{1,1,1,P,2} & N_{1,1,Q,2} & 0 & 0 & \ldots & 0 & 0\\ N_{1,1,1,P,3} & N_{1,1,Q,3} & 0 & 0 & \ldots & 0 & 0\\ \ldots & \ldots & \ldots & \ldots & \ldots & \ldots & \ldots \\ 0 & 0 & N_{1,2,P,1} & N_{1,2,Q,1} & \ldots & 0 & 0\\ 0 & 0 & N_{1,2,P,2} & N_{1,2,Q,2} & \ldots & 0 & 0\\ 0 & 0 & N_{1,2,P,3} & N_{1,2,Q,3} & \ldots & 0 & 0\\ \ldots & \ldots & \ldots & \ldots & \ldots & \ldots & \ldots \\ 0 & 0 & 0 & 0 & \ldots & N_{\mathrm{cl},\mathrm{st},m,P,n} & N_{\mathrm{cl},\mathrm{st},m,Q,n} \end{array}\right]\\ \cdot \left[\alpha_{1,1,P}\;\alpha_{1,1,Q}\;\alpha_{1,2,P}\;\alpha_{1,2,Q}\;\ldots \;\alpha_{\mathrm{cl},\mathrm{st},P}\;\alpha_{\mathrm{cl},\mathrm{st},Q}\right], \end{array}$$

The ion intensity in the mixed sample, *I*_*mix*_, the metabolite‐specific MS background signal, β (i.e., the constant term in Equation (1)), and the number of cells in G_0_ or any other cell cycle phase, *N*_*Q*_ and *N*_*P*_, respectively, are measured, while the *α* coefficients are fitted. The index *cl* indicates the cell line, *st* the quiescence stimulus, *m* the sample mix (1–8 with predefined mixing ratios, see above), and *n* the replicate (1–3). For each metabolite, the model coefficients α_*cl,st,P*_ and α_*cl,st,Q*_ represent the relative intracellular abundance of the given metabolite. To estimate the error for each coefficient, we used a bootstrap resampling approach, where we first calculated the model residuals (i.e., the deviation of the model prediction from the measured values) for each subpopulation, and then selected a random sample of the residuals with replacement and added it to the model prediction. Using linear regression analysis, we then obtained new coefficient values α_*cl,st,P*_ and α_*cl,st,Q*_. After 100 repetitions of these steps, we calculated the errors of the model coefficients, ${\mathrm{SD}}_{\mathrm{cl},\mathrm{st},P}$ and ${\mathrm{SD}}_{\mathrm{cl},\mathrm{st},Q}$, as the standard deviation of the bootstrapped coefficients. Using this approach, we obtained estimates of the relative intracellular abundance (coefficients α) and their standard errors separately for G_0_ and non‐G_0_ cells in each cell line and quiescence stimulus (i.e., 2‐cell states × 6‐cell lines × 3 quiescence stimuli = 36 observations per metabolite), for 1,940 putatively annotated metabolites (Dataset EV4).

### Method validation

To verify the ability of the deconvolution workflow to disentangle the metabolic signatures of two distinct cell subpopulations, we tested whether known differences in metabolite abundance between two cell lines can be recovered after applying the deconvolution strategy described above. To that end, we generated cell extract samples from individual cultures of five cell lines in standard growth conditions (RPMI‐1640 with 2 g/l glucose, 2 mM glutamine, 1% P/S, and 5% dFBS), and calculated the cell concentration in the extract samples. Using the mixing scheme described above, we prepared eight mixed samples for each possible pair of cell lines, and measured metabolite abundances using FIA‐TOFMS as described above. After annotation, we applied the deconvolution approach (Equation (1)). For each cell line, we determined one model coefficient α and its error for 2,713 putatively annotated metabolites (Dataset EV3). Next, we compared these estimates of relative metabolite abundances across the five cell lines with existing data. We previously reported comparative metabolic profiles under steady‐state conditions for the same 5 cell lines as part of a larger panel of 54 adherent cancer cell lines (Ortmayr *et al*, 2019) (NCI‐60 panel). In that dataset, 267 out of 2,181 putatively annotated metabolites were characteristically different between the five cell lines, i.e., showing a variation across cell lines greater than three times the typical error of the estimates for individual cell lines. Of these metabolites showing the most characteristic differences between the cell lines, 117 were also annotated in the herein‐generated dataset after deconvolution. To systematically assess the overlap of the relative metabolite abundances for these key metabolites, we calculated Spearman correlation coefficients (Fig 2C), confirming that characteristic differences in the metabolic profiles of the five cell lines were recovered.

### Calculation of purified ion intensity profiles of G_0_ and non‐G_0_ cells

In addition to obtaining the model parameters quantifying the characteristic relative metabolite abundance in G_0_ vs. non‐G_0_ cells (see above, Regression‐based deconvolution), we also reconstructed ion intensity profiles in the original cell extracts at 8 and 96 h (corresponding to mostly non‐G_0_, and mostly G_0_ cells, respectively), similar to the approach used in a previous study (Wang *et al*, 2016). To that end, we used the previously determined slope parameters from the regression model together with the known subpopulation fractions to calculate and subtract for each annotated metabolite the ion intensity attributed to the minor subpopulation, hence obtaining a purified intensity profile corresponding to only one subpopulation. For example, in the sample taken at 96 h, enriched for cells in G_0_, we multiplied the slope value for non‐G_0_ cells ($\alpha_{\text{non}-G_{0}}$) by the number of non‐G_0_ cells ($N_{\text{non}-G_{0}}$) and subtracted this intensity value from the measured ion intensity $I_{\mathrm{mix}}$, such that:

(3) $$I_{G_{0}}=I_{\mathrm{mix}}-\alpha_{\text{non}-G_{0}}\cdot N_{\text{non}-G_{0}}\cdot V_{\text{non}-G_{0}}$$

Vice versa, the intensities measured in extracts obtained at 8 h, containing mostly non‐G_0_ cells, were corrected for the ion intensity predicted to originate from the small subpopulation of G_0_ cells.

(4) $$I_{\text{non}-G_{0}}=I_{\mathrm{mix}}-\alpha_{G_{0}}\cdot N_{G_{0}}\cdot V_{G_{0}}$$

Similar to the original model, the cell volumes $V_{G_{0}}$ and $V_{\text{non}-G_{0}}$ were assumed to be constant (see also above). Importantly, this approach to separate metabolic signatures of cell subpopulations does not correct for differences in cell numbers, hence we additionally normalized the purified ion intensity profiles (Dataset EV4) to the number of non‐G_0_ cells (for the 8‐h sample) or G_0_ cells (for the 96‐h sample). All calculations and the subsequent analysis by principal component analysis (PCA, Appendix Fig S3) were carried out in Matlab 2019b. Of note, because the parameters $\alpha_{\text{non}-G_{0}}$ and $\alpha_{G_{0}}$ are as per model definition invariant to cell numbers and are derived across multiple independent ion intensity measurements (i.e., 8 different mixing ratios) rather than individual measurements of end‐point samples, all other analyses presented in this paper are obtained based on $\alpha_{\text{non}-G_{0}}$ and $\alpha_{G_{0}}$ as measures of the characteristic relative abundances of intracellular metabolites in non‐G_0_ and G_0_ cells, respectively.

### Differential analysis: effect size

To systematically compare the metabolite abundances between G_0_ and any other cell cycle phase, we calculated effect sizes d for each annotated metabolite *i*, i.e., the difference in relative metabolite abundances *α* in matching cell types *cl* and quiescence‐triggering conditions *st* standardized to the pooled standard deviation (*SD*) of metabolite abundances:

(5) $$d_{i,\mathrm{cl},\mathrm{st}}=\frac{\alpha_{i,\mathrm{cl},\mathrm{st},Q}-\alpha_{i,\mathrm{cl},\mathrm{st},P}}{{\mathrm{SD}}_{i,\mathrm{cl},\mathrm{st},\text{pooled}}}$$

(6) $${\mathrm{SD}}_{i,\mathrm{cl},\mathrm{st},\text{pooled}}=\sqrt{\frac{{{\mathrm{SD}}_{i,\mathrm{cl},\mathrm{st},P}}^{2}+{{\mathrm{SD}}_{i,\mathrm{cl},\mathrm{st},Q}}^{2}}{2}},$$

where ${\mathrm{SD}}_{i,\mathrm{cl},\mathrm{st},P}$ and ${\mathrm{SD}}_{i,\mathrm{cl},\mathrm{st},Q}$ are the errors in the estimates of relative metabolite abundances determined above. A negative effect size indicates a lower abundance of a metabolite in quiescent cells, while positive values reflect an accumulation of metabolites in quiescent cells. The result of this analysis is differential metabolic profiles in quiescent vs. proliferating cells for 1,940 putatively annotated metabolites in six cell types and three quiescence trigger signals (Dataset EV4).

### Analysis of common metabolic changes in G_0_ cells

To identify metabolites that show a consistent pattern of differences between G_0_ phase and any other cell cycle phase (i.e., effect sizes calculated as described above) we used an iterative thresholding approach. The basic principle behind the analysis is to assess how strongly and significantly any given metabolite is associated with G_0_ state, based on how consistently and similarly the metabolite abundance changes between G_0_ and non‐G_0_ cells across 6 cell lines and 3 quiescence‐inducing stimuli. To avoid selecting a single threshold value for all metabolites and for both increasing and decreasing metabolite abundances (i.e., positive and negative effect sizes), we assessed the association with G_0_ for different effect size thresholds between 3 and 13 (the mean of all absolute effect size values) in increments of 1.

For each metabolite and effect size threshold, we first counted separately the number of effect sizes that exceed the positive threshold value, and the number of effect sizes that are lower than the negative threshold value. The sign with the larger number of changes is then prioritized, i.e., the metabolite is assigned either a consistently accumulating or decreasing pattern at that threshold value. To assess the statistical significance of such a consistent pattern at each threshold value, we used a hypergeometric test. Here, we assessed the probability of drawing the same or higher number of effect sizes exceeding the threshold value (for accumulated metabolites) or falling below the threshold value (for metabolites with decreased abundance in G_0_ cells) when sampling at random from all changes in the dataset with matching sign. Thus, at each threshold value, we recorded the sign and fraction of changes passing the threshold (out of 18 in total, i.e., 6‐cell lines × 3 quiescence stimuli), and the statistical significance (*P*‐value) of a common pattern for each metabolite. To define an overall G_0_‐associated metabolic signature, for each metabolite we selected the threshold value where the most significant common pattern (i.e., lowest *P*‐value) was observed. The resulting profile of G_0_ associations and *P*‐values (Benjamini–Hochberg correction for multiple hypothesis testing) for 1940 putatively annotated metabolites are reported in Fig 2F and Dataset EV4.

### Inhibition of fatty acid β‐oxidation

We used two different pharmacological inhibitors of fatty acid β‐oxidation (FAO), i.e., trimetazidine and etomoxir. While trimetazidine competitively inhibits 3‐keto acyl‐CoA thiolase, i.e., directly targets the last enzymatic step of FAO, etomoxir (in its active form etomoxiryl‐CoA) is an irreversible inhibitor of carnitine palmitoyl transferase (CPT1), thus inhibiting FAO indirectly by limiting the substrate access into mitochondria. To treat adherent cancer cells, the drugs were added directly into the supernatant, either individually or in combinations, as indicated in the main text. Trimetazidine solutions were prepared by dissolving trimetazidine (1‐(2,3,4‐Trimethoxybenzyl)piperazine dihydrochloride, cat. no. 653322, Sigma Aldrich, Buchs, Switzerland) in deionized water to a concentration of 50 mM. The pH of the solution was adjusted to pH 7.4, and the solution was sterile‐filtered prior to addition to the culture supernatant. To establish the dose‐dependent effect of trimetazidine on serum‐starved cancer cells, we prepared serial dilutions (10 μM up to 1 mM) of the drug in serum‐free RPMI‐1640 with 2 g/l glucose, 2 mM glutamine, and 1% P/S. Growth phenotypic and metabolic changes in response to trimetazidine exposure in full medium and quiescence‐inducing conditions are reported in Dataset EV5 and EV6.

### Targeted analysis of trimetazidine‐induced metabolic changes by LC–MS/MS

HCT116 cells were seeded in two 6‐well plates and incubated overnight at 37°C in 5% CO_2_ atmosphere to allow cell attachment. On the next day, on one plate (i.e., six replicate cultures) the medium was exchanged for serum‐free medium, and trimetazidine was added to three wells at a final concentration of 1 mM. Similarly, on the second plate, the medium was removed and replaced by a fresh growth medium containing 5% dialyzed FBS, and 1 mM of trimetazidine was added to three wells. Serum‐starved cultures with or without trimetazidine were extracted at 72 h after treatment, while cultures in full growth medium were sampled before reaching confluence, at 48 h. Cell extracts were generated using *in situ* extraction (Dubuis *et al*, 2018; Ortmayr *et al*, 2019) as described above, and stored at −80°C until MS analysis. Immediately prior to MS analysis, 150 μl aliquots of all cell extracts were dried by vacuum centrifugation and resuspended in 30 μl (5‐fold concentration) LC–MS grade water.

LC–MS analysis was carried out on a Thermo TSQ Vantage triple quadrupole mass spectrometer, using a chromatographic method adapted from a previous publication (Lu *et al*, 2010), and MS/MS parameters established in Buescher *et al* (2010). Peak integration in LC–MS/MS data was carried out in Skyline, and statistical analysis was performed in Matlab 2019b (Dataset EV6).

### Viability imaging assay and CellProfiler analysis

To assess cell viability in adherent cell cultures *in situ*, we used a fluorescence microscopy assay based on live cell staining with the DNA‐binding dyes Hoechst 33342 and propidium iodide (PI). Hoechst 33342 is cell‐permeable and stains all cells (i.e., live and dead cells), while propidium iodide cannot enter live cells and stains only dead cells with damaged cell membranes. Hoechst 33342 (2 μg/ml in H_2_O, Life technologies cat. no. H3570) and propidium iodide (2 μg/ml, cat. no. P4864, Sigma Aldrich) were added directly into the supernatant of cell cultures in 96‐well plates, and the cells were incubated for 30 min at 37°C in 5% CO_2_ atmosphere before imaging. Without any prior washing steps, the cultures were imaged in 96‐well plates using a TECAN SparkCyto plate reader for fluorescence microscopy, recording images in the bright‐field, blue (Ex. 381–400 nm, Em. 414–450 nm) and red channels (Ex. 543–566 nm, Em. 580–611 nm). In each channel, multiple images were acquired of each well with 4× magnification and tiled online into a single image covering the whole well in the vendor software (TECAN SparkControl). The fluorescence images were analyzed in a custom image analysis pipeline in CellProfiler 3.1.9 (McQuin *et al*, 2018). For each well, the blue‐ and red‐channel images were first cropped into a circular area to exclude areas close to (200‐pixel border offset) and outside the well border and converted from RGB to gray scale. Next, both images were independently segmented to identify all nuclei (blue channel) and PI‐positive cells (red channel), respectively, using the *IdentifyPrimaryObjects* module of CellProfiler, and a global threshold estimated using Otsu's method (two classes). To improve the robustness of the automated threshold estimation, we constrained the lower bound for the threshold value in red channel images, thus preventing false‐positive recognition of dead cells in images where no or only few dead cells are present. In the last step, we applied the *MaskObjects* module to apply the PI‐positive cell mask to the segmented nuclei, i.e., nuclei for which a red object is recorded in the same location are recognized, and labeled as dead cells. As a measure of cell viability, we then calculate for each well the fraction of dead cells as the ratio of the number of dead cells (i.e., blue and red positive) over the total number of nuclei (blue objects), in % (Dataset EV6).

### Metabolite supplementation under trimetazidine treatment

To investigate the functional role of FAO in quiescence‐induced cells, we supplemented several small molecules together with trimetazidine, or during regrowth after trimetazidine treatment, i.e., citrate, etomoxir, and inosine (Dataset EV5). Citrate supplementation can bypass the reduced citrate production from FAO‐derived acetyl‐CoA upon FAO inhibition. Therefore, we supplemented citrate at a final concentration of 250 μM in cell culture media either at the same time as trimetazidine was added (0.25, 0.5, or 1 mM doses) or when regrowth was stimulated following 96 h serum starvation in presence of trimetazidine (0.25, 0.5, 0.75, or 1 mM doses). Citrate stocks were prepared from powder (citric acid monohydrate, cat. no. 1.00244.1000, Merck Millipore) at 50 mM in deionized water, and sterile‐filtered (0.25 μm pore size) prior to addition to cell culture media. We confirmed that at the final concentration of 200 μM, the pH of the cell culture medium (i.e., pH 7.4 under 5% CO_2_ atmosphere) was not affected by citrate addition.

Etomoxir is a small‐molecule inhibitor of carnitine palmitoyltransferase I (CPT1), and can thus limit the entry of fatty acyl chains into mitochondria and potentially divert fatty acid flux away from mitochondria, avoiding damage sustained by the accumulation of fatty acyls in mitochondria upon FAO inhibition. Etomoxir was prepared from powder ((+)‐Etomoxir sodium salt hydrate, cat. no. E1905, Sigma Aldrich, Buchs, Switzerland) as a 10 mM stock solution in DMSO. Because commonly used 40 μM doses have been associated with indirect effects (Divakaruni *et al*, 2018) rather than specific action on CPT1, we opted for a lower concentration, i.e., 10 μM.

Inosine is a purine metabolism intermediate and can serve as a source of inosine 5′‐monophosphate (IMP), adenosine 5′‐monophosphate (AMP), and guanosine 5′‐monophosphate (GMP). Here, inosine was used as a control condition to test the effect of a supplementation unrelated to FAO functions (in contrast to citrate, see above), and to test whether regrowth after serum starvation is generally metabolically limited by the production of key biosynthetic precursors like nucleotides (e.g., for nucleic acid synthesis). To that end, inosine was supplemented in fresh growth media when cells were stimulated to regrow after 96 h serum starvation with or without trimetazidine (0.25, 0.5, 0.75, or 1 mM doses). Inosine stock solutions were prepared from powder (cat. no. I4125, Sigma Aldrich, Buchs, Switzerland) at 10 mM in deionized water, and sterile‐filtered (0.25 μm pore size) prior to addition to cell culture media.

### Dynamic metabolome profiling under trimetazidine treatment

Time‐dependent changes in the abundance of individual metabolites were determined as described previously (Dubuis *et al*, 2018; Ortmayr *et al*, 2019). Cells were seeded in 96‐well plates in full growth medium (RPMI‐1640, 5% dialyzed FBS, 2 g/l glucose, and 2 mM glutamine). After allowing cells to attach overnight, media were replaced by serum‐free RPMI‐1640 in all wells to induce quiescence by serum starvation, and treatments were applied as indicated, i.e., trimetazidine was added to final concentrations of 400 μM or 1 mM from 50 mM stocks in RPMI‐1640 (pH adjusted to 7.4), with or without the addition of etomoxir at a final concentration of 10 μM from a 10 mM stock in DMSO. In experiments with cells in full medium, RPMI‐1640 with 5% dialyzed FBS was supplied. Samples for metabolome analysis were taken at 24‐h intervals after drug addition. During sampling, as described previously (Dubuis *et al*, 2018; Ortmayr *et al*, 2019), cells were washed once with 75 mM ammonium carbonate (pH 7.4, 37°C) and then immediately extracted *in situ* by adding 100 μl prechilled extraction solvent (40% methanol, 40% acetonitrile, 20% deionized water, −20°C) to each well without prior cell detachment. To estimate the number of cells in each sample, a second plate was processed as described above, but the medium was added instead of extraction solvent, and the plate was immediately imaged and analyzed for cell confluence on a TECAN Spark 10 M plate reader for automated bright‐field microscopy imaging. Extracted cell samples were incubated at −20°C for 1 h after extraction, and subsequently stored at −80°C until MS analysis. The MS‐based measurement of metabolite abundances was carried out as described above using untargeted high‐throughput flow‐injection analysis (Fuhrer *et al*, 2011) on an Agilent 6550 time‐of‐flight mass spectrometer. Metabolites were putatively annotated as described above, i.e., against Recon 3D and HMDBv4. In addition, all ions were matched to SwissLipids entries (https://www.swisslipids.org) via HMDB ids to classify lipid‐related metabolites and obtain sum composition information for putatively annotated lipids (Fig 3F–H and Dataset EV6). Metabolome profiles were normalized as described previously (Dubuis *et al*, 2018; Ortmayr *et al*, 2019), using a linear regression model to estimate the relationship between metabolite abundance and cell confluence for each putatively annotated metabolite at a steady state (i.e., in full medium). We then estimated the deviation of each metabolite's abundance from the expected steady‐state abundance by calculating the ratio of the measured abundance and the steady‐state abundance at the matching cell confluence based on the linear regression coefficients, as described in detail previously (Dubuis *et al*, 2018). In addition, we calculated the statistical significance (*P*‐value) of each change using an unpaired *t*‐test against all unperturbed samples. The resulting dataset of log_2_ fold‐changes in metabolite abundance and corresponding *P*‐values, also corrected for multiple hypothesis testing using the Benjamini–Hochberg method, is provided in Dataset EV6. For the data representation as a Volcano plot in Fig 3F, for each metabolite we subtracted the log_2_ fold‐change measured in serum starvation alone from the log_2_ FC values of trimetazidine‐treated cells, to highlight metabolites whose abundance was specifically altered by the added trimetazidine treatment. The statistical significance (*P*‐value) is estimated against the fold‐changes in the untreated quiescence‐induced condition, additionally applying the Benjamini–Hochberg correction for multiple hypothesis testing. In Fig 3G and H, we summarized the time‐dependent changes in abundance for each ion by taking the log_2_FC with the highest statistical significance (*P*‐value, *t*‐test against steady‐state abundance in full medium), i.e., the minimum of *P*‐values against full medium across all time‐points.

### Dynamic monitoring of apoptosis induction

We used a fluorescence imaging assay to assess the fraction of early and late apoptotic cells continuously in live cell cultures (Appendix Fig S8, Dataset EV6) under incubation conditions (37°C, 5% CO_2_) in a TECAN SparkCyto fluorescence imaging system. The assay included three different fluorescent probes, i.e., Hoechst 33342 (Life technologies cat. no. H3570) for staining the nuclei of live cells, ApoTracker Green (BioLegend, San Diego, CA, USA) to stain cells with phosphatidylserine (PS) residues exposed on the cell surface as a marker for early stages of apoptosis, and the live cell‐impermeable propidium iodide (Sigma Aldrich, cat. no. P4864) to stain cells with compromised cell membranes during late‐stage apoptosis or necrosis. Of note, in contrast to the related PS‐binding by Annexin V, binding by ApoTracker Green is calcium‐independent, allowing reliable staining also in calcium‐low cell culture media like RPMI‐1640. The concentration of Hoechst 33342 was optimized for each cell line (110 nM for A549, and 55 nM for HCT116) to minimize phototoxicity effects during long‐term live cell imaging. For ApoTracker Green and propidium iodide, the dye concentrations in the culture supernatant were 200 nM and 0.5 μg/ml, respectively.

For each experiment, cells were seeded as indicated in 96‐well plates (Nunclon™ Delta cell culture treated surface, Thermo Scientific, cat. no. 167008), and the three fluorescent probes were added to each well parallel to the treatments. The experiment plate was then immediately loaded into the TECAN SparkCyto for continuous incubation (37°C, 5% CO_2_) and automated fluorescence imaging. Images covering the entire well area (with automated stitching in the instrument software) were acquired for each well in a fully automatized manner every 3 h using a 4× objective and the kinetic cycle and multicolor analysis options of the TECAN SparkControl software. Fluorescence microscopy images were acquired consecutively with 60 ms exposure for blue fluorescence (Hoechst 33342, excitation 381–400 nm, emission 414–450 nm), 150 ms for green fluorescence (ApoTracker Green, ex. 461–487 nm, em. 500–530 nm) and 200 ms for red fluorescence (propidium iodide, ex. 543–566 nm, emission 580–611 nm). Image analysis was optimized with TECAN ImageAnalyzer software and key settings were transferred to TECAN SparkControl for real‐time image analysis. Individual cells were recognized based on nuclear staining (Hoechst 33342), and two secondary masks (for green and red fluorescence, respectively) were used to measure the green and red fluorescence signals associated with each cell. The secondary masks were defined based on the nuclear mask (using Voronoi mask) with a radius of 14 μm and an intensity threshold of 0.02 and 0.01 RFU (relative fluorescence units) for green and red fluorescence, respectively. Nuclei counts and fractions of cells in each bin (i.e., blue^+^/green^+^, blue^+^/red^+^ or blue^+^/green^+^/red^+^) at each time‐point were exported in Microsoft Excel format and are provided in Dataset EV6.

## Author contributions

**Karin Ortmayr:** Conceptualization; resources; data curation; software; formal analysis; funding acquisition; validation; investigation; visualization; methodology; writing—original draft; project administration; writing—review and editing. **Mattia Zampieri:** Conceptualization; resources; supervision; funding acquisition; methodology; writing—original draft; project administration; writing—review and editing.

In addition to the CRediT author contributions listed above, the contributions in detail are:

KO conceived and designed the project with advice from MZ. KO and MZ developed the experimental‐computational methodology. KO carried out all experiments and analyzed the data. KO and MZ wrote the manuscript.

## Disclosure statement and competing interests

The authors declare that they have no conflict of interest.

## Supporting information



Appendix

Dataset EV1

Dataset EV2

Dataset EV3

Dataset EV4

Dataset EV5

Dataset EV6

## Data Availability

Generated data are included in the Appendix and EV Datasets. Raw MS ion intensity data can be downloaded from https://www.ebi.ac.uk/biostudies with the accession number S‐BSST894.
