# Supplementary material for: Sorting‐free metabolic profiling uncovers the vulnerability of fatty acid β‐oxidation in in vitro quiescence models
Source: Mol Syst Biol. 2022 Sep 12;18(9):e10716. doi: 10.15252/msb.202110716 (PMC9465820; doi:10.15252/msb.202110716)
Supplement: Supplementary file 1 — Appendix [file MSB-18-e10716-s002.pdf]

Sorting-free metabolic profiling uncovers the vulnerability of fatty acid β-oxidation in quiescence

Karin Ortmayr[1,2] and Mattia Zampieri[1,*]

Affiliations:

[1] Institute of Molecular Systems Biology, ETH Zürich, Switzerland

[2] Division of Pharmacognosy, Department of Pharmaceutical Sciences, Faculty of Life Sciences, University of Vienna, Austria

* to whom correspondence should be addressed: zampieri@imsb.biol.ethz.ch

Appendix

Table of Contents

Appendix Figure S1. Characterization of quiescence- and growth phenotypes by flow cytometry and live-cell imaging

Appendix Figure S2. Impact of trypsin-mediated cell detachment on cellular metabolome profiles.

Appendix Figure S3. Verification of linearity of MS ion intensity measurements in different cell extracts.

Appendix Figure S4. Validation of the deconvolution procedure and identification of common patterns of metabolic changes.

Appendix Figure S5. Analysis of stimulus-dependent metabolic adaptations to quiescence (G0).

Appendix Figure S6. Impact of trimetazidine on growth phenotypes and viability in serum starvation and glutamine limitation.

Appendix Figure S7. Effect of trimetazidine on citrate accumulation, and impact of metabolite supplementation on trimetazidine-induced toxicity in serum starvation.

Appendix Figure S8. Impact of trimetazidine on cell metabolism and apoptosis induction in full medium vs. serum starvation.

Appendix Figure S9. Impact of trimetazidine on the return to proliferation after quiescence exit.

References

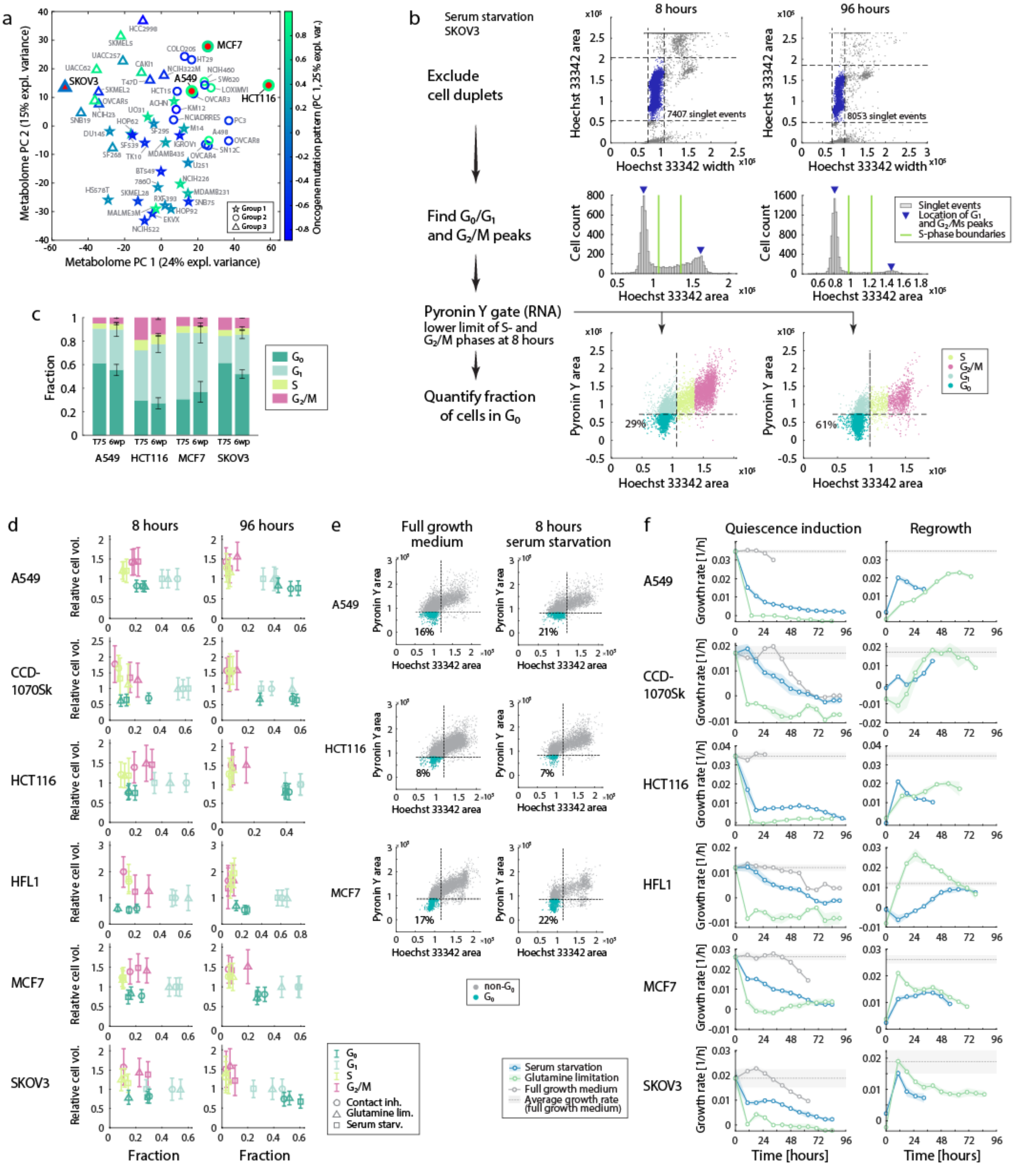

Appendix Figure S1. (a) First two principal components of previously published intracellular metabolome profiles of 54 human cancer cell lines (Ortmayr et al., 2019) (NCI-60 panel) identifying 3 major groups (circle, triangle and pentagram symbols). We additionally performed principal component analysis on the mutation state in 18 known cancer-genes in the same cell lines (Ikediobi et al., 2006) to group cell lines with a similar mutation profile (in colors, principal component 1, explaining 25% of the variance). The four cell lines used in this study are highlighted with red-filled symbols. **(b)** Schematic representation of the data processing workflow for the quantification of G_0 fractions based on single-cell DNA- and RNA-contents measured via Hoechst 33342- (DNA) and Pyronin Y (RNA) staining in a flow cytometry assay. First, cell clusters (e.g. cell duplets) that falsify the quantification of single-cell signal intensities were excluded by combining Hoechst 33342 area and width measurements, retaining only measurements from individual cells. In the second step, the distribution of Hoechst 33342 intensities serves for the identification of G_0/G_1- and G_2/M phase cell sub-populations (2n and 4n DNA contents, respectively). Cells with Hoechst 33342 intensities between G_0/G_1 and G_2/M phases were classified as S-phase cells. In the last step, the lower level of RNA contents (Pyronin Y intensity) of cells in S- and G_2/M phases is determined for each cell line in the control sample containing mostly proliferating cells (i.e. 8 hours after quiescence

induction), and is used in matching samples at 8 and 96 hours quiescence induction to quantify the fraction of cells in G_0. **(c)** Comparison of flow cytometry-based quantification of cells in G_0 in four cancer cell lines induced to enter quiescence by contact inhibition for 96 hours, in two different cultivation formats, i.e. 75 cm^2 cell culture flasks (T75) and 6-well cell culture dishes (6wp). Error bars reflect the standard deviation of fractions estimated from triplicate samples and flow cytometry measurements in 6-well plates. **(d)** Scatter plot showing the average relative cell volumes of cells in different cell cycle phases (normalized to G_1 phase) in relation to their frequency in the cell population (x-axis, fraction determined by flow cytometry as shown in panel b). Relative cell volumes in different cell cycle phases were estimated based on side-scatter area (SSC-A) measurements (Tzur et al., 2011) collected in parallel to Hoechst 33342 and pyronin Y measurements using flow cytometry. Results are shown for all six cell lines in separate sub-panels and the three quiescence stimuli in circle, triangle and square symbols. **(e)** Fraction of G_0 cells quantified using flow cytometry in three cell lines as described in panel b, either during growth in full growth medium or after 8 hours of serum starvation. Cell cultures exposed to serum-free conditions for only 8 hours (i.e. reference sample for metabolome profiling) exhibit similar fractions of G_0 cells as in full growth media. **(f)** Growth rates were estimated from cell confluence data (collected using time-lapse microscopy, Figure 1 in the main text) by linear regression of log-transformed confluence data at different time points using moving windows (24 hours width, 8 hours step size, see Methods section for a detailed description). Results are shown for quiescence induction- and regrowth phases in separate subpanels for each cell line. Results from serum-starved cultures are shown in blue, glutamine limitation in green. The gray line represents the average growth rate observed in a proliferating cell culture. Shaded areas indicate the standard deviation across three replicates.

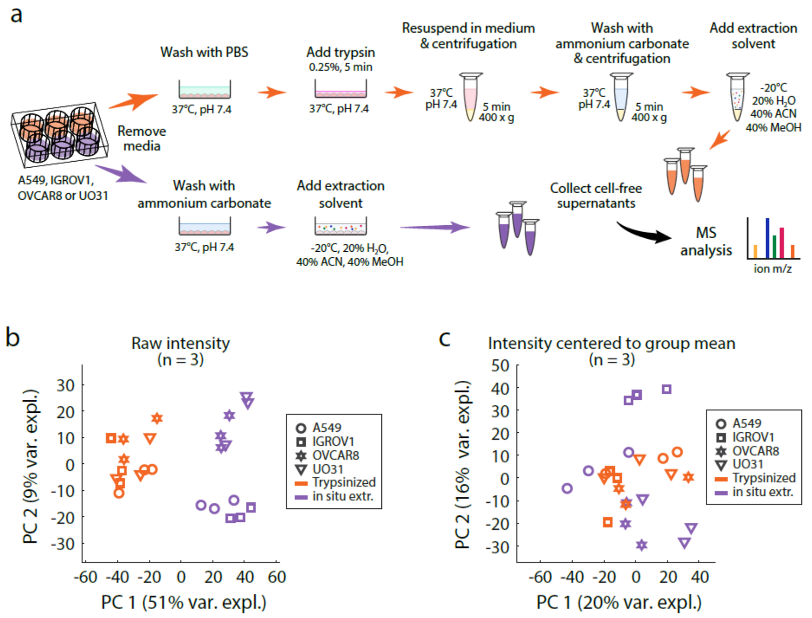

Appendix Figure S2. (a) Overview and comparison of sample processing steps for trypsin-mediated cell detachment and extraction vs. in situ extraction of adherent cells (Ortmayr et al., 2019) without prior cell detachment. Trypsin enzymatically degrades extracellular matrix components that mediate cell attachment to the surface of the culture dish. Once cells are detached, trypsin is inactivated by addition of serum-containing medium, and cells are harvested by centrifugation. Metabolites can be extracted after an additional washing step to remove residual media. In contrast, in situ extraction of adherent cells allows very fast processing, requiring only one washing step before cells are lysed while still attached to the culture dish. Shorter and less invasive sample processing minimizes the risk of metabolic artefacts caused by cellular stress responses to experimental conditions. **(b)** We generated cell extracts of four different cell lines (A549, IGROV1, OVCAR8 and UO31 cells) using two different extraction methods, i.e. extraction after enzymatic detachment using trypsin, or non-invasive in situ extraction (Ortmayr et al., 2019) without prior cell detachment, and compared the metabolite abundances measured using high-throughput metabolome profiling (Fuhrer et al., 2011). Identical biomass amounts of each cell line were used for the two extraction methods. Scatter plots report the major trends, i.e. principal components (PC) 1 and 2, distinguishing metabolic profiles of four different cell lines in cell samples generated following trypsin-mediated cell detachment ("Trypsinized", orange symbols) as compared to in situ extraction (purple symbols). Principal component analysis (PCA) was performed on raw intensity data without prior normalization (left plot), and additionally after centering the data for each extraction method to the mean within groups (right plot) to account for potential systematic differences in extracted biomass. While in situ-derived metabolite extracts revealed distinct metabolic profiles of the four cell lines, the profiles detected following trypsin-mediated detachment were overlapping masking characteristic metabolic differences between cell lines (Appendix Figure S2). These results indicate that the authentic metabolic differences between cell lines were obscured by cell detachment, washing and centrifugation steps and the relatively longer time until metabolism is quenched by addition of organic solvents.

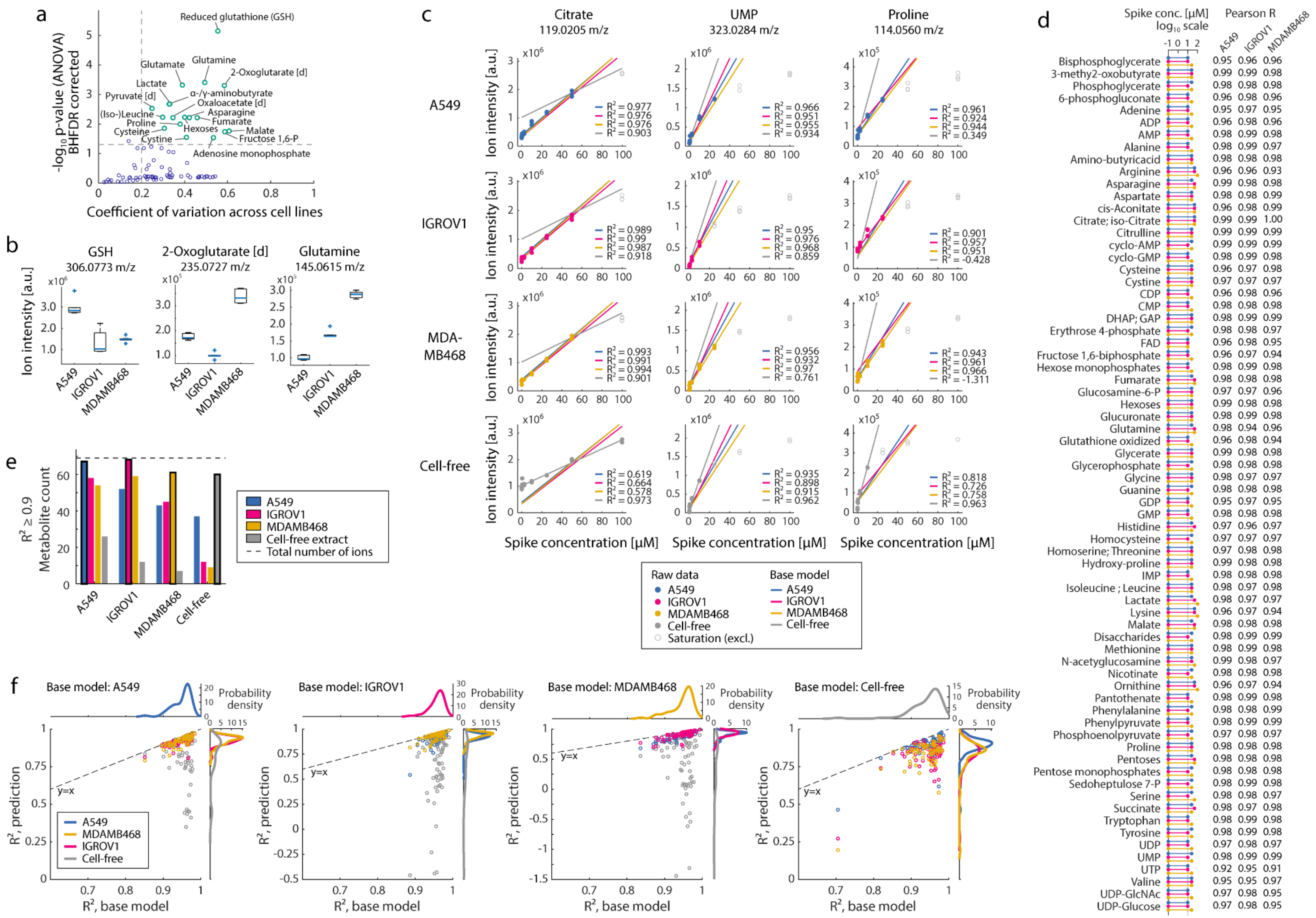

Appendix Figure S3. (a-b) Extracts of A549, IGROV1 and MDAMB468 cells represent different sample matrices, i.e. show different basal abundances of several metabolites typically present at high concentrations. One-way ANOVA uncovered significant differences across cell lines (y-axis, adjusted p-value < 0.05, Benjamini-Hochberg multiple testing correction)

for 22 metabolites with greater than 20% variance (x-axis) that were also contained in the spike mix. Matrix-associated ions included metabolites present in cells at high concentrations, like reduced glutathione and 2-oxoglutarate (detected as phenyl hydrazone derivative (Zimmermann et al., 2014)), glutamine and glutamate, for which boxplot distributions are shown in panel **b**. Box edges represent 25th and 75th percentiles, the horizontal blue bars indicate the median and blue plus signs represent outliers. **(c)** Evaluation of linearity in MS ion intensity measurements in a standard addition layout to rule out effects of different sample matrices (e.g. cell line extracts) on measurement linearity, and thereby on the deconvolution procedure. Increasing amounts of 105 metabolites were spiked into four different sample matrices (organized as rows), i.e. cell extracts of three different cell lines (A549, IGROV1 and MDAMB468), or cell-free extraction solvent. Example data curves are shown for endogenous metabolites citrate, uridine 5'-monophosphate (UMP) and proline. Intensity measurements at an x-axis value of 0 correspond to unspiked cell extract samples, and reflect the endogenous abundance of the metabolite in the cell extract. For each spiked and annotated metabolite (82 unique ions, 96 metabolites), linear regression analysis was used to fit a base model determining the slope and intercept of the linear relationship between metabolite concentration and ion abundance for each sample matrix separately. Ion intensity measurements above the saturation limit (open circle symbols), caused by ion suppression effects during ionization and ion transfer as well as MS detector capacity, were excluded. **(d)** The linear range for the relative quantification of 86 endogenous metabolites (70 unique ions) in cell extracts of three different cell lines, i.e. A549, IGROV1 and MDAMB468 (see also panel **c**) extended at least 2 orders of magnitude in concentration beyond basal metabolite concentrations in cell extract samples. The linear range was defined for each sample matrix (i.e. cell line) separately based on ion intensity measurements in cell extract samples spiked with increasing amounts of metabolites (0.1 – 100 µM final concentration), by determining the concentration range with the highest Pearson correlation (Pearson R) after excluding measurements close to the saturation limit. **(e-f)** The four different base models derived from fitting measured ion intensities of spiked increasing concentrations of 96 metabolites in the extracts from 3 different cell lines and cell free extract (panel **c)** were subsequently applied to each of the other sample matrices, and R^2 values were calculated to evaluate the goodness of fit (values in plot legends in panel **c**). This analysis quantifies whether the linear relationship between changes in metabolite concentration and ion intensity is independent of changes in sample matrix (i.e. cell lines, panel **a**). For more than 80% of spiked metabolites (panel **e**), we observed excellent reproducibility ($R^2 > 0.9$) of the base models fit on any of the three cell lines also in the other two cell line matrices, confirming that linearity is preserved across cell extract samples with similar extracted cell numbers. Scatter plots in panel **f** relate for each metabolite the R^2 value for the base model (x-axis) to the R^2 value when the same model was applied to ion intensity measurements in another sample matrix (y-axis). Sample matrices are encoded in data point colors. As expected, because of the radical difference in sample composition and hence complexity of the sample matrix in the cell-free sample (i.e. pure metabolite mixtures spiked into fresh extraction solvent), larger deviations are observed when applying the cell-free base model to cell line extracts and vice versa.

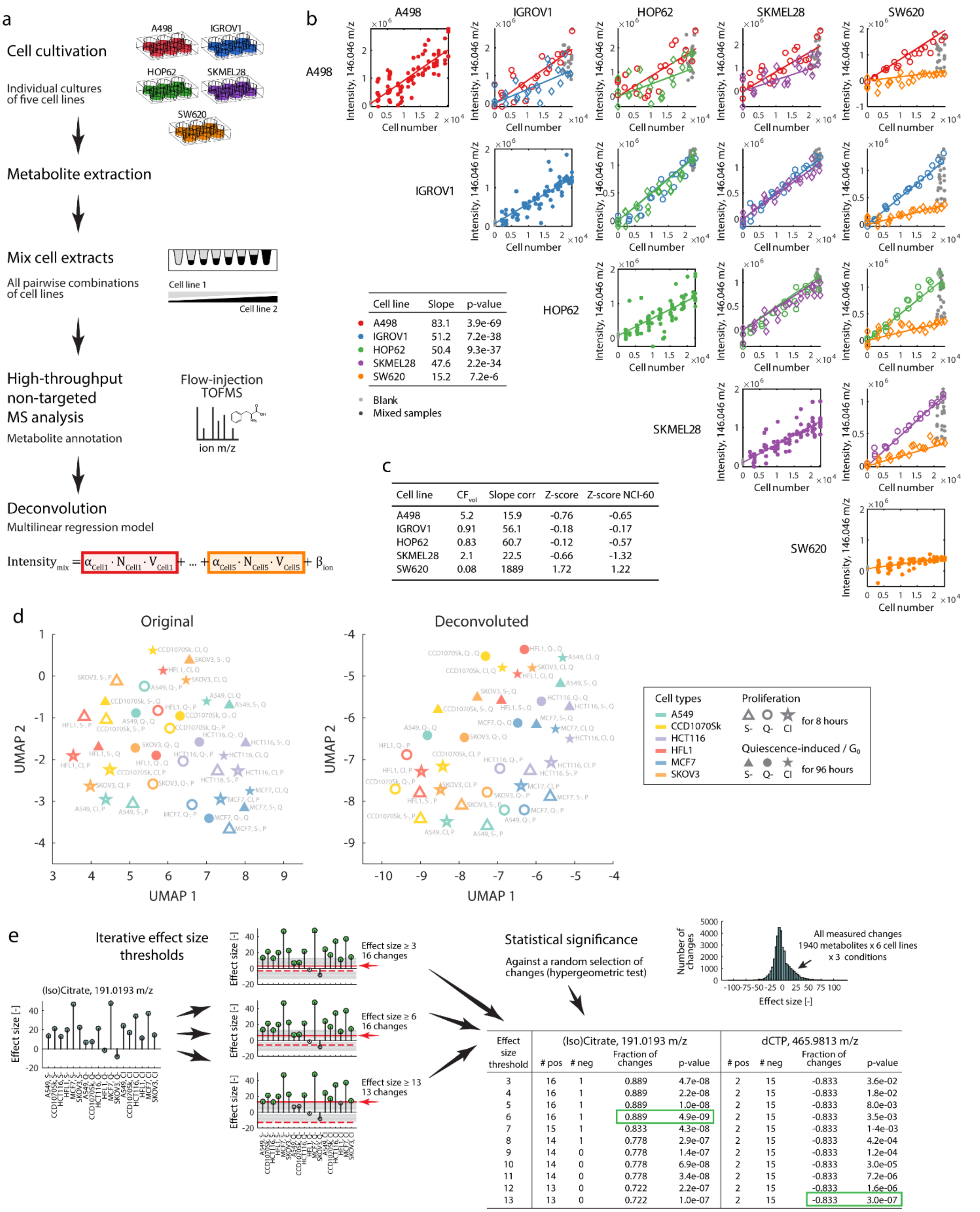

Cell line	Slope	p-value
A498	83.1	3.9e-69
IGROV1	51.2	7.2e-38
HOP62	50.4	9.3e-37
SKMEL28	47.6	2.2e-34
SW620	15.2	7.2e-6

Cell line	CF_{vol}	Slope corr	Z-score	Z-score NCI-60
A498	5.2	15.9	-0.76	-0.65
IGROV1	0.91	56.1	-0.18	-0.17
HOP62	0.83	60.7	-0.12	-0.57
SKMEL28	2.1	22.5	-0.66	-1.32
SW620	0.08	1889	1.72	1.22

Effect size threshold	(Iso)Citrate, 191.0193 m/z				dCTP, 465.9813 m/z			
	# pos	# neg	Fraction of changes	p-value	# pos	# neg	Fraction of changes	p-value
3	16	1	0.889	4.7e-08	2	15	-0.833	3.6e-02
4	16	1	0.889	2.2e-08	2	15	-0.833	1.8e-02
5	16	1	0.889	1.0e-08	2	15	-0.833	8.0e-03
6	16	1	0.889	4.9e-09	2	15	-0.833	3.5e-03
7	15	1	0.833	4.3e-08	2	15	-0.833	1-4e-03
8	14	1	0.778	2.9e-07	2	15	-0.833	4.2e-04
9	14	0	0.778	1.4e-07	2	15	-0.833	1.2e-04
10	14	0	0.778	6.9e-08	2	15	-0.833	3.0e-05
11	14	0	0.778	3.4e-08	2	15	-0.833	7.2e-06
12	13	0	0.722	2.2e-07	2	15	-0.833	1.6e-06
13	13	0	0.722	1.0e-07	2	15	-0.833	3.0e-07

Appendix Figure S4. (a) Schematic representation of the experimental setup used to validate the methodology (main text Figure 2a-b) to separate subpopulation metabolic profiles in heterogeneous cell cultures without physical separation. Metabolite extracts were collected from individual cultures of five different cell lines, and cell counts per sample were obtained using a Countess II cell counter (Invitrogen). Two cell lines each were mixed at eight different ratios to generate cell extract samples with a counter-gradient of the two cell lines, keeping the total cell number per sample constant. The procedure was repeated for all pairwise combinations of the five cell lines, and all samples were analyzed by FIA-TOFMS (Fuhrer et al., 2011). After putative annotation of metabolites against HMDBv4 (Wishart et al., 2018) and metabolites listed in Recon3D (Brunk et al., 2018), the multilinear regression scheme described in detail in the methods section was applied to separate the metabolite abundances of the individual cell lines from the mixed sample measurements. **(b)**

Detailed overview of the validation results for the deconvolution of metabolite abundances using the herein-presented regression-based approach (main text Figure 2a-b), modeling the total measured ion abundance in a mixed sample as a linear combination of the metabolite abundances in the two subpopulations. Results are shown for glutamate as a representative intracellular metabolite. The leftmost plot in each row shows the linear dependency of metabolite abundance on the number of cells obtained by applying the deconvolution. The coefficient values describing the change in intensity per change in cell number (i.e. slope) and its statistical significance are reported in the table. Each subplot in the upper right shows the linear combination of these basal dependencies to yield the total ion abundance measured in the mixed cell extract samples (dark grey data points). **(c)** Comparison of cell line metabolic profiles estimated using the regression-based approach (panels a-b) with relative metabolite abundances measured using a standard approach (Ortmayr et al., 2019). Glutamate is shown as a representative example (see also panel b). The relative metabolite abundances ("Slope" value in the table) were corrected for differences in cell volume (Slope corr) using a correction factor (CF_{vol}) obtained as described previously (Ortmayr et al., 2019). Z-score normalization was applied to the relative metabolite abundances for the five cell lines in both data sets individually prior to systematic comparison using correlation analysis (main text Figure 2c). **(d)** Comparison of metabolic profiles with and without deconvolution to separate metabolic signatures of cell subpopulations (i.e. G_0- vs. non-G_0), in six different cell lines and three different quiescence-inducing condition using uniform manifold approximation and projection (UMAP (McInnes et al., 2018)). Filled symbols indicate measurements at 96 hours, where populations are enriched for G_0 cells, while open symbols correspond to samples taken at 8 hours, containing mostly cells in G_1 phase (see Figure 1). To evaluate the effect of deconvolution, purified metabolite intensity profiles were calculated by subtracting the ion intensity contribution of G_0- and non-G_0 cells predicted by the deconvolution model from the total measured ion intensities (see Methods section). While before deconvolution, metabolic profiles are in part dominated by cell-type specific signatures, a major trend reflecting conserved metabolic traits of G_0 cells emerges in the data after separating the subpopulation metabolic signatures by deconvolution. **(e)** Schematic representation of the iterative thresholding approach used to identify common quiescence-associated patterns of changes in metabolite abundances (effect size) between G_0- and non-G_0 cells (main text Figure 2f). For each putatively annotated metabolite (here, citrate is shown as an example) different thresholds were set iteratively, each time counting the number of conditions (i.e. six cell lines and three quiescence stimuli) in which the effect size exceeded the threshold value. The counting was done separately for positive and negative effect sizes, and the sign with the larger number of changes was retained and reported as the fraction of changes exceeding the threshold out of all 18 conditions. At each threshold, the statistical significance (p-value) of the pattern was estimated using a hypergeometric testing layout to test the fraction of changes exceeding the threshold against the number of changes expected in a random sample of changes from the entire data set, given the frequency of different effect size signs and magnitudes. From the fractions and p-values collected at the different threshold, the minimum p-value and corresponding fraction (i.e. most significant common pattern) is selected for each metabolite (main text Figure 2f).

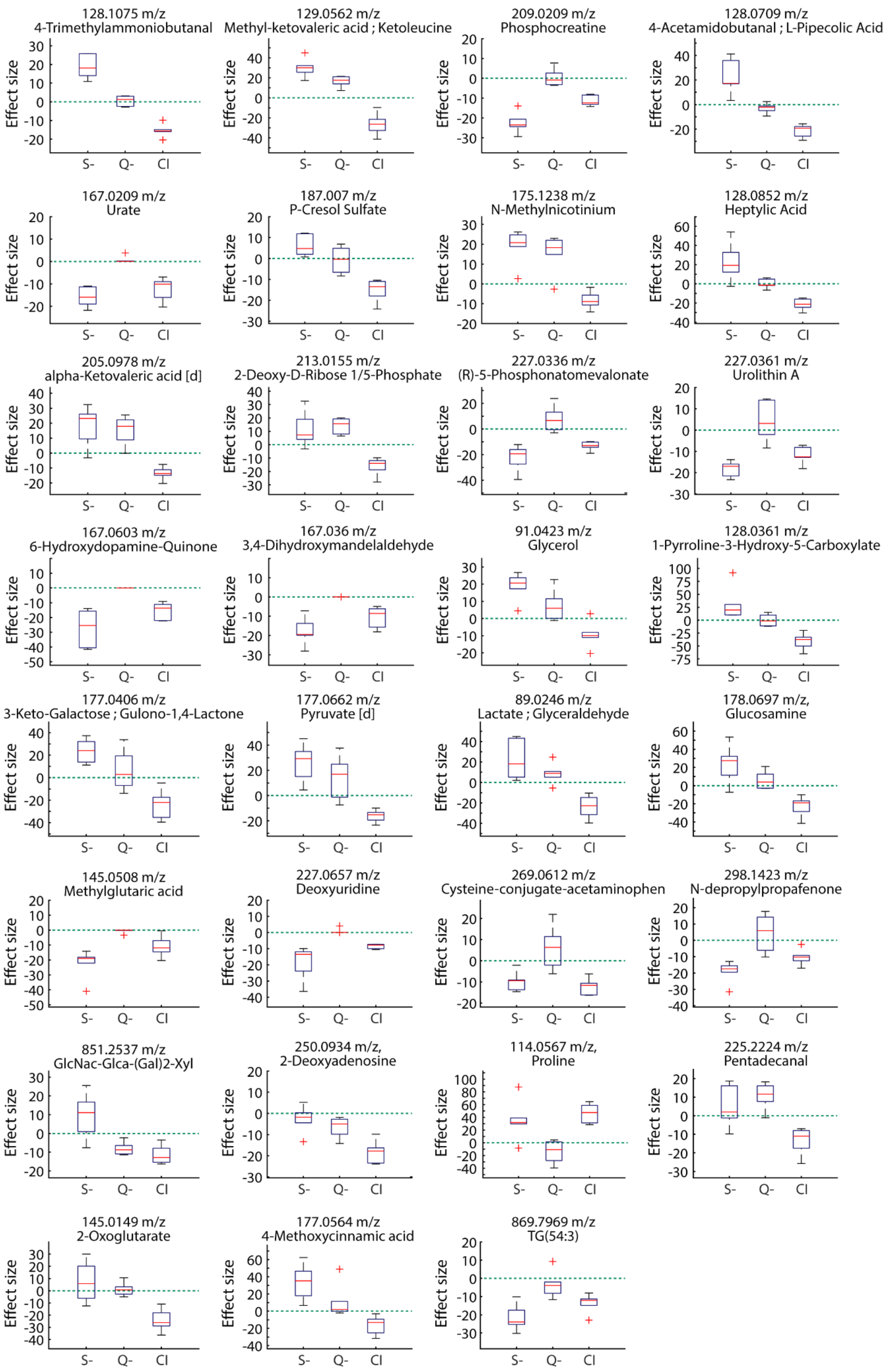

Appendix Figure S5. Analysis of stimulus-dependent metabolic adaptations to quiescence (G_0). Boxplots for 31 putatively annotated metabolites where changes in metabolite abundance between G_0- and non-G_0 cells (effect size, y-axis) in six cell lines were significantly dependent (1-way ANOVA p-value < 0.05, Benjamini-Hochberg multiple testing correction) on the G_0-inducing stimulus, i.e. serum starvation (S-), glutamine limitation (Q-) or contact inhibition (CI). Box edges represent 25th and 75th percentiles, horizontal red bars indicate the median and red plus signs represent outliers. The horizontal green line indicates an effect size of 0, i.e. no difference in abundance between G_0- and non-G_0 cells. The suffix [d] indicates α-keto acids detected as phenyl hydrazine derivatives (Zimmermann et al., 2014).

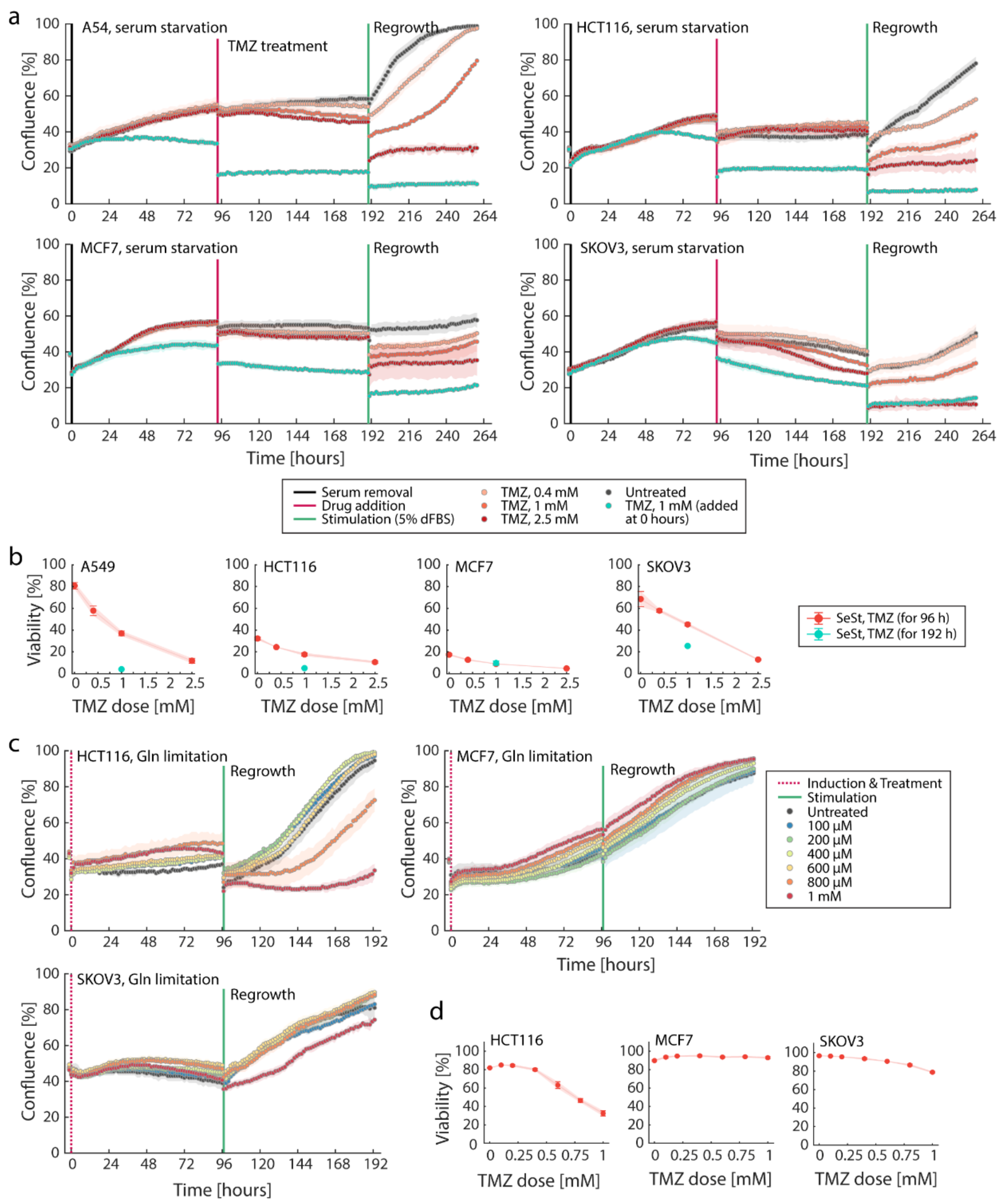

Appendix Figure S6. (a) Growth dynamics (cell confluence) upon trimetazidine (TMZ) treatment after the entry phase of quiescence. Four cancer cell lines (A549, HCT116, MCF7 and SKOV3) were first induced to enter quiescence by serum starvation in RPMI-1640 medium without drug addition. At 96 hours, the medium was removed (causing a slight decrease in confluence as detached and dead cells are removed) and replaced by fresh serum-free medium containing TMZ at final concentrations of 0.4, 1 or 2.5 mM (symbols in shades of red). In parallel, three replicate cultures of each cell line were treated with TMZ already when serum was removed (1 mM dose from 0 hours on, blue data points), and treatment was maintained for a total of 192 hours. At 192 hours, the medium was again removed in all cultures, and was replaced by full growth medium containing 5% dFBS without TMZ, to stimulate regrowth from quiescence. Cell confluence as a proxy of cell numbers was monitored using automated time-lapse microscopy in a plate reader (TECAN Spark 10M). **(b)** Viability at 192 hours as a function of TMZ concentration in the serum-starved cell cultures shown in panel a. Red color indicates that TMZ was added only after the initial induction phase (at 96 hours in panel a), while data points in blue correspond to cells continuously treated with TMZ for 192 hours. Viability was assessed using a fluorescence imaging assay in a TECAN Spark Cyto, following staining of all nuclei by the cell-permeable DNA stain Hoechst 33342, and dead cells by cell-impermeable propidium iodide (2 µg/mL each). **(c)** Growth phenotypic changes induced by trimetazidine (TMZ) treatment in A549 and HCT116 cells grown in glutamine-free RPMI-1640 medium (with 5% dialyzed FBS). Cell confluence as a proxy of cell numbers was monitored using automated time-lapse microscopy in a plate reader (TECAN Spark 10M). **(d)** Viability at 96 hours as a function of TMZ concentration in the cell cultures shown in panel c. Viability was assessed as described in panel b.

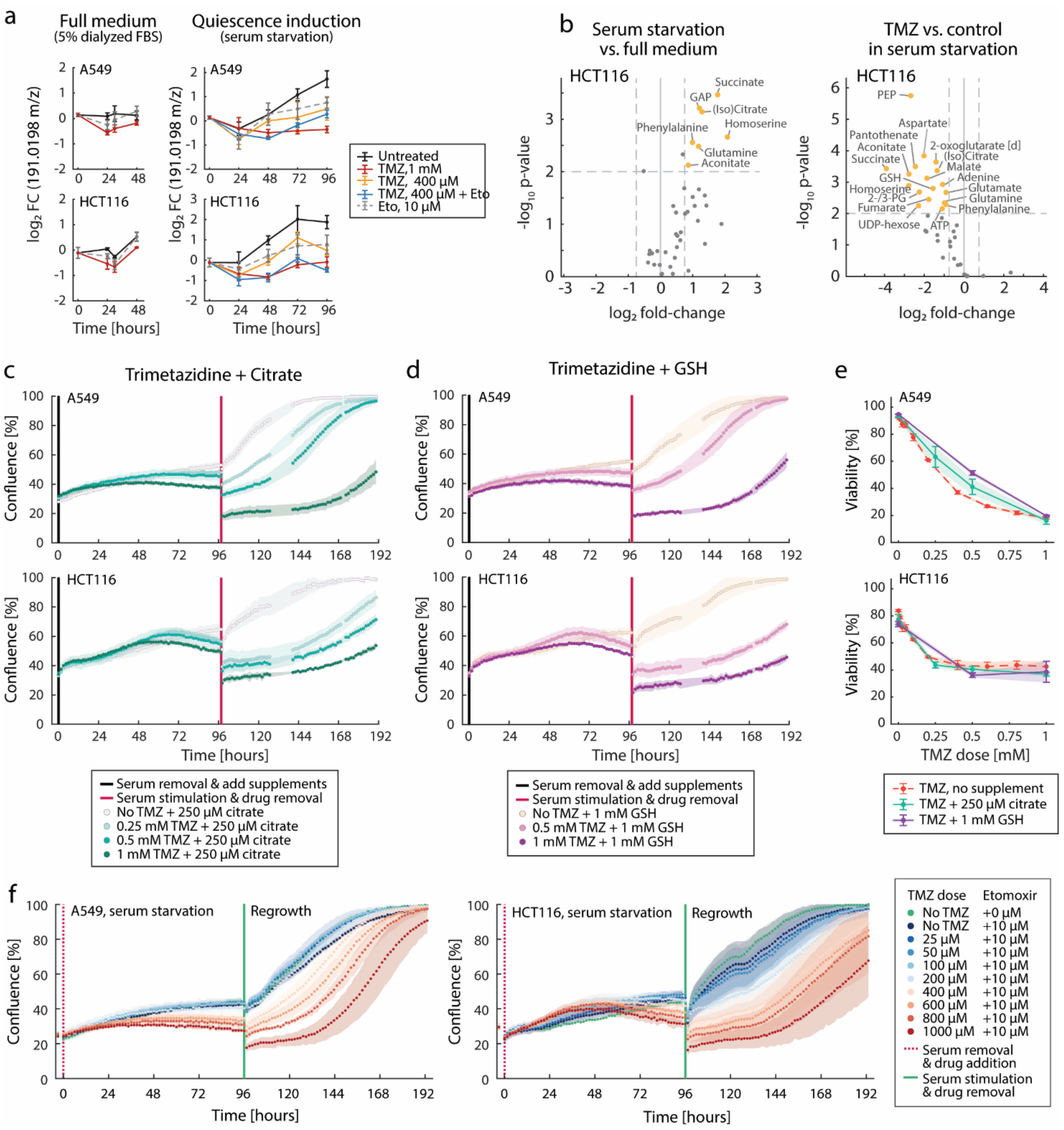

Appendix Figure S7. (a) Dynamic changes in the intracellular levels of citrate in A549 and HCT116 cells during rapid proliferation ("full medium") or induced to enter quiescence by serum starvation with or without trimetazidine (TMZ), alone or in combination with 10 µM etomoxir. Fold-changes were estimated relative to steady-state levels in full medium (Dubuis et al., 2018). **(b)** Volcano plots showing changes in intracellular metabolite abundances ($\log_2$ fold-change, x-axis) induced in HCT116 colon cancer cells by serum starvation (as compared to control cells in full medium, left panel) as well as additional trimetazidine treatment (right panel), measured by targeted LC-MS/MS (see Methods section for full detail). **(c-d)** Growth curves showing dynamic changes in cell confluence (y-axis) in A549 and HCT116 cells (upper and lower plot, respectively) during quiescence induction (serum starvation, 0-96 hours) in presence of TMZ with or without supplementation of citrate (panel **c**) or reduced glutathione (GSH, panel **d**). At 96 hours, media were removed and replaced by full growth medium (5% dFBS, without TMZ, citrate and GSH) to stimulate regrowth. Cell confluence as a proxy of cell numbers was monitored using automated time-lapse microscopy in a plate reader (TECAN Spark 10M). **(e)** Viability at 96 hours as a function of TMZ concentration in the serum-starved cell cultures shown in panels **c-d**. Viability was assessed using a fluorescence imaging assay in a TECAN Spark Cyto, following staining of all nuclei by the cell-permeable DNA stain Hoechst 33342, and dead cells by cell-impermeable propidium iodide. **(f)** A549 and HCT116 cancer cells induced to enter quiescence by serum starvation were treated with combinations of different concentrations of trimetazidine and etomoxir at a fixed dose of 10 µM. 96 hours after quiescence induction, cells were washed and supplied with fresh full growth media without drug. Cell confluence was continuously monitored as described in panel **c-d**.

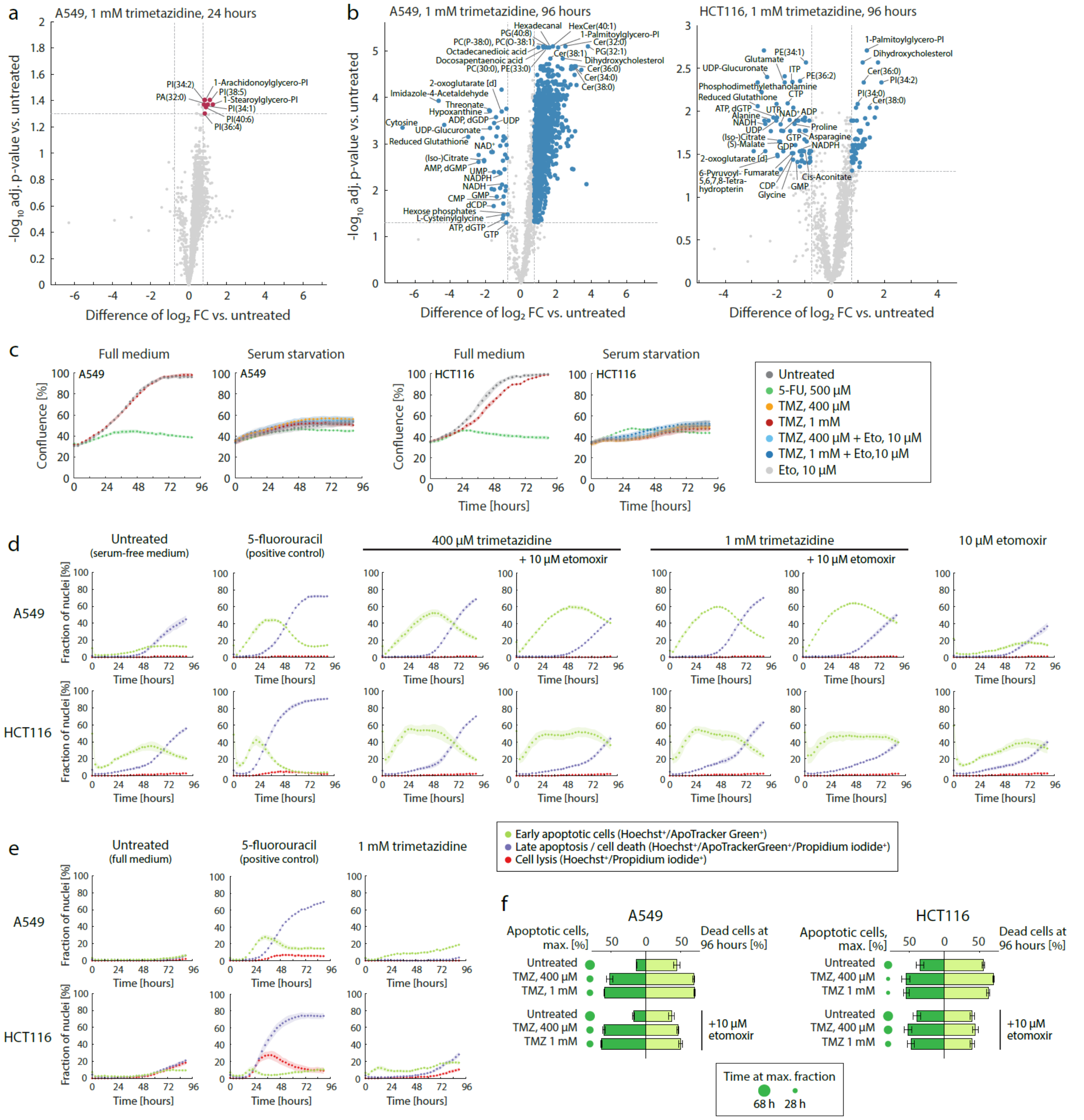

Appendix Figure S8. (a) Volcano plot showing metabolic changes induced by trimetazidine treatment (1 mM) in A549 cells induced to enter quiescence by serum starvation, at 24 hours after drug addition. Data shown represent the difference in log_2 fold-change of individual metabolites between trimetazidine treatment and serum starvation alone (x-axis), and the statistical significance (adjusted p-value, y-axis) of the difference (t-test, Benjamini-Hochberg correction). **(b)** Volcano plot showing characteristic metabolic changes induced by trimetazidine treatment (1 mM) in A549 and HCT116 cells under quiescence-inducing conditions (serum starvation), at 96 hours, i.e. before stimulation to regrow (main text Figure 3). Data are presented as described in panel **a**. **(c)** Measurements of cell confluence parallel to the dynamic monitoring of apoptosis (panels **d-e**), in A549 and HCT116 cells maintained either in full growth medium or serum starvation and treated with TMZ alone or in combination with 10 µM etomoxir. **(d-e)** Dynamic monitoring of apoptosis in live cell cultures in regular 3-hours intervals on a TECAN SparkCyto plate reader for automated fluorescence microscopy. Data are shown for A549 and HCT116 cell lines in quiescence-inducing conditions (panel **d**) or in full growth medium (panel **e**), treated with trimetazidine and/or etomoxir as indicated in the plot titles. 5-fluorouracil (500 µM) is used as a positive control for apoptosis induction. Early apoptotic cells are detected using ApoTracker Green (BioLegend) which binds to phosphatidylserine (PS) residues exposed on the outer cell surface. Staining by propidium iodide (PI) marks the loss of membrane integrity, allowing the identification of lysing (PI^+ only) or late apoptotic/necrotic cells ($ApoTracker^+/PI^+$). In addition, all cells were stained with low, non-toxic concentrations of Hoechst 33342 (see methods section) to allow automated cell segmentation and real-time cell classification based on a primary nuclear mask and

secondary measurements of ApoTracker Green and PI signals. Of note, such an assay provides a more realistic depiction of cell viability/death dynamics during the transition between rapid proliferation and quiescence than an end-point readout at 96 hours. Because the in situ fluorescence imaging approach is centered on adherent cells on the well bottom, floating dead cells are not captured at an end-point. A dynamic format of this assay allows capturing all stages from the viable state, to the induction and progression of apoptosis, until cell death. **(f)** Summary of the dynamics of apoptosis induction and cell death for treatments not shown in main text Figure 3l. For each treatment, the maximum fraction of apoptotic cells and time at which the maximum was reached was determined from the time-course data shown in panel **d**, and is reported alongside with the fraction of dead cells at 96 hours.

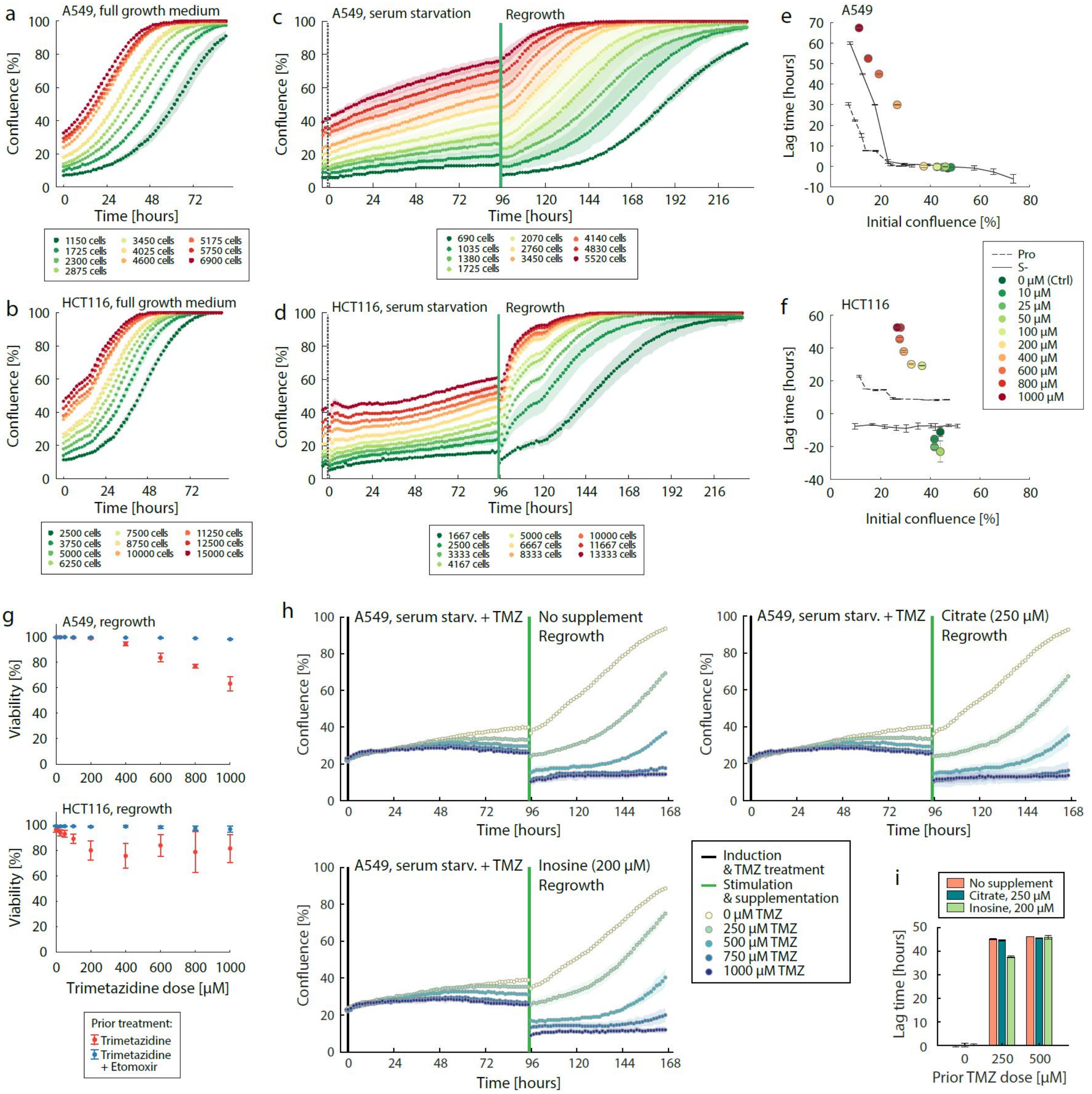

Appendix Figure S9. (a-b) A549 and HCT116 cell in full growth medium (RPMI-1640 with 5% dialyzed FBS and 2 mM glutamine) were seeded at different starting cell densities (legend indicates cells/well in a 96-well plate) and confluence was monitored using time-lapse microscopy (TECAN Spark 10M) until all cell cultures reached confluence. **(c-d)** A549 and HCT116 cell were seeded at different starting cell densities (legend indicates cells/well in a 96-well plate) and maintained in serum-free medium to induce quiescence (main text Figure 1). After 96 hours, all cultures were supplied with full growth medium to stimulate regrowth. Confluence was monitored using time-lapse microscopy (TECAN Spark 10M) until all cell cultures reached 100% confluence. **(e-f)** Lag times were estimated from cell confluence data shown in panels a-d and main Figure 3c, by first estimating the maximum growth rate (see Methods section for a detailed description), and subsequently finding the intersection between the tangent to the point with the highest growth rate and the initial confluence. **(g)** Viability (i.e. fraction of viable cells) of cells retained after restoring full growth media following serum starvation and treatment with trimetazidine alone (red data points) or in combination with 10 µM etomoxir (blue data points, see panel a). Viability was assessed using a fluorescence imaging assay following staining of all nuclei by the cell-permeable DNA stain Hoechst 33342, and dead cells by cell-impermeable propidium iodide. **(h)** Regrowth dynamics from serum starvation and trimetazidine (TMZ) treatment in A549 cells, with or without supplementation of citrate (250 µM) or inosine (200 µM). Serum starvation and TMZ treatment was maintained for 96 hours before stimulation by exchanging media for fresh full growth medium without TMZ but with either citrate or inosine. Growth was continuously monitored via confluence as described above. **(i)** Bar plot showing lag times estimated from the confluence data shown in panel g, describing growth dynamics during regrowth from serum starvation under trimetazidine (TMZ) treatment in presence or

absence of citrate or inosine. Lag times were only estimated for conditions where cells completed at least one doubling after serum stimulation and removal of TMZ from the medium.

References

Brunk, E., Sahoo, S., Zielinski, D.C., Altunkaya, A., Dräger, A., Mih, N., Gatto, F., Nilsson, A., Preciat Gonzalez, G.A., Aurich, M.K., Prlić, A., Sastry, A., Danielsdottir, A.D., Heinken, A., Noronha, A., Rose, P.W., Burley, S.K., Fleming, R.M.T., Nielsen, J., Thiele, I., Palsson, B.O., 2018. Recon3D enables a three-dimensional view of gene variation in human metabolism. Nature Biotechnology 36, 272–281. https://doi.org/10.1038/nbt.4072

Dubuis, S., Ortmayr, K., Zampieri, M., 2018. A framework for large-scale metabolome drug profiling links coenzyme A metabolism to the toxicity of anti-cancer drug dichloroacetate. Communications Biology 1, 101. https://doi.org/10.1038/s42003-018-0111-x

Fuhrer, T., Heer, D., Begemann, B., Zamboni, N., 2011. High-Throughput, Accurate Mass Metabolome Profiling of Cellular Extracts by Flow Injection–Time-of-Flight Mass Spectrometry. Anal. Chem. 83, 7074–7080. https://doi.org/10.1021/ac201267k

Ikediobi, O.N., Davies, H., Bignell, G., Edkins, S., Stevens, C., O’Meara, S., Santarius, T., Avis, T., Barthorpe, S., Brackenbury, L., Buck, G., Butler, A., Clements, J., Cole, J., Dicks, E., Forbes, S., Gray, K., Halliday, K., Harrison, R., Hills, K., Hinton, J., Hunter, C., Jenkinson, A., Jones, D., Kosmidou, V., Lugg, R., Menzies, A., Mironenko, T., Parker, A., Perry, J., Raine, K., Richardson, D., Shepherd, R., Small, A., Smith, R., Solomon, H., Stephens, P., Teague, J., Tofts, C., Varian, J., Webb, T., West, S., Widaa, S., Yates, A., Reinhold, W., Weinstein, J.N., Stratton, M.R., Futreal, P.A., Wooster, R., 2006. Mutation analysis of 24 known cancer genes in the NCI-60 cell line set. Mol Cancer Ther 5, 2606–2612. https://doi.org/10.1158/1535-7163.MCT-06-0433

McInnes, L., Healy, J., Melville, J., 2018. UMAP: Uniform Manifold Approximation and Projection for Dimension Reduction. arXiv:1802.03426 [cs, stat].

Ortmayr, K., Dubuis, S., Zampieri, M., 2019. Metabolic profiling of cancer cells reveals genome-wide crosstalk between transcriptional regulators and metabolism. Nature Communications 10, 1841. https://doi.org/10.1038/s41467-019-09695-9

Tzur, A., Moore, J.K., Jorgensen, P., Shapiro, H.M., Kirschner, M.W., 2011. Optimizing Optical Flow Cytometry for Cell Volume-Based Sorting and Analysis. PLOS ONE 6, e16053. https://doi.org/10.1371/journal.pone.0016053

Wishart, D.S., Feunang, Y.D., Marcu, A., Guo, A.C., Liang, K., Vázquez-Fresno, R., Sajed, T., Johnson, D., Li, C., Karu, N., Sayeeda, Z., Lo, E., Assempour, N., Berjanskii, M., Singhal, S., Arndt, D., Liang, Y., Badran, H., Grant, J., Serra-Cayuela, A., Liu, Y., Mandal, R., Neveu, V., Pon, A., Knox, C., Wilson, M., Manach, C., Scalbert, A., 2018. HMDB 4.0: the human metabolome database for 2018. Nucleic Acids Res 46, D608–D617. https://doi.org/10.1093/nar/gkx1089

Zimmermann, M., Sauer, U., Zamboni, N., 2014. Quantification and Mass Isotopomer Profiling of α-Keto Acids in Central Carbon Metabolism. Anal. Chem. 86, 3232–3237. https://doi.org/10.1021/ac500472c
