# Supplementary material for: Sorting‐free metabolic profiling uncovers the vulnerability of fatty acid β‐oxidation in in vitro quiescence models
Source: Mol Syst Biol. 2022 Sep 12;18(9):e10716. doi: 10.15252/msb.202110716 (PMC9465820; doi:10.15252/msb.202110716)
Supplement: Supplementary file 8 [file MSB-18-e10716-s008.pdf]

molecular systems biology

Sorting-free metabolic profiling uncovers the vulnerability of fatty acid β-oxidation in in vitro quiescence models

Karin Ortmayr and Mattia Zampieri
DOI: 10.15252/msb.202110716

Corresponding author(s): Mattia Zampieri (zampieri@imsb.biol.ethz.ch) , Karin Ortmayr (karin.ortmayr@univie.ac.at)

Review Timeline:

Submission Date:	27th Sep 21
Editorial Decision:	22nd Oct 21
Revision Received:	6th Jun 22
Editorial Decision:	21st Jul 22
Revision Received:	18th Aug 22
Accepted:	22nd Aug 22

Editor: Maria Polychronidou

Transaction Report:

(Note: With the exception of the correction of typographical or spelling errors that could be a source of ambiguity, letters and reports are not edited. Depending on transfer agreements, referee reports obtained elsewhere may or may not be included in this compilation. Referee reports are anonymous unless the Referee chooses to sign their reports.)

RE: MSB-2021-10716, Sorting-free metabolic profiling uncovers the essential homeostatic role of fatty acid β-oxidation in quiescence

Thank you again for submitting your work to Molecular Systems Biology. We have now heard back from the three referees who agreed to evaluate your study. Overall, the reviewers acknowledge that the presented approach and findings seem interesting. They raise however a series of concerns, which we would ask you to address in a major revision.

Without repeating all the points listed below, some of the more fundamental issues are the following:

- As reviewer #1 details in their comments, additional experiments are needed to more conclusively demonstrate that fatty acid β-oxidation is indeed required for the transition to proliferation and to examine if other metabolites and/or metabolic-related processes are also important.

- The reviewers mention that ideally the applicability of the method to a heterogeneous population should be demonstrated (even if only by in vitro experiments are reviewer #1 recommends).

- The comment above is also related to remarks by reviewers #2 and #3 who mention that the potential limitations of the approach e.g. when analysing complex tissues should be discussed.

- Reviewer #3 has raised some technical concerns, which need to be addressed and/or discussed.

- As reviewers #2 and #3 mention, the study would benefit from comparisons of the presented method to alternative approaches.

All issues raised by the reviewers would need to be satisfactorily addressed. Please contact me in case you would like to discuss in further detail any of the issues raised, I would be happy to schedule a video call.

Reviewer #1:

This manuscript describes a new method to analyze metabolic profiles in cell subpopulations without the need for physical separation. This method is also validated by profiling the metabolic features of quiescent cells. The authors find a key role of fatty acid degradation in the ability of cells to maintain cellular homeostasis during quiescence and show that this feature - which is independent of quiescence stimulus - is shared among different cell types. Furthermore, they found that inhibiting fatty acid β-oxidation impair their ability to restart proliferation.
In general, the manuscript is easy to read. The introduction is appropriate and the story line of the results is well structured. Additional experiments are needed to further prove whether fatty acid β-oxidation is indeed key to restart proliferation or, instead, other metabolites (e.g. citrate) and/or metabolic-related processes (e.g. mitochondria stability) are also crucial for this transition.

Major points:

- The major limitation of the study is that the mechanism of why quiescent cells need FAO is missing.
- Etomoxir is also preventing FAO. Thus, how do the authors expect to rescue trimetazidine-induced toxicity using etomoxir if FAO is important for quiescent cells? It would be important to test additional rescues that could restore FAO or its product (e.g acetate as an alternative source for acetyl-CoA production, or citrate).
- The authors state that the results suggest (345-346) while fatty acid degradation is dispensable for rapid proliferation, it becomes essential during quiescence-induced growth arrest and rapid growth resumption. Besides FOA, also the accumulation of citrate is lost upon trimetazidine treatment, thus the authors would need to exclude that the citrate accumulation is key to their phenotype.
- The authors mention in the discussion (447-448): Together with conventional anti-cancer agents, combination therapies (e.g. with trimetazidine) could simultaneously target highly proliferating and dormant cancer cells, and thereby reduce the risk of cancer recurrence. It would be more relevant if the authors could test this in an in vitro approach, e.g. mimicking tumor heterogeneity by combining proliferating and quiescent cells and treating with combination therapies as suggested. Additionally, since the authors do not provide any in vivo evidence for their mechanism suggesting trimetazidine as a treatment is not justified.
- Could this new methodology be applied to characterize differential metabolic profiles on a heterogeneous population? It would be interesting to validate it in vitro by combining different populations that one can find within a tumor, or in the tumor microenvironment (e.g, CAF and cancer cells...)
- The authors do not discuss current approaches to perform metabolic profiling of quiescent cells. Adding a simplified experiment comparing the new method with one of those and highlight potential advantages would make this approach and hence this manuscript more powerful.

Minor comments:

- While the described method and in vitro findings are very compelling, in vivo evidence for the proposed mechanism is missing. The authors at least need to discuss this and point out this limitation in their study. They should also rephrase the text to avoid therapeutic conclusions or conclusions about cancer dormancy
- The authors should avoid the term dormancy because they do not provide any data on true dormancy in cancer cells
- Figure 3f-g or lines 264-266: A549 and HCT116 cancer cells under serum starvation conditions, trimetazidine treatment caused drastic dynamic changes in cell confluence and induced cell death in a dose-dependent manner. Is it possible that those changes are related to serum (lipids) starvation rather than quiescence induction?
- Figure 3m seems to be essential to explain the hypothesis about fatty acid degradation playing a role in preparing cells for reversing quiescence but it is poorly explained in the text. It could be better explained since it is a complex figure showing an unconventional output.
- The authors show that the main differences in metabolic features are mostly due to the cell state rather than cell type or quiescence stimulus. Are there also any metabolic adaptations that differ when comparing environmental stimuli?

Reviewer #2:

In this manuscript, the authors describe the metabolism of quiescence by studying multiple different cancer and normal cell lines in proliferating and quiescent states. They use a mixing approach followed by linear regression to determine metabolites that are different when a variety of cell lines are induced into quiescence by one of multiple model systems. They discovered that whether the cells were quiescent or proliferating was the strongest determinant of the metabolic profile. The metabolites elevated in quiescent compared with proliferating cells included metabolites associated with fatty acid degradation. Based on these findings, the authors hypothesized that fatty acid degradation is selectively important for quiescent cells. They found that fatty acid degradation is important for viability of the quiescent cells and their ability to enter the cell cycle from quiescence. They propose that this is a fundamental property of quiescent cells that can be exploited for cancer therapy.

The study is a systematic approach to understanding the consistent changes in metabolism when different cell types are induced into quiescence by different signals. The finding of consistencies is interesting and will be informative for others interested in the quiescent state.

Comments

The authors report a novel sorting-free way to measure metabolite levels in quiescent cells.

There is a real problem and important problem for understanding the metabolism of quiescent cells in vivo. We want to know the metabolism of the stem cells in a mixed population taken straight from a tissue. If we sort for the small number of cells that are quiescent, we perturb the system and its metabolism during the sort and can't trust our data. If we do metabolomics on the whole tissue, we don't know what the contribution was of the quiescent cells.

The authors seem to be claiming that they solved this. But they are working with a system with cultured cells that they can transition to a quiescent condition. They can compare the proliferating and quiescent cells. The linear mixing that they perform may provide some improvement, but fundamentally, they could compare the cells cultured in conditions that generate mostly proliferating or mostly quiescent cells. This approach doesn't solve the problem that scientists trying to study complex tissues have, which is that they don't have a pure population of quiescent cells.

The authors should be careful to cite previous studies that have also investigated fatty acid oxidation in cells in quiescent states. As one example, the review by Shyh-Chang and Ng in Genes and Development in 2017 provides information on previous findings about the role of fatty acid oxidation in stem cells.

Fig 3l shows the extent of apoptosis and death with TMZ treatment. There are no statistical comparisons shown. There is more death in the treated quiescent cells than the treated full medium cells, but there is also more death and apoptosis in the untreated quiescent than untreated full medium cells. TMZ treatment may have significantly increased apoptosis in the full medium conditions as well as the quiescence conditions.

Figure 3M is a critical figure because the authors argue that it demonstrates that fatty acid oxidation not only causes death of quiescent cells but also a failure to re-enter the cell cycle. Fig 3b and 3c also show a delay in regrowth but it's not clear if that's a failure to proliferate or cell death. In Fig 3M, the authors compare lag times with and without trimetazidine treatment. They do this by monitoring growth at the maximum timepoint and extrapolating back, and comparing to untreated cells.
There are better ways to monitor proliferation at a specific timepoint than doing a backwards interpolation from a later timepoint. The authors could perform Ki-67 or BrdU labeling to determine the fraction of cells dividing.
Further, the data for A549 and HCT116 cells seem to be telling different stories. For A549 cells, it seems that for doses over 400 microM, there is a similar lag with treatment versus without, while for HCT116, the data seem to show a bigger lag with higher dose. However, these data are somewhat complicated to interpret because the experiment seems to have been performed differently for the two cell lines since for HCT116 but not A549, all of the data have the same expected lag. Can the authors please explain?

Reviewer #3:

Summary
Ortmayr and Zampieri show that a panel of four cancer cell lines and two fibroblast cell lines have similar growth dynamics upon induction of quiescence through three different stimuli: glutamine deprivation, serum starvation, and contact inhibition. They utilize a multiple term linear model to deconvolute cell population contributions to a mixed cell metabolome, estimating the relative intracellular abundances of metabolites in proliferating versus quiescent cells. Increased TCA cycle intermediates that are downstream of fatty acid oxidation (FAO) along with other lipid metabolism-associated metabolites in quiescent cells suggested modulation of FAO during quiescence. To test the dependence of quiescence on FAO, the authors inhibited beta oxidation with trimetazidine (TMZ). TMZ had an inhibitory effect on cell regrowth after quiescence release, and reduced cell

viability during quiescence induction. Further, signature metabolite abundance changes associated with quiescent cells were blocked by TMZ, and quiescent cultures displayed higher apoptosis.

Overall, this is an interesting manuscript that focuses on an important problem in metabolism: how do we deconvolute the metabolic contributions from different cell types when they are in mixed populations? In this reviewer's perspective, the work represents an interesting step forward; however, it is absolutely critical that the authors clearly highlight the limitations of their approach if they are to publish, which are not trivial as described below.

General remarks
This study confirms other published results that have shown the importance of FAO in endothelial cells, fibroblasts, neural stem cells, leukemia, and melanoma through its use of four additional cancer cell lines (PMID: 31375515, 28854364, 27374788, 20038799, 27049668, 21049082, 30146488). Other studies have suggested redox toxicity after FAO inhibition, which this study did not investigate. Thus, the major and primary advance of this study is technical: in designing a deconvolution method for mixed-cell metabolomics.
I have three significant concerns about the technical approach.
First, the authors assume that the total abundance of a metabolite is linearly dependent on the number of cells extracted. This is not necessarily true. The signal of a metabolite in mass spectrometry is not linear with respect to its concentration. Thus, a two-fold change in cell number could correspond to only a small change in MS signal. I am not sure how their model can address this intrinsic and non-predictable lack of linearity in MS data. Additionally, the signal of a metabolite is dependent upon its matrix. A metabolite standard spiked into one cell extract at a given concentration will give a different signal when it is spiked into another cell extract at the same concentration due to differences in matrix effects. Mixing cell types changes the matrix. The signals measured in pure cell types cannot be quantitatively compared to signals measured from mixed cell types (especially when using FIA). This seems like a serious complication to the approach described.
The second concern is that this approach will only work for a limited number of applications where cells can be purified and measured individually to generate a reference curve. Such analyses will not be possible for tissues. Thus, the method is really limited to a specific set of in vitro studies.
The third concern is that the approach assumes that intracellular metabolite levels will not change as a function of cellular environment. In other words, the authors assume that one million pure cells will produce the same amount of glutamate as one million of the same cells mixed with other cell types. However, this might not be true. For example, it is likely that the amount of a given metabolite in a cell will change depending on the density of other sub-populations in which it is cocultured. This is because cells reprogram their metabolism when they are cocultured with different cell types.

Recommendations
1. The most important revision that I urge the authors to consider is discussing the technical limitations above. They may not all be solvable, but the authors should at least discuss them in detail so that they do not mislead readers. For example, the title should highlight that this is an in vitro technique only. Perhaps "An in vitro sorting-free profiling technique", or something similar. The abstract should point out that the technique is not compatible with tissues. Each of the limitation points above should be described in detail in the discussion. Conclusions should be tempered based on these points. Readers should be cautioned of the assumptions being made and when they might break down (eg, maybe low abundance metabolites are more susceptible to non-linearity issues).
2. While the authors did assess a handful of cell types and observe similar patterns, drawing general conclusions about quiescence induction and release dynamics is risky. Such a claim would require experiments with many more non-fibroblast and non-cancer cell types. Even when generalizing about cancers from different tissues, several cell lines from each tissue should be utilized before generalizations are made. I recommend removing the claim that the observations are broadly applicable to all cell types rather than trying to prove this, which would require substantial experimental effort.
3. Quiescence release growth dynamics and apoptosis induction in quiescent cells by TMZ should be re-measured after isolation of quiescent cells via flow sorting to confirm that the dynamics are not due to a large population of non-quiescent cells continuing to proliferate and that the observed apoptosis is indeed in sensitized quiescent cells and not proliferating cells
4. The authors validate their method by comparing computationally derived results to known mixtures of independently grown cultures; however, since the computational method is based on dilution of known pure cell population metabolite extracts, this validation method is circular. The authors do not benchmark their method against other computational methods for deconvolution, so it is unclear how significant this advance is compared to previous methods (eg, 10.1109/BIBM.2016.7822519). They may wish to compare their method to the previously published one if they find that it is relevant to their system.
5. To validate their computational model, the authors may want to test a known metabolic change in a mixed cell population (eg, delivering an inhibitor to which one cell line is sensitive and the other is resistant with a previously published metabolic phenotype, or utilizing an inducible-oncogene cell line in which oncogene induction has metabolic effects combined with a cell type that has no reaction to the induction stimulus). Without such validation using previously published phenotypes or methods, it is difficult to truly accept the results produced by the computational model.
6. The authors write in the methods section that metabolites were only putatively annotated. This is particularly problematic in FIA where there are no retention times to compare to. The identity of metabolites should be confirmed with fragmentation data.

Minor points
1. What is the authors' explanation for the greater proportion of proliferating cells in CCD1070Sk cultures exposed to glutamine deprivation over other stimuli (Fig 1C) but the significantly lower (even reduced) confluence over time (Fig 1d)?

2. The authors should depict how cell number estimates are acquired in their graphical representation of the data acquisition workflow (Fig 2A).
3. The authors should illustrate the complete Intensity equation, including volume parameters (even though these were taken to be equal for all cell types) to accurately represent the model's inputs to the reader.
4. The authors may want to clarify the explanation for Fig 2C as this reviewer finds it currently difficult to follow. For example, what is the estimated density of correlations and how was it acquired? Is this a histogram?
5. Effect size or fold change information should be depicted in Fig 2F for the reader to be able to judge the biological significance of metabolite abundance changes outside of their statistical significance and association.
6. How do the authors account for the outliers in Fig 2G? Were biological and technical replicates measured? If not already, they should be.
7. Why is a continuous plot not shown for the data in Fig 3d and Fig 3e? It currently appears as if the data do not line up from one plot to another.
8. The Figure 3 legend has incorrect lettering progression (letter j is repeated where letter i should be).
9. Why did the authors not measure metabolite abundance at 96 hours post-TMZ when a larger phenotypic difference is observed?
10. The explanation for the scatter plot in Fig 3K should be clearer. What are the depicted fold changes relative to? Are they proliferating vs quiescent cells?
11. I also recommend that the authors re-work the manuscript title to reflect that their method is used to validate the importance of FAO to quiescence, which has been published before (eg, perhaps something like "An in vitro sorting-free profiling technique confirms the importance of...".
12. Would the authors please confirm with literature citations that all cancer cell lines used are contact-inhibited? If some are not, do they display differing levels of quiescence induction?
13. Would the authors please clarify the methods section about how the mass spectrometry was run? Currently it suggests that a liquid chromatography instrument was used despite the method citing flow-infection TOFMS.

Response to reviewer comments

Reviewer #1:

This manuscript describes a new method to analyze metabolic profiles in cell subpopulations without the need for physical separation. This method is also validated by profiling the metabolic features of quiescent cells. The authors find a key role of fatty acid degradation in the ability of cells to maintain cellular homeostasis during quiescence and show that this feature - which is independent of quiescence stimulus - is shared among different cell types. Furthermore, they found that inhibiting fatty acid β-oxidation impair their ability to restart proliferation.
In general, the manuscript is easy to read. The introduction is appropriate and the story line of the results is well structured. Additional experiments are needed to further prove whether fatty acid β-oxidation is indeed key to restart proliferation or, instead, other metabolites (e.g. citrate) and/or metabolic-related processes (e.g. mitochondria stability) are also crucial for this transition.

Major points:
- The major limitation of the study is that the mechanism of why quiescent cells need FAO is missing.

>> We have clarified the conclusions in the main text and added new experimental data. Our findings suggest that FAO provides a key mechanism for the clearance of fatty acid species and maintaining cellular homeostasis. This role extends the previously reported involvement of FAO in energy generation and redox homeostasis (Ito *et al*, 2012; Knobloch *et al*, 2017; Kalucka *et al*, 2018). We performed new experiments to show that neither providing the FAO product citrate (for energy generation as well as the regeneration of redox cofactors like NADPH) nor reduced glutathione (GSH) is sufficient to rescue cells from the toxicity induced by FAO inhibitor trimetazidine (TMZ). Similarly, supplementing citrate was not sufficient to facilitate the return to proliferation after TMZ treatment. Altogether, these results suggest that while FAO can be crucial for energy generation (Ito *et al*, 2012; Knobloch *et al*, 2017) and/or regeneration of redox cofactors (Kalucka *et al*, 2018) in long-term quiescent cells, during the initial transition to quiescence FAO plays a key role in avoiding the accumulation of intermediates of fatty acid degradation in mitochondria.

- Etomoxir is also preventing FAO. Thus, how do the authors expect to rescue trimetazidine-induced toxicity using etomoxir if FAO is important for quiescent cells? It would be important to test additional rescues that could restore FAO or its product (e.g acetate as an alternative source for acetyl-CoA production, or citrate).

>> We thank the reviewer for raising this point which we now clarify. Etomoxir was developed as an inhibitor of fatty acyl transport into mitochondria (i.e. inhibiting CPT-1), and can reduce fatty acid-driven respiration by approximately 50% at the moderate doses used in this study (10 µM) (Divakaruni et al, 2018). At higher dosages, other indirect effects can dominate (Divakaruni et al, 2018). In combination with trimetazidine treatment, etomoxir, by limiting the entry of FAO substrates into mitochondria, can reduce the accumulation of mitochondria-damaging intermediates and/or ROS. Most importantly, etomoxir does not inhibit peroxisomal fatty acid degradation pathways, or fatty acid secretion into the extracellular space, two alternative ways by which cells can compensate for a reduced mitochondrial FAO and avoid accumulation of toxic intermediates.

- The authors state that the results suggest (345-346) while fatty acid degradation is dispensable for rapid proliferation, it becomes essential during quiescence-induced growth arrest and rapid growth resumption. Besides FOA, also the accumulation of citrate is lost upon trimetazidine treatment, thus the authors would need to exclude that the citrate accumulation is key to their phenotype.

>> This is a crucial aspect which we now clarify. We thank the reviewer for the suggestion. We have added new experimental evidence to show that increased availability of citrate is not sufficient to rescue

quiescence-induced cells from trimetazidine-induced toxicity, and supplementing citrate is similarly not sufficient to facilitate rapid regrowth upon quiescence exit. This indicates that that the role of FAO goes beyond generating citrate for acetyl-CoA- or lipid biosynthesis.

- The authors mention in the discussion (447-448): Together with conventional anti-cancer agents, combination therapies (e.g. with trimetazidine) could simultaneously target highly proliferating and dormant cancer cells, and thereby reduce the risk of cancer recurrence. It would be more relevant if the authors could test this in an in vitro approach, e.g. mimicking tumor heterogeneity by combining proliferating and quiescent cells and treating with combination therapies as suggested. Additionally, since the authors do not provide any in vivo evidence for their mechanism suggesting trimetazidine as a treatment is not justified.

>> We revised the text to clarify this point and avoid any overstatements. Our work sheds light on mechanisms of metabolic adaptation during entry and exit to/from quiescence, using trimetazidine to test and guide the interpretation of our metabolomics data. While collected data suggests for the possibility that TMZ could be an attractive adjuvant of chemotherapeutic treatments, its clinical relevance remain beyond the scope of this study and we stressed this aspect in the conclusions.

- Could this new methodology be applied to characterize differential metabolic profiles on a heterogeneous population? It would be interesting to validate it in vitro by combining different populations that one can find within a tumor, or in the tumor microenvironment (e.g, CAF and cancer cells...)

>> We thank the reviewer for the chance to clarify this point. We revised the text to clarify that we have tested our deconvolution method in a similar way to what was suggested by the reviewer. We used five different cell lines to mimic different cell populations and showed that the deconvolution method can reconstruct the characteristic metabolic differences between the cell lines as previously measured with a standard metabolome profiling approach. To that end, we mixed cell extracts of 2 cell lines at a time, measured ion intensities in the mixed cell extracts and then performed deconvolution. We then compared these reconstructed profiles against the measurements performed previously (Ortmayr et al, 2019) on pure extracts to validate and benchmark the deconvolution method. We have additionally expanded the discussion to specifically comment on the applicability of our approach to the analysis of heterogeneous tissue samples.

- The authors do not discuss current approaches to perform metabolic profiling of quiescent cells. Adding a simplified experiment comparing the new method with one of those and highlight potential advantages would make this approach and hence this manuscript more powerful.

>> The reviewer raises an important point. We now performed new analysis to clarify the difference between the standard approach, i.e. profiling of bulk cell populations, and our deconvolution method (see panel d in Appendix Figure S4). We now also better illustrate the need for an approach that avoids physical separation of cells by experimentally showing that just the trypsinization of adherent cells (a necessary step in any type of physical separation of cell subpopulations) has pleiotropic metabolic effects and introduces additional noise in the data (new Appendix Figure S2). Our approach, by considering aspects that are not typically accounted for, like the fraction and number of cells in each subpopulation, allows generating comparable metabolic signatures characteristic of distinct cell subpopulations, and across multiple cell types and conditions. Of note, only a small number of papers in published literature has performed direct metabolome measurements in quiescence models, and none included multiple cell types, emphasizing the need for alternative approaches enabling a better resolution of cell subpopulations.

Minor comments:

- While the described method and in vitro findings are very compelling, in vivo evidence for the proposed mechanism is missing. The authors at least need to discuss this and point out this limitation in their study. They should also rephrase the text to avoid therapeutic conclusions or conclusions about cancer dormancy

>> We agree with the reviewer. We have expanded the discussion of our approach and findings and revised the text to avoid any overstatements in particular on the clinical relevance, for which further investigations are necessary (see Discussion section). While, indeed, in vivo evidence is needed, genome-based studies have already shown that metabolic shifts in specific cell states exist also in vivo and can be decisive for cell fate and disease progression (e.g. PMID: 34381210).

- The authors should avoid the term dormancy because they do not provide any data on true dormancy in cancer cells

>> We revised the text to clarify this point. We define quiescence as the G_0 cell cycle phase, with 2n DNA- and RNA-contents (see description in section 1), according to seminal literature (PMID: 16509772). It is noteworthy that the distinguishing characteristics (e.g. molecular markers) of quiescence vs. dormancy are somewhat vague in the field. To avoid misleading the reader, we have carefully revised the text to refer only to quiescence as per the above definition.

- Figure 3f-g or lines 264-266: A549 and HCT116 cancer cells under serum starvation conditions, trimetazidine treatment caused drastic dynamic changes in cell confluence and induced cell death in a dose-dependent manner. Is it possible that those changes are related to serum (lipids) starvation rather than quiescence induction?

>> We have now added new experimental data showing that similarly to serum starvation, increased sensitivity to trimetazidine is also seen in cell cultures enriched for G_0 cells in glutamine limitation conditions (Appendix Figure S6).

- Figure 3m seems to be essential to explain the hypothesis about fatty acid degradation playing a role in preparing cells for reversing quiescence but it is poorly explained in the text. It could be better explained since it is a complex figure showing an unconventional output.

>> We thank the reviewer for the constructive feedback, and we have revised the text to improve clarity. Indeed, regrowth of cells after drug perturbation is often overlooked and we showed that it can be crucial to fully capture the effect and mechanistic evidence on drug action. We have revised the explanation in the text to more explicitly explain the factors that need to be considered when assessing lag times. One key aspect is the dependency commonly observed between initial cell number and lag time. Already without any treatments or perturbation, longer lag times are often observed around and below 20% initial cell confluence. To make sure that our conclusions are not biased by differences in cell numbers, we normalized the observed lag times based on reference measurements of untreated cells at different cell densities. Specifically, we showed that trimetazidine treatment caused lag times much longer than what would be expected simply because of lower initial cell numbers.

- The authors show that the main differences in metabolic features are mostly due to the cell state rather than cell type or quiescence stimulus. Are there also any metabolic adaptations that differ when comparing environmental stimuli?

>> This is an interesting point which we now expand on in the main text. We found 31 ions which show effect sizes that exhibit significant differences across quiescence-inducing stimuli (see new Appendix Figure S5).

Reviewer #2:

In this manuscript, the authors describe the metabolism of quiescence by studying multiple different cancer and normal cell lines in proliferating and quiescent states. They use a mixing approach followed by linear regression to determine metabolites that are different when a variety of cell lines are induced into quiescence by one of multiple model systems. They discovered that whether the cells were quiescent or proliferating was the strongest determinant of the metabolic profile. The metabolites elevated in quiescent compared with proliferating cells included metabolites associated with fatty acid degradation. Based on these findings, the authors hypothesized that fatty acid degradation is selectively important for quiescent cells. They found that fatty acid degradation is important for viability of the quiescent cells and their ability to enter the cell cycle from quiescence. They propose that this is a fundamental property of quiescent cells that can be exploited for cancer therapy.

The study is a systematic approach to understanding the consistent changes in metabolism when different cell types are induced into quiescence by different signals. The finding of consistencies is interesting and will be informative for others interested in the quiescent state.

Comments

The authors report a novel sorting-free way to measure metabolite levels in quiescent cells.

There is a real problem and important problem for understanding the metabolism of quiescent cells in vivo. We want to know the metabolism of the stem cells in a mixed population taken straight from a tissue. If we sort for the small number of cells that are quiescent, we perturb the system and its metabolism during the sort and can't trust our data. If we do metabolomics on the whole tissue, we don't know what the contribution was of the quiescent cells.

The authors seem to be claiming that they solved this. But they are working with a system with cultured cells that they can transition to a quiescent condition. They can compare the proliferating and quiescent cells. The linear mixing that they perform may provide some improvement, but fundamentally, they could compare the cells cultured in conditions that generate mostly proliferating or mostly quiescent cells. This approach doesn't solve the problem that scientists trying to study complex tissues have, which is that they don't have a pure population of quiescent cells.

>> The reviewer raises an important aspect, which we now clarify and discuss more thoroughly in the manuscript. We clarify and emphasize that our approach does not require pure cell populations. Note that, as we show in the flow cytometry results in Figure 1, even in vitro quiescence-inducing conditions do not produce pure cell populations, and the degree of enrichment can vary between different cell types and quiescence-inducing stimuli. We now show with additional experimental evidence that experimental procedures in cell sorting protocols are too invasive and induce strong confounding metabolic changes (see Appendix Figure S2). We now also show that simply comparing mixed populations will mask characteristic metabolic differences in quiescence-induced cell populations (see new analysis in Appendix Figure S4).

The authors should be careful to cite previous studies that have also investigated fatty acid oxidation in cells in quiescent states. As one example, the review by Shyh-Chang and Ng in Genes and Development in 2017 provides information on previous findings about the role of fatty acid oxidation in stem cells.

>> We carefully revised the citations and added the suggested reference. Moreover, we clarify how our findings compare to previous studies suggesting a role of FAO in quiescence.

Fig 3l shows the extent of apoptosis and death with TMZ treatment. There are no statistical comparisons shown. There is more death in the treated quiescent cells than the treated full medium cells, but there is also more death and apoptosis in the untreated quiescent than untreated full medium cells. TMZ treatment may have significantly increased apoptosis in the full medium conditions as well as the quiescence conditions.

>> The reviewer addresses an important point which we now clarify. Here, we monitored dynamic changes in cell health and apoptosis in real time and showed that in trimetazidine-treated quiescence-induced cells, apoptosis occurs rapidly and continuously, reaching approximately 60% of cells by 48 hours. In contrast, untreated cells show neither apoptosis nor cell death until approximately 48 hours, where the cultures have already reached full confluence, consistent with cell health being unaffected by trimetazidine. To better visually reflect the dynamic aspect in Figure 3, we have added a new panel which shows the overlay of the time-course data in treated vs. untreated cells.

Figure 3M is a critical figure because the authors argue that it demonstrates that fatty acid oxidation not only causes death of quiescent cells but also a failure to re-enter the cell cycle. Fig 3b and 3c also show a delay in regrowth but it's not clear if that's a failure to proliferate or cell death. In Fig 3M, the authors compare lag times with and without trimetazidine treatment. They do this by monitoring growth at the maximum timepoint and extrapolating back, and comparing to untreated cells.
There are better ways to monitor proliferation at a specific timepoint than doing a backwards interpolation from a later timepoint. The authors could perform Ki-67 or BrdU labeling to determine the fraction of cells dividing.

>> We thank the reviewer for raising this point which we now clarify. The method suggested by the reviewer, while able to more directly measure fractions of dividing cells, is typically applied at single time points and cannot be measured dynamically, thereby requiring a priori knowledge on the onset time for cell divisions in the multiple conditions tested. The herein-estimated lag time is a measure of the time necessary to resume proliferation and can be estimated using data from the entire growth curve dynamics shown in Figure 3c, rather than individual time-points.

Further, the data for A549 and HCT116 cells seem to be telling different stories. For A549 cells, it seems that for doses over 400 microM, there is a similar lag with treatment versus without, while for HCT116, the data seem to show a bigger lag with higher dose. However, these data are somewhat complicated to interpret because the experiment seems to have been performed differently for the two cell lines since for HCT116 but not A549, all of the data have the same expected lag. Can the authors please explain?

>> We have revised the text to more explicitly explain the interpretation of lag time data. Adherent cell lines typically display a dependence of regrowth dynamics (e.g. lag time) on the starting cell density, i.e. the lower the initial cell density, the longer is the lag time. This basal dependency is cell line dependent (Appendix Figure S9). To ensure that our conclusions on the growth dynamics in TMZ-treated cells are not biased by this behavior, we compared lag time in treated vs untreated quiescent cells with a comparable initial cell number (see black lines Appendix Figure S9). To that end, we estimated the lag time that one would expect in untreated cells at the same initial cell density (x-axis value in Figure 3k), and compared it to the lag times estimated from the dynamic growth curves shown in Figure 3c (y-axis value in Figure 3k). The expected lag for HCT116 cultures is similar because HCT116 inherently does not exhibit significant lag times over a wide range of cell densities (see Appendix Figure S9). In contrast, A549 cell show a more graded dependency of lag time on cell density, explaining the greater spread of expected lag times in Figure 3k).
Importantly, in both cell lines and at trimetazidine doses of 400 µM or higher, the observed lag time is significantly higher than the expected lag time in untreated cells.

Overall, we thank the reviewer for the constructive feedback and the opportunity to clarify important aspects of this manuscript.

Reviewer #3:

Summary

Ortmayr and Zampieri show that a panel of four cancer cell lines and two fibroblast cell lines have similar growth dynamics upon induction of quiescence through three different stimuli: glutamine deprivation, serum starvation, and contact inhibition. They utilize a multiple term linear model to deconvolute cell population contributions to a mixed cell metabolome, estimating the relative intracellular abundances of metabolites in proliferating versus quiescent cells. Increased TCA cycle intermediates that are downstream of fatty acid oxidation (FAO) along with other lipid metabolism-associated metabolites in quiescent cells suggested modulation of FAO during quiescence. To test the dependence of quiescence on FAO, the authors inhibited beta oxidation with trimetazidine (TMZ). TMZ had an inhibitory effect on cell regrowth after quiescence release, and reduced cell viability during quiescence induction. Further, signature metabolite abundance changes associated with quiescent cells were blocked by TMZ, and quiescent cultures displayed higher apoptosis.

Overall, this is an interesting manuscript that focuses on an important problem in metabolism: how do we deconvolute the metabolic contributions from different cell types when they are in mixed populations? In this reviewer's perspective, the work represents an interesting step forward; however, it is absolutely critical that the authors clearly highlight the limitations of their approach if they are to publish, which are not trivial as described below.

General remarks

This study confirms other published results that have shown the importance of FAO in endothelial cells, fibroblasts, neural stem cells, leukemia, and melanoma through its use of four additional cancer cell lines (PMID: 31375515, 28854364, 27374788, 20038799, 27049668, 21049082, 30146488). Other studies have suggested redox toxicity after FAO inhibition, which this study did not investigate. Thus, the major and primary advance of this study is technical: in designing a deconvolution method for mixed-cell metabolomics.

>> We thank the reviewer for the opportunity to clarify the novelty aspect. Indeed, here we developed an original approach to detect characteristic differences between co-existing subpopulations, and applied it to investigate characteristic metabolic differences in G_0 cells in multiple cell types and conditions. We found fundamental metabolic differences that were shared among G_0 cells in multiple cell types and conditions, an aspect that had never been explored or observed before.

The reviewer is correct and indeed other studies have suggested different roles for FAO in quiescence. We expanded the discussion on these previous findings. However, there are several aspects, beyond the methodology, that differ and build on previous literature:

- Earlier studies typically considered only one cell type, and to a large extent focused on non-cancer cells. Our metabolome-based analysis uncovered a characteristic difference in the regulation of fatty acid degradation in multiple cell types and quiescence-inducing stimuli.
- While fatty acid beta-oxidation has so far mostly been studied in endothelial- or stem cells and with a focus on its role in energy generation or redox homeostasis, we have now added additional evidence to suggest that FAO during quiescence induction is likely not crucial for energy or redox balance, but rather to mediate homeostasis in degradation of fatty acids and prevent the buildup of toxic intermediates (see new data on citrate/GSH supplementation in Appendix Figure S7 and Appendix Figure S9, and complementary text in section 3).

I have three significant concerns about the technical approach.

First, the authors assume that the total abundance of a metabolite is linearly dependent on the number of cells extracted. This is not necessarily true. The signal of a metabolite in mass spectrometry is not linear with respect to its concentration. Thus, a two-fold change in cell number could correspond to only a small change

in MS signal. I am not sure how their model can address this intrinsic and non-predictable lack of linearity in MS data.

>> The reviewer raises important points which helped us to mature the manuscript. The reviewer is correct in that several parameters can affect the relationship between metabolite concentration and ion intensity. To address this fundamental aspect our procedure identifies and filters out ions that do not show a significant linear dependency. We emphasized this point in the main text and materials and methods section in detail (see below points).
Moreover, we clarify the assumptions that are key for the analysis and interpretation of our metabolic profiling approach, and performed new experiments and analysis to verify that the conditions are met.

Additionally, the signal of a metabolite is dependent upon its matrix. A metabolite standard spiked into one cell extract at a given concentration will give a different signal when it is spiked into another cell extract at the same concentration due to differences in matrix effects. Mixing cell types changes the matrix. The signals measured in pure cell types cannot be quantitatively compared to signals measured from mixed cell types (especially when using FIA). This seems like a serious complication to the approach described.

>> This is an important point, which we now explicitly remark in the main text. We have included new systematic experimental data and analysis to verify and confirm that different cell types do not exhibit large differences in the overall sample matrix. To that end, we performed an experiment similar to what was suggested by the reviewer, where we spiked metabolites in increasing concentrations into cell extracts of different cell lines. Our analysis showed that signal intensities, respectively the dependency of ion intensity on metabolite concentration, is robust to different cell types (see revised text in section 2, and Appendix Figure S3).
This new analysis complements the already included benchmarking data including five different cell lines, which supports this claim as well. Here, each cell line was mixed with up to four different other cell lines and we applied the deconvolution approach to reconstruct the original metabolic profile of each of the 5 cell lines. Our analysis showed that across all different sample mixes, ion intensities aligned into a conserved linear relationship between the number of cells and the measured ion intensity (series of plots at the diagonal in Appendix Figure S4, panel b) for 1850 putatively annotated metabolites. This confirms that even the mixing of different cell lines does not cause a drastic change in the overall sample matrix that could affect the performance of our deconvolution approach. We agree with the reviewer that this is a key prerequisite and have emphasized the need to verify this aspect in the main text.

The second concern is that this approach will only work for a limited number of applications where cells can be purified and measured individually to generate a reference curve. Such analyses will not be possible for tissues. Thus, the method is really limited to a specific set of in vitro studies.

>> We thank the reviewer for the opportunity to clarify this point. Indeed, one key advantage of our approach is that it can be applied on mixed populations without the need for purified cells. We now revised the text to make this fundamental aspect clear.
Our approach does not require pure cell populations. In fact, even in vitro, cultures maintained in full medium (containing FBS) contain a fraction of G_0 cells (see Figure 1). Similarly, quiescence induction does not produce pure populations. Note that, as we show in the flow cytometry results in Figure 1, even in vitro quiescence-inducing conditions do not produce pure cell populations. Avoiding cell sorting procedures to obtain pure cell cultures was indeed one key motivation behind developing the deconvolution approach.
What is really crucial, is a way to modulate the relative proportion of the different subpopulations in the samples before measurement, for which there are different solutions. Here, we were able to enrich cell populations using in vitro conditions (serum starvation, glutamine limitation, contact inhibition). We envisage that in vivo, tissue sections from different sites can already be differentially enriched for subpopulations of interest. For example, outer- and inner regions of a tumor contain cells reprogrammed

by different environments, or tissue sections close to blood- or lymphatic vessels can exhibit greater infiltration of specific immune cells. Such samples would be perfectly suitable to apply the deconvolution approach to separate the metabolic signatures of the underlying cell populations. We now more explicitly discuss this point in the main text (see also discussion section).

The third concern is that the approach assumes that intracellular metabolite levels will not change as a function of cellular environment. In other words, the authors assume that one million pure cells will produce the same amount of glutamate as one million of the same cells mixed with other cell types. However, this might not be true. For example, it is likely that the amount of a given metabolite in a cell will change depending on the density of other sub-populations in which it is cocultured. This is because cells reprogram their metabolism when they are cocultured with different cell types.

>> We now clarify that this is an assumption of our framework. Because our samples always contain a mix of the two subpopulations of interest (G_0 and non-G_0 cells, see also point above) key metabolic interactions should be already and consistently present at all time points in our analysis. The fact that only 8% of putatively annotated ions did not allow fitting a significant linear relationship in at least one cell line is supporting this claim.

Recommendations
1. The most important revision that I urge the authors to consider is discussing the technical limitations above. They may not all be solvable, but the authors should at least discuss them in detail so that they do not mislead readers. For example, the title should highlight that this is an in vitro technique only. Perhaps "An in vitro sorting-free profiling technique", or something similar. The abstract should point out that the technique is not compatible with tissues. Each of the limitation points above should be described in detail in the discussion. Conclusions should be tempered based on these points. Readers should be cautioned of the assumptions being made and when they might break down (eg, maybe low abundance metabolites are more susceptible to non-linearity issues).

>> We thank the reviewer for the constructive feedback. We have now revised the text to clarify the potential, assumptions and limitations of our approach (see also points above).

2. While the authors did assess a handful of cell types and observe similar patterns, drawing general conclusions about quiescence induction and release dynamics is risky. Such a claim would require experiments with many more non-fibroblast and non-cancer cell types. Even when generalizing about cancers from different tissues, several cell lines from each tissue should be utilized before generalizations are made. I recommend removing the claim that the observations are broadly applicable to all cell types rather than trying to prove this, which would require substantial experimental effort.

>> We revised the text to avoid any overstatement. In principle adding more cell lines, or conditions is always desirable, but the question is how much would be enough. Our study comprises 6 largely diverse cell lines and 3 radically different stimuli, and the extent to which the quiescence-related metabolic characteristics were shared across cell types and stimuli is remarkable and never observed before. Hence, we think that the degree of metabolic commonality is a relevant and novel aspect of our work worth to be discussed and expanded on in future work.

3. Quiescence release growth dynamics and apoptosis induction in quiescent cells by TMZ should be re-measured after isolation of quiescent cells via flow sorting to confirm that the dynamics are not due to a large population of non-quiescent cells continuing to proliferate and that the observed apoptosis is indeed in sensitized quiescent cells and not proliferating cells

>> The reviewer raises an important point. This is an experiment we have also considered ourselves.

However, it is technically not trivial. Sorting procedures are lengthy and presents intense and potentially lethal stress for the cells, beginning with enzymatic cell detachment, and further including repeated physical stress (e.g. centrifugation, shear stress and electromagnetic forces during flow sorting) and exposure to low-nutrient conditions (e.g. PBS, low O_2 supply due to closed vessels). Hence we think that results from such experiments will be non-conclusive.

4. The authors validate their method by comparing computationally derived results to known mixtures of independently grown cultures; however, since the computational method is based on dilution of known pure cell population metabolite extracts, this validation method is circular.

>> We revised the text to better clarify the experimental setup for the benchmarking of our procedure. The key point here is that the deconvolution method does not use measurements of pure cell populations as an input (see also points above). In the benchmarking experiment, we generated mixed samples containing cell extracts of two different cell lines, which mimic co-occurring G_0- and non-G_0 cells. The advantage is that in the case of these new mixed samples, we know the characteristic metabolic differences between the mixed cell types, and can compare the deconvolution result to earlier measurements of relative differences in metabolite abundances (Ortmayr et al, 2019) to confirm the validity of the deconvolution procedure.

The authors do not benchmark their method against other computational methods for deconvolution, so it is unclear how significant this advance is compared to previous methods (eg, 10.1109/BIBM.2016.7822519). They may wish to compare their method to the previously published one if they find that it is relevant to their system.

>> We thank the reviewer for pointing us to this paper. There are few technical and conceptual differences between our and the cited approach. The most important is that in the work mentioned by the reviewer, the approach is meant to denoise the signals from “contaminating” populations of cells, the number of which is not directly quantified and that represents only a minor fraction of the entire population. The authors use a semi-supervised probabilistic approach. Our approach instead is based on the quantification of cells in the different subpopulations which allows to use a more quantitative model design and estimates of metabolic parameters based on regression analysis. Moreover, if we understand correctly, in the suggested framework one needs multiple samples from almost pure populations of the same type. Our approach also takes into account and preserves aspects like cell type specificity, precluding the possibility to use the cited approach directly on our data.

5. To validate their computational model, the authors may want to test a known metabolic change in a mixed cell population (eg, delivering an inhibitor to which one cell line is sensitive and the other is resistant with a previously published metabolic phenotype, or utilizing an inducible-oncogene cell line in which oncogene induction has metabolic effects combined with a cell type that has no reaction to the induction stimulus). Without such validation using previously published phenotypes or methods, it is difficult to truly accept the results produced by the computational model.

>> Model validation is indeed an important point, and what suggested by the reviewer is largely consistent with the validation presented. We had characterized the metabolic differences between the 5 cell lines in an earlier, independent study, and showed here that we can recover the significant differences in metabolite abundance between the cell lines, equivalent to metabolic differences induced by an inhibitor or oncogene induction. Using an inhibitor to validate our results generally seems a more difficult strategy. Inhibitors can induce multiple (also indirect) metabolic and phenotypic changes, making data interpretation and analysis complicated and possibly unconclusive. Our strategy provides solid evidence to support model-based predictions.

6. The authors write in the methods section that metabolites were only putatively annotated. This is particularly problematic in FIA where there are no retention times to compare to. The identity of metabolites should be confirmed with fragmentation data.

>> We have performed additional LC-MS/MS measurements to confirm the major metabolic changes leading to our core biological conclusions (see Appendix Figure S7), both with respect to G_0 vs. non-G_0 and to confirm the effect of trimetazidine treatment.

Minor points

1. What is the authors' explanation for the greater proportion of proliferating cells in CCD1070Sk cultures exposed to glutamine deprivation over other stimuli (Fig 1C) but the significantly lower (even reduced) confluence over time (Fig 1d)?

>> The reviewer points to an important aspect. Whether or not cells enter G_0 cannot be concluded from cell confluence alone, which is why we use the flow cytometry assay to specifically quantify the G_0 fractions in each cell line and condition. A key difference in the interpretation is that cell confluence estimates the total number of cells, while flow cytometry informs on the relative proportion of subpopulations within a population.

2. The authors should depict how cell number estimates are acquired in their graphical representation of the data acquisition workflow (Fig 2A).

>> We have modified the schematic in Figure 2a to include both cell counting and the quantification of G_0 fractions by flow cytometry, to reflect all key inputs to the model.

3. The authors should illustrate the complete Intensity equation, including volume parameters (even though these were taken to be equal for all cell types) to accurately represent the model's inputs to the reader.

>> We have modified the intensity equations in Figure 2b and Appendix Figure S4a accordingly.

4. The authors may want to clarify the explanation for Fig 2C as this reviewer finds it currently difficult to follow. For example, what is the estimated density of correlations and how was it acquired? Is this a histogram?

>> We thank the reviewer for the feedback and have revised the text and figure caption to explain this better. This figure shows that we found high correlation between relative metabolite abundances estimated by deconvolution and earlier reference measurements for a large number of metabolites. We present the distribution of correlation coefficients for all metabolites that showed significant variation across the five cell lines, which is shifted towards high correlations (median 0.7, Spearman). We report this distribution as probability densities estimated using a kernel smoothing function. To estimate the statistical significance, we show distributions that could be expected at random by calculating correlations after scrambling cell line labels in the reference dataset, or after selecting the same number of metabolites at random.

5. Effect size or fold change information should be depicted in Fig 2F for the reader to be able to judge the biological significance of metabolite abundance changes outside of their statistical significance and association.

>> Figure 2f focuses on analyzing the similarities of metabolic differences between G_0- and non-G_0 cells across cell types and conditions. We present the underlying changes in abundance (effect sizes) across cell types and conditions in Figure 2g exemplarily for the most prominent metabolic changes which we also mention in the text, and for all 1940 putatively annotated metabolites in all cell types and conditions in the Datasets section. We use effect size as a measure of metabolic change, which is the difference in the mean relative metabolite abundance between G_0 and non-G_0 cells, divided by the pooled standard deviations of

the two abundance estimates. Hence, the effect size not only quantifies the abundance change, but also relates it to the technical/experimental error (see methods section for full detail).

6. How do the authors account for the outliers in Fig 2G? Were biological and technical replicates measured? If not already, they should be.

>> Our analysis always takes into account variations across 3 biological replicates. The estimates of the two model parameters have an associated error which reflects variations among replicates and is used in the calculation of effect sizes (see also point 5 above).

7. Why is a continuous plot not shown for the data in Fig 3d and Fig 3e? It currently appears as if the data do not line up from one plot to another.

>> We had chosen to separate the two phases for figure referencing reasons, because the time segments before and after 96 hours inform on distinct aspects of the quiescence phenotype, i.e. quiescence entry and regrowth, respectively. It is important to note that the figure showed the full continuous data already. The decrease in confluence at 96 hours is explained by detached and dead cells being rinsed away when we replace the medium in each well by full growth medium to stimulate regrowth, consistent with the viability data shown in Figure 3d. We have revised the text and figure to re-assemble the continuous timeline.

8. The Figure 3 legend has incorrect lettering progression (letter j is repeated where letter i should be).

>> We have amended the Figure labels and caption.

9. Why did the authors not measure metabolite abundance at 96 hours post-TMZ when a larger phenotypic difference is observed?

>> We have indeed measured metabolic differences at 96 hours, they were reported in Supplementary Figure 4, now Appendix Figure S8.

10. The explanation for the scatter plot in Fig 3K should be clearer. What are the depicted fold changes relative to? Are they proliferating vs quiescent cells?

>> The fold-changes are relative to the steady state in proliferating cells. This is because it allows contrasting changes related to quiescence per se from changes related to the additional trimetazidine treatment. We have described this in the Methods section, and have revised the figure caption to better indicate this.

11. I also recommend that the authors re-work the manuscript title to reflect that their method is used to validate the importance of FAO to quiescence, which has been published before (eg, perhaps something like "An in vitro sorting-free profiling technique confirms the importance of...".

>> We have considered the reviewer's comment and revised the title. However, as already discussed at the beginning, it is important to note though that while FAO has been associated with quiescence before, here we first show that this is a metabolic characteristic and a vulnerability in quiescence across diverse cell types and three quiescence-inducing conditions, and secondly suggested a potentially additional role in mediating transitioning into quiescence.

12. Would the authors please confirm with literature citations that all cancer cell lines used are contact-inhibited? If some are not, do they display differing levels of quiescence induction?

>> We are not completely sure about what the reviewer is asking here. We measured the fraction of G_0 cells in all cell lines after 96 hours contact inhibition (Figure 1) and have not noted a strict dependence of G_0 fractions on a specific cell type.

13. Would the authors please clarify the methods section about how the mass spectrometry was run? Currently it suggests that a liquid chromatography instrument was used despite the method citing flow-infection TOFMS.

>> It is important to avoid confusion on this point. All instrumental components that we use are part of an LC-MS instrument and we indicate the name as specified by the vendor. This is different from the mode of operation, where we indicated a flow injection-based methodology with electrospray ionization, including the original literature reference.

Overall, we thank the reviewer for the input and constructive feedback, and the opportunity to clarify important aspects of this manuscript.

RE: MSB-2021-10716R, Sorting-free metabolic profiling uncovers the vulnerability of fatty acid β-oxidation in quiescence

Thank you for sending us your revised manuscript. My apologies for the delay, but as Chris wrote to you, I was out of the office on holiday for two weeks. We have heard back from the reviewers who agreed to evaluate your revised study. As you will see below, they think that the study has improved as a result of the performed revisions. Reviewers #2 and #3 suggest some further minor revisions, mostly referring to text edits. Regarding the point of reviewer #3 requesting the removal of all statements related to "in vivo" and "clinical", we do not think that this is necessary, provided that the related statements are balanced and clearly mention potential limitations etc. We would ask you to address these remaining issues (listed below) in a last minor revision.

Let me know if there is anything you would like to discuss before preparing the revision.

We would also ask you to address some remaining editorial issues listed below:

Reviewer #1:

The authors have significantly improved the manuscript.
It is very interesting that they propose that toxic intermediates of FAO accumulate. Showing this accumulation and the concomitant decrease upon etomoxir treatment would strengthen the mechanistic conclusions. In line the overview figure 3A should be adapted.

Reviewer #2:

I thank the authors for responding to my questions and the questions from the other reviewers. Some key points remain:

Reviewer 3 suggested 2 changes to the title, first acknowledging that this approach is an in vitro approach and something to acknowledge the previous studies showing fatty acid oxidation is important with quiescence.

On line 163, the authors write that the abundance of a metabolite is linearly dependent on the number of cells extracted, but Reviewer 3 noted that it's not possible to make that assumption.

Line 466: says that the system is applicable to any system. Line 471 talks about using this in a clinical context.
Without some way of knowing what profile goes with what cell, it seems like a very big jump from cultured cells that have different and controllable amounts of quiescent versus proliferating cells or mixtures of 2 cancer cell lines to complex mixtures of tissue in which a scientist does not know what cells are present, in what abundance, and what their metabolic profiles are.

Reviewer #3:

I suggest the authors strike all mention of "in vivo" or "clinical" unless they are willing to discuss or execute strategies for that application. The authors propose in their rebuttal to use tissue sections from different sites that have different cell type enrichments; however, the presupposition that different areas have different relative cell populations precludes the use of the authors' computational method since this method requires two identical samples to run in parallel through the flow and metabolomics pipelines.

The authors should revise the manuscript with the MS nonlinear dependence caveat at line 164. Further, the paragraph starting at line 191 does not resolve or test the first assumption introduced on line 168 which was our third major concern in our initial review. Line 167 should be amended to reflect this concern, e.g. "in a mixed cell population, the total measured metabolite abundance is a linear combination of the characteristic metabolite abundances in each individual cell subpopulation, provided that the presence or relative abundance of each distinct population in culture does not affect the metabolism or general cell biology of the other in a way that might change the amount of any given metabolite per cell". The authors might then wish to elaborate that changes in cell size, shape, metabolic activity, signaling influencing metabolic activity, growth rate, organelle size or health, and nutritional uptake/availability would all potentially cause this critical assumption to fail. The authors attempt to resolve this concern by noting high degrees of linearity across their extract mixing spectrum, but our concern lies with the metabolism of cells before metabolites have even been extracted (i.e. when still in culture). It is necessary to show that different proportions of G0 to proliferating cells within the dish has no relevant biological effect on either population in order to utilize this

method, and to discuss that this assumption has already been shown to break down in notable cases like the tumor microenvironment.

If the authors wish to generalize their conclusions to quiescence in general, they should test cell lines that are non-cancerous and also not fibroblasts to ensure that their limited sample size does not lead to faulty conclusions. The data argue most compellingly for conclusions about "cancer quiescence" currently; however, even that claim is questionable without more data.

Further minor comments:
How reproducible are the proportions shown in Fig 1C between batches of quiescence induction? A high degree of reproducibility would be required to map flow-determined G0 vs proliferating cell counts onto separate batches of cells used for metabolomics with sufficient confidence to call the method of mixing differently enriched extracts plausible.
Addressing the rebuttal in #3: if the process of flow sorting is so damaging to the cells that accurate evaluation of populations after measuring growth dynamics is not possible, then the authors should reword their conclusions to reflect the uncertainty in the existing data, and scrutinize the use of this technique in the central deconvolution method of the paper.
Addressing the rebuttal in #5: I disagree that the proposed experiment matches the validation already completed in the paper, and reiterate that the validation method used in the paper seems inappropriate as it uses pure cell populations and relies on the mixing of cell extracts to supposedly replicate a mixed cell population. If inhibitors seem too messy, perhaps the inducible oncogene approach would be more favored. I recommend completing this additional validation to be confident in the success of the computational method.
The authors' response to Minor Point #1 requires further clarification. I am confused at how the higher proportion of proliferating cells in the glutamine deprivation condition relative to serum starvation in the CCD1070Sk cells, as established by flow cytometry, does not result in a higher confluence over time compared to serum starvation which has a lower proportion of proliferating cells; rather, serum starvation shows markedly higher proliferation as evidenced by the increase in confluency over time. Do the authors propose that the cells labeled as proliferating by flow cytometry exhibit a different proliferative rate in glutamine deprivation than those under serum starvation?
Addressing the rebuttal in Minor Point #11: While I'm not aware of any paper demonstrating the importance of FAO during the transition from proliferation to quiescence, induction of FAO in quiescent cells has been thoroughly established in many cell types. Further, the vulnerability of FAO in quiescent cells has been established in at least one paper pertaining to Leukemia, so this aspect is not entirely novel either.

Reviewer comments:

Reviewer #1:

The authors have significantly improved the manuscript.
It is very interesting that they propose that toxic intermediates of FAO accumulate. Showing this accumulation and the concomitant decrease upon etomoxir treatment would strengthen the mechanistic conclusions. In line the overview figure 3A should be adapted.
>> We thank the reviewer for the positive assessment of the changes we introduced. It is worth noting that etomoxir is an indirect inhibitor of FAO, in that it limits the transport of long-chain fatty acids across the mitochondrial membrane. Cells can bypass such a limitation by diffusion of short-chain fatty acids, or peroxisomal FAO, as we note in section 3. Moreover, the data presented in a previous study characterizing etomoxir's action suggests that FAO inhibition by etomoxir is incomplete (PMID: 30043752). Hence etomoxir, when combined with trimetazidine, showed residual toxicity (Figure 3d) and accumulation of citrate (Appendix Figure S7) and only a delay in apoptosis induction (Appendix Figure S8).

Reviewer #2:

I thank the authors for responding to my questions and the questions from the other reviewers. Some key points remain:

Reviewer 3 suggested 2 changes to the title, first acknowledging that this approach is an in vitro approach and something to acknowledge the previous studies showing fatty acid oxidation is important with quiescence.
>> We changed the title to accommodate the reviewer's request.

On line 163, the authors write that the abundance of a metabolite is linearly dependent on the number of cells extracted, but Reviewer 3 noted that it's not possible to make that assumption.
>> The statement in line 163 refers to the theoretical dependency between sample intake/extracted biomass (e.g. number of cells) and signal response (i.e. ion intensity) in MS-based metabolomics independent of the mixing setup. In previous work, we have demonstrated that by using our experimental and analytical approach (PMID: 31015463) a large fraction of ions exhibits a linear relationship in a wide range of physiological concentrations. Here we reinforced these findings by adding extensive experimental data that demonstrates linearity (see Appendix Figure S3). We also provide direct experimental evidence that the same linear relationship holds true in different cell lines, ruling out possible confounding matrix effects in the data analysis and interpretation.

Line 466: says that the system is applicable to any system. Line 471 talks about using this in a clinical context.
Without some way of knowing what profile goes with what cell, it seems like a very big jump from cultured cells that have different and controllable amounts of quiescent versus proliferating cells or mixtures of 2 cancer cell lines to complex mixtures of tissue in which a scientist does not know what cells are present, in what abundance, and what their metabolic profiles are.
>> The purpose of our methodology is to uncover characteristic profiles of different cell sub-populations without prior knowledge of their molecular (metabolic) profiles. We revised the text to make sure that we do not claim nor is it intended that the methodology could discover entirely unknown sub-populations *de novo*. Indeed, knowing the relative proportion of cell types of interest is an essential requirement (see

discussion section). New techniques, like single-cell gene expression analysisPMID: 35469013), cell painting (PMID: 29153976), or CyTOF (PMID: 35363540) might partially solve this problem and guide the characterization and quantification of relevant cell sub-types also in complex clinical samples.

Reviewer #3:

I suggest the authors strike all mention of "in vivo" or "clinical" unless they are willing to discuss or execute strategies for that application. The authors propose in their rebuttal to use tissue sections from different sites that have different cell type enrichments; however, the presupposition that different areas have different relative cell populations precludes the use of the authors' computational method since this method requires two identical samples to run in parallel through the flow and metabolomics pipelines.

>> In the Discussion section, we further clarify how we envisage the approach to be applied beyond the in vitro scope of this study. We now provide more context on the potential role that our approach could play in clinical research with the aim to indicate to the reader what would be necessary to take the next step. On a technical note, the collection of flow cytometry samples is not a strict limitation, since typically only small amounts of cells are needed for metabolome measurements, allowing to split the sample for flow cytometry and metabolomics in parallel.

The authors should revise the manuscript with the MS nonlinear dependence caveat at line 164. Further, the paragraph starting at line 191 does not resolve or test the first assumption introduced on line 168 which was our third major concern in our initial review. Line 167 should be amended to reflect this concern, e.g. "in a mixed cell population, the total measured metabolite abundance is a linear combination of the characteristic metabolite abundances in each individual cell subpopulation, provided that the presence or relative abundance of each distinct population in culture does not affect the metabolism or general cell biology of the other in a way that might change the amount of any given metabolite per cell". The authors might then wish to elaborate that changes in cell size, shape, metabolic activity, signaling influencing metabolic activity, growth rate, organelle size or health, and nutritional uptake/availability would all potentially cause this critical assumption to fail. The authors attempt to resolve this concern by noting high degrees of linearity across their extract mixing spectrum, but our concern lies with the metabolism of cells before metabolites have even been extracted (i.e. when still in culture). It is necessary to show that different proportions of G0 to proliferating cells within the dish has no relevant biological effect on either population in order to utilize this method, and to discuss that this assumption has already been shown to break down in notable cases like the tumor microenvironment.

>> We agree with the reviewer that this is an important point. We amply discussed it in the revised manuscript and we additionally revised the text as suggested. It is worth noting that because in our method the two different cell extracts used in the mixing scheme are derived from cultures containing both sub-populations already (see Figure 1c), potential interactions between sub-populations are likely present in the samples.

If the authors wish to generalize their conclusions to quiescence in general, they should test cell lines that are non-cancerous and also not fibroblasts to ensure that their limited sample size does not lead to faulty conclusions. The data argue most compellingly for conclusions about "cancer quiescence" currently; however, even that claim is questionable without more data.

>> We have carefully re-inspected all relevant statements to avoid overstatements. We state in the discussion section that further investigations using more cell lines and conditions are needed to support further generalization of our findings.

Further minor comments:
How reproducible are the proportions shown in Fig 1C between batches of quiescence induction? A high degree of reproducibility would be required to map flow-determined G0 vs proliferating cell counts onto separate batches of cells used for metabolomics with sufficient confidence to call the method of mixing differently enriched extracts plausible.
>> We report the repeatability precision of flow cytometry measurements in Appendix Figure S1, showing less than 15% variation across replicates for all cell cycle phases (on average 12% for G_0 fractions) and good agreement between different cultivation formats (i.e. 6-well plate and T75 cell culture flask).

Addressing the rebuttal in #3: if the process of flow sorting is so damaging to the cells that accurate evaluation of populations after measuring growth dynamics is not possible, then the authors should reword their conclusions to reflect the uncertainty in the existing data, and scrutinize the use of this technique in the central deconvolution method of the paper.
>> This is an important point which we clarified in the revised manuscript. We discuss the problems with cell sorting in metabolomics, which differently from transcriptomics or proteomics is highly sensitive to even short perturbations in a fraction of seconds (PMID: 23455438), while significant changes in the abundance of transcripts and proteins require minutes. We indeed added new experimental evidence that show how the very first necessary step for flow cytometry of adherent cells, i.e. trypsin-mediated detachment, can cause severe metabolic changes. Our results and conclusions are also consistent with previous findings (PMID: 30362351, PMID: 29627745). It is worth noting that in part for these technical limitations, previous studies reporting metabolic characteristics associated with quiescence mostly relied on bulk metabolite measurements without prior cell separation, or used transcriptome- or proteome measurements altogether. Hence, our approach, by avoiding unwanted perturbations inevitably linked to the cell sorting procedures, offers a more direct strategy to probe metabolism of unperturbed coexisting cell subpopulations.

Addressing the rebuttal in #5: I disagree that the proposed experiment matches the validation already completed in the paper, and reiterate that the validation method used in the paper seems inappropriate as it uses pure cell populations and relies on the mixing of cell extracts to supposedly replicate a mixed cell population. If inhibitors seem too messy, perhaps the inducible oncogene approach would be more favored. I recommend completing this additional validation to be confident in the success of the computational method.
>> Under the assumptions clarified and discussed in the manuscript our experiment is able to test the feasibility and accuracy of the approach to deconvolute the signals of co-occurring cell types. We clarify this in the text.

The authors' response to Minor Point #1 requires further clarification. I am confused at how the higher proportion of proliferating cells in the glutamine deprivation condition relative to serum starvation in the CCD1070Sk cells, as established by flow cytometry, does not result in a higher confluence over time compared to serum starvation which has a lower proportion of proliferating cells; rather, serum starvation shows markedly higher proliferation as evidenced by the increase in confluency over time. Do the authors propose that the cells labeled as proliferating by flow cytometry exhibit a different proliferative rate in glutamine deprivation than those under serum starvation?
>> We thank the reviewer for clarifying the question. Please note that the confluence dynamics alone do not allow conclusions on quiescence, and not entering G_0 does not imply that cells continue actively undergoing cell division (i.e. proliferation). This is also why we do not refer to non-G_0 cell subpopulations as "proliferating". Furthermore, the mentioned difference in confluence dynamics between serum starvation and glutamine limitation are likely due to different factors becoming growth limiting in the two conditions.

Addressing the rebuttal in Minor Point #11: While I'm not aware of any paper demonstrating the importance of FAO during the transition from proliferation to quiescence, induction of FAO in quiescent cells has been thoroughly established in many cell types. Further, the vulnerability of FAO in quiescent cells has been established in at least one paper pertaining to Leukemia, so this aspect is not entirely novel either.

>> The reviewer is right, FAO is repeatedly found in the literature to associate with quiescence or phenotypically similar cell states, although often based on indirect evidence, and we amply discussed and cited relevant studies. Typically studies that relate FAO to quiescence focus on one/few specific cell types and conditions, and functionality and mechanisms are often not fully investigated. Furthermore, a great challenge in the field is the existence of multiple definitions and terminology to refer to quiescent, dormant or persister cancer cells, and many studies are based on phenotypic observations rather than defined molecular markers. Here, by systematically characterizing metabolic differences between non-G_0 and G_0 cells across different cell types and quiescence inducing stimuli, our work can serve as a basis to formulate predictive models as well as general rules that provide a framework for mechanistic understanding and translational pharmacological interference with the ability of cancer to switch back and forth from quiescence.

RE: MSB-2021-10716RR, Sorting-free metabolic profiling uncovers the vulnerability of fatty acid β-oxidation in in vitro quiescence models

Thank you again for sending us your revised manuscript. We are now satisfied with the modifications made and I am pleased to inform you that your paper has been accepted for publication.

EMBO Press Author Checklist

Corresponding Author Name: Mattia Zampieri
Journal Submitted to: Molecular Systems Biology
Manuscript Number: MSB-2021-10716

USEFUL LINKS FOR COMPLETING THIS FORM
The EMBO Journal - Author Guidelines
EMBO Reports - Author Guidelines
Molecular Systems Biology - Author Guidelines
EMBO Molecular Medicine - Author Guidelines

Reporting Checklist for Life Science Articles (updated January
This checklist is adapted from Materials Design Analysis Reporting (MDAR) Checklist for Authors. MDAR establishes a minimum set of requirements in transparent reporting in the life sciences (see Statement of Task: 10.31222/osf.io/9sm4x). Please follow the journal's guidelines in preparing your
Please note that a copy of this checklist will be published alongside your article.

Abridged guidelines for figures
1. Data
The data shown in figures should satisfy the following conditions:
- the data were obtained and processed according to the field's best practice and are presented to reflect the results of the experiments in an accurate and unbiased manner.
- ideally, figure panels should include only measurements that are directly comparable to each other and obtained with the same assay.
- plots include clearly labeled error bars for independent experiments and sample sizes. Unless justified, error bars should not be shown for technical
- if n<5, the individual data points from each experiment should be plotted. Any statistical test employed should be justified.
- Source Data should be included to report the data underlying figures according to the guidelines set out in the authorship guidelines on Data

2. Captions
Each figure caption should contain the following information, for each panel where they are relevant:
- a specification of the experimental system investigated (eg cell line, species name).
- the assay(s) and method(s) used to carry out the reported observations and measurements.
- an explicit mention of the biological and chemical entity(ies) that are being measured.
- an explicit mention of the biological and chemical entity(ies) that are altered/varied/perturbed in a controlled manner.
- the exact sample size (n) for each experimental group/condition, given as a number, not a range;
- a description of the sample collection allowing the reader to understand whether the samples represent technical or biological replicates (including how many animals, litters, cultures, etc.).
- a statement of how many times the experiment shown was independently replicated in the laboratory.
- definitions of statistical methods and measures:
  - common tests, such as t-test (please specify whether paired vs. unpaired), simple χ2 tests, Wilcoxon and Mann-Whitney tests, can be unambiguously identified by name only, but more complex techniques should be described in the methods section;
  - are tests one-sided or two-sided?
  - are there adjustments for multiple comparisons?
  - exact statistical test results, e.g., P values = x but not P values < x;
  - definition of 'center values' as median or average;
  - definition of error bars as s.d. or s.e.m.

Please complete ALL of the questions below.
Select "Not Applicable" only when the requested information is not relevant for your study.

Materials

Newly Created Materials	Information included in the manuscript?	In which section is the information available? (Reagents and Tools Table, Materials and Methods, Figures, Data Availability Section)
New materials and reagents need to be available; do any restrictions apply?	Not Applicable	

Antibodies	Information included in the manuscript?	In which section is the information available? (Reagents and Tools Table, Materials and Methods, Figures, Data Availability Section)
For **antibodies** provide the following information:<br>- Commercial antibodies: RRID (if possible) or supplier name, catalogue number and or/clone number<br>- Non-commercial: RRID or citation	Not Applicable	

DNA and RNA sequences	Information included in the manuscript?	In which section is the information available? (Reagents and Tools Table, Materials and Methods, Figures, Data Availability Section)
**Short novel DNA or RNA including primers, probes**: provide the sequences.	Not Applicable	

Cell materials	Information included in the manuscript?	In which section is the information available? (Reagents and Tools Table, Materials and Methods, Figures, Data Availability Section)
**Cell lines:** Provide species information, strain. Provide accession number in repository **OR** supplier name, catalog number, clone number, and/**OR** RRID.	Yes	Materials and Methods
**Primary cultures:** Provide species, strain, sex of origin, genetic modification status.	Not Applicable	
Report if the cell lines were recently **authenticated** (e.g., by STR profiling) and tested for mycoplasma contamination.	Yes	Materials and Methods

Experimental animals	Information included in the manuscript?	In which section is the information available? (Reagents and Tools Table, Materials and Methods, Figures, Data Availability Section)
**Laboratory animals or Model organisms:** Provide species, strain, sex, age, genetic modification status. Provide accession number in repository **OR** supplier name, catalog number, clone number, **OR** RRID.	Not Applicable	
**Animal observed in or captured from the field:** Provide species, sex, and age where possible**.**	Not Applicable	
Please detail **housing and husbandry conditions**.	Not Applicable	

Plants and microbes	Information included in the manuscript?	In which section is the information available? (Reagents and Tools Table, Materials and Methods, Figures, Data Availability Section)
**Plants:** provide species and strain, ecotype and cultivar where relevant, unique accession number if available, and source (including location for collected wild specimens).	Not Applicable	
**Microbes:** provide species and strain, unique accession number if available, and source.	Not Applicable	

Human research participants	Information included in the manuscript?	In which section is the information available? (Reagents and Tools Table, Materials and Methods, Figures, Data Availability Section)
If collected and within the bounds of privacy constraints report on age, sex and gender or ethnicity for all study participants.	Not Applicable	

Core facilities	Information included in the manuscript?	In which section is the information available? (Reagents and Tools Table, Materials and Methods, Figures, Data Availability Section)
If your work benefited from core facilities, was their service mentioned in the acknowledgments section?	Yes	Acknowledgements

Design

Study protocol	Information included in the manuscript?	In which section is the information available? (Reagents and Tools Table, Materials and Methods, Figures, Data Availability Section)
If study protocol has been **pre-registered, provide DOI in the manuscript**. For clinical trials, provide the trial registration number **OR** cite DOI.	Not Applicable	
Report the **clinical trial registration number** (at ClinicalTrials.gov or equivalent), where applicable.	Not Applicable	

Laboratory protocol	Information included in the manuscript?	In which section is the information available? (Reagents and Tools Table, Materials and Methods, Figures, Data Availability Section)
Provide DOI OR other citation details if **external detailed step-by-step protocols** are available.	Not Applicable	

Experimental study design and statistics	Information included in the manuscript?	In which section is the information available? (Reagents and Tools Table, Materials and Methods, Figures, Data Availability Section)
Include a statement about **sample size** estimate even if no statistical methods were used.	Yes	Materials and Methods
Were any steps taken to minimize the effects of subjective bias when allocating animals/samples to treatment (e.g. **randomization procedure**)? If yes, have they been described?	Not Applicable	
Include a statement about **blinding** even if no blinding was done.	Not Applicable	
Describe **inclusion/exclusion criteria** if samples or animals were excluded from the analysis. Were the criteria pre-established?<br><br>If sample or data points were omitted from analysis, report if this was due to attrition or intentional exclusion and provide justification.	Yes	Data points with obvious technical defects or not meeting rational filter criteria were excluded, e.g. outliers or MS measurements exceeding the saturation limit. A justification for all criteria is provided in the Materials and Methods section.
For every figure, are **statistical tests** justified as appropriate? Do the data meet the assumptions of the tests (e.g., normal distribution)? Describe any methods used to assess it. Is there an estimate of variation within each group of data? Is the variance similar between the groups that are being statistically compared?	Yes	Statistical procedures are described in the figure captions, and details are provided in the Materials and Methods section.

Sample definition and in-laboratory replication	Information included in the manuscript?	In which section is the information available? (Reagents and Tools Table, Materials and Methods, Figures, Data Availability Section)
In the figure legends: state number of times the experiment was **replicated** in laboratory.	Not Applicable	
In the figure legends: define whether data describe **technical or biological replicates**.	Yes	Biological replicates are indicated in figure captions and the Materials and Methods section.

Ethics

Ethics	Information included in the manuscript?	In which section is the information available? (Reagents and Tools Table, Materials and Methods, Figures, Data Availability Section)
Studies involving **human participants**: State details of **authority granting ethics approval** (IRB or equivalent committee(s), provide reference number for approval.	Not Applicable	
Studies involving **human participants**: Include a statement confirming that **informed consent** was obtained from all subjects and that the experiments conformed to the principles set out in the WMA Declaration of Helsinki and the Department of Health and Human Services Belmont Report.	Not Applicable	
Studies involving **human participants:** For publication of **patient photos**, include a statement confirming that consent to publish was obtained.	Not Applicable	
Studies involving experimental **animals**: State details of **authority granting ethics approva**l (IRB or equivalent committee(s), provide reference number for approval. Include a statement of compliance with ethical regulations.	Not Applicable	
Studies involving **specimen and field samples:** State if relevant **permits** obtained, provide details of authority approving study; if none were required, explain why.	Not Applicable	

Dual Use Research of Concern (DURC)	Information included in the manuscript?	In which section is the information available? (Reagents and Tools Table, Materials and Methods, Figures, Data Availability Section)
Could your study fall under dual use research restrictions? Please check biosecurity documents and list of **select agents and toxins** (CDC): https://www.selectagents.gov/sat/list.htm	Not Applicable	
If you used a select agent, is the security level of the lab appropriate and reported in the manuscript?	Not Applicable	
If a study is subject to dual use research of concern regulations, is the name of the **authority granting approval and reference number** for the regulatory approval provided in the manuscript?	Not Applicable	

Reporting

The MDAR framework recommends adoption of discipline-specific guidelines, established and endorsed through community initiatives. Journals have their own policy about requiring specific guidelines and recommendations to complement MDAR.

Adherence to community standards	Information included in the manuscript?	In which section is the information available? (Reagents and Tools Table, Materials and Methods, Figures, Data Availability Section)
State if relevant guidelines or checklists (e.g., **ICMJE, MIBBI, ARRIVE, PRISMA**) have been followed or provided.	Not Applicable	
For **tumor marker prognostic studies,** we recommend that you follow the **REMARK** reporting guidelines (see link list at top right). See author guidelines, under 'Reporting Guidelines'. Please confirm you have followed these guidelines.	Not Applicable	
For **phase II and III randomized controlled trials**, please refer to the **CONSORT** flow diagram (see link list at top right) and submit the CONSORT checklist (see link list at top right) with your submission. See author guidelines, under 'Reporting Guidelines'. Please confirm you have submitted this list.	Not Applicable	

Data Availability

Data availability	Information included in the manuscript?	In which section is the information available? (Reagents and Tools Table, Materials and Methods, Figures, Data Availability Section)
Have **primary datasets** been deposited according to the journal's guidelines (see 'Data Deposition' section) and the respective accession numbers provided in the Data Availability Section?	Yes	Data Availability section
Were **human clinical and genomic datasets** deposited in a public access-controlled repository in accordance to ethical obligations to the patients and to the applicable consent agreement?	Not Applicable	
Are **computational models** that are central and integral to a study available without restrictions in a machine-readable form? Were the relevant accession numbers or links provided?	Not Applicable	
If publicly available data were reused, provide the respective **data citations in the reference list**.	Not Applicable	
